# Stereocontrolled addition of Grignard reagents to oxa-bridged benzazepines: highly efficient synthesis of functionalized benzazepine scaffolds†

**DOI:** 10.1039/d0ra08758k

**Published:** 2020-11-17

**Authors:** Yuewei Zhang, Qingqing Bao, Ning Zhang, Shuohang Wang, Xue Yu

**Affiliations:** School of Chemistry and Pharmaceutical Engineering, Jilin Institute of Chemical Technology Jilin 132022 China dongjibinghuayuxue@163.com

## Abstract

An efficient and highly diastereoselective synthesis of 2-substituted benzo[*b*]azepin-5-ol *via* stereocontrolled addition of Grignard reagents to oxa-bridged benzazepines has been developed. The reaction proceeds efficiently starting from versatile skeletons with mild reaction conditions as well as simple operation. Furthermore, 2-substituted benzazepinones could been obtained by simple Dess–Martin oxidation in excellent yields.

## Abstract 2

An efficient and highly diastereoselective synthesis of 2-substituted benzo[*b*]azepin-5-ol *via* stereocontrolled addition of Grignard reagents to oxa-bridged benzazepines has been developed.

## Graphical abstract

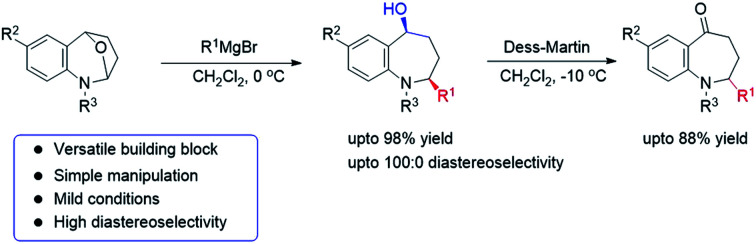

Benzofused azepines, a unique family of seven-member aza-heterocycles, are widely found in numerous bioactive molecules, natural products and pharmaceuticals.^1–4^ This is due to their chemotherapeutic properties, and exhibiting interesting biological activities,^5–9^ for instance, competitive vasopressin receptor antagonist (tolvaptan),^10–12^ antidepressants (mianserin),^13^ zilpaterol (beef improvement agent),^14^ ACE inhibitor (benazepril)^15^ (Fig. 1).

**Fig. 1:**
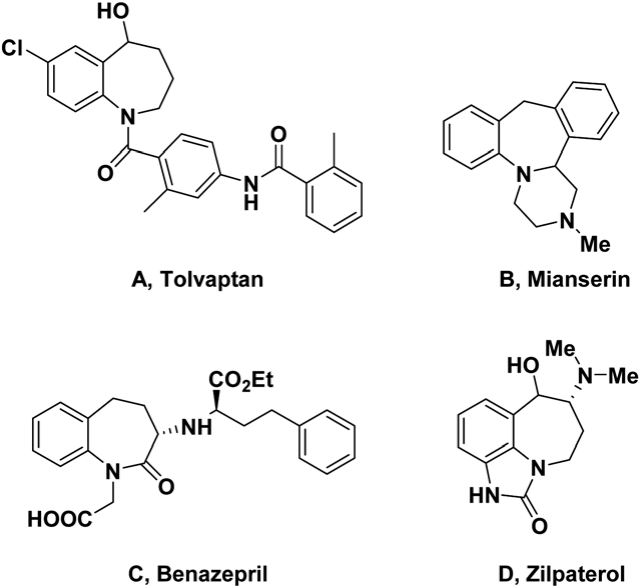
Selected examples containing a benzazepine skeleton.

Consequently, tremendous efforts have recently been dedicated to developing new methodologies to construct the benzazepine derivatives. Typically, the benzazepine skeletons could be assembled by expansion of smaller rings, rearrangements,^16,17^ Dieckmann cyclization,^18,19^ transition-metal-catalyzed coupling, ring closure metathesis,^15,20,21^ and others.^22–25^ Nevertheless, most of these protocols are limited to highly engineered starting materials, expensive catalysts and hazardous handling, obviously expeditious strategies for the diverse construction of benzazepine backbones from readily available starting materials, remains highly attractive and challenging.

Diversity-oriented synthesis (DOS), defined as a powerful synthetic strategy to the libraries of diverse highly valuable molecules from one parent compound,^26,27^ is therefore well-suited for the timely design and execution of parallel (library) synthesis.^28^ In recent years, our group focused on the development of a more facile and efficient diversity-oriented synthesis strategy for the generation of this class of 7-membered heterocyclic compounds.^29–31^ This newly introduced ene-type cyclization reaction was used to prepare a series of bridged aromatic fused azepines,^29^ as a versatile building block, which could be transformed into structurally different ring systems through selective ring opening of the cyclic acetals (Scheme 1A).^30,31^

**Scheme 1:**
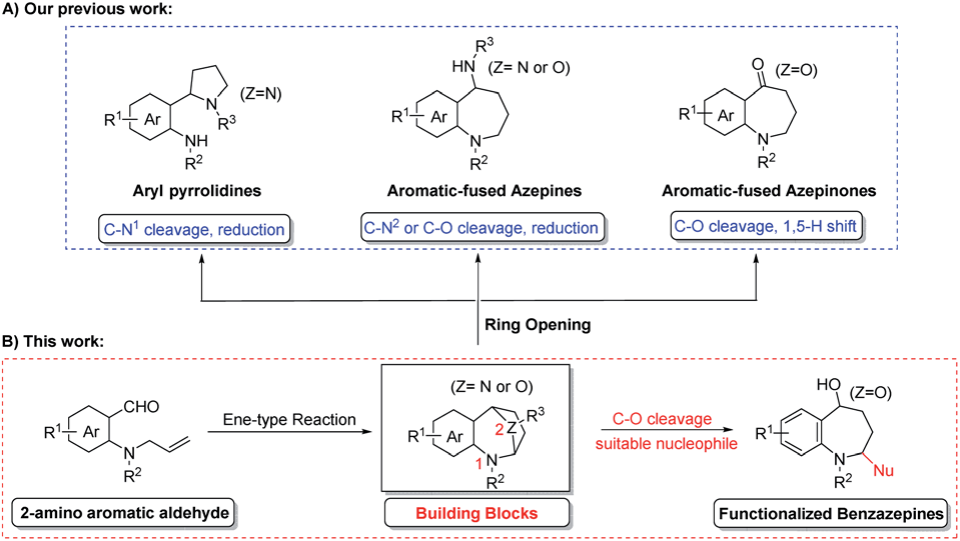
Our previous work (A) and this work (B).

As an extension of our ongoing work toward the synthesis of the azepine skeleton, we suggested a new reaction model could be achieved if the suitable nucleophile could be carefully designed. Recently, a couple of efficient approaches to access nitrogen-containing heterocycles has been developed through the nucleophilic addition of cyclic *N*,*O*-acetal with Grignard reagents.^32–35^ Inspired by their excellent studies and to showcase the utility of cyclic *N*,*O*-acetal building blocks for the preparation of functionalized azepines, we present a facile approach to a stereoselective synthesis of 2,5-substituted benzazepine derivatives from oxa-bridged benzazepines by Grignard addition. This strategy is complementary to our recently published cascade reaction to prepare the benzazepinone scaffold. Herein, the details of this study is disclosed.

Our investigations commenced by exploring nucleophilic addition of 1a, which was readily prepared in two steps *via* substitution reaction and subsequent ene-type reaction (see the ESI†). We started our screening with 1-allyl-2,3,4,5-tetrahydro-1*H*-2,5-epoxybenzo[*b*]azepine (1a) as a model substrate for the optimization of the reaction conditions (Table 1). First, we chose the commonly used solvent tetrahydrofuran and dioxane for Grignard addition, no 2a was observed (Table 1, entries 1 and 2). The desired product 2a was obtained in 92% yield, along with low diastereoselectivity (*dr* = 24 : 76), when the reaction was carried out in diethyl ether (Table 1, entry 3). Subsequently, we conducted this nucleophilic addition of 1a in halogen-containing solvent instead of the more commonly used ether solvent to increase the coordination of organomagnesium to the substrates.^36,37^ We were gratified to find that Grignard reagents could indeed be added with high selectivity (Table 1, entries 4–6).

**Table 1:** Optimization of the Grignard addition conditions^a^

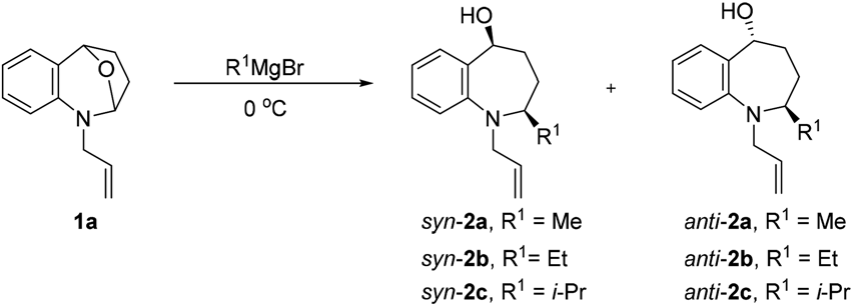					
Entry	Solvent	Additive	Time	Yield^b^ (%)	*dr* (*syn*/*anti*)^c^
1^d^	THF	—	10 h	0	—
2^d^	Dioxane	—	10 h	0	—
3	Et_2_O	—	30 min	92	2a, 24/76
4	1,2-Dichloroethane	—	10 min	90	2a, 68/32
5	CHCl_3_	—	10 min	95	2a, 66/34
6	CH_2_Cl_2_	—	10 min	96	2a, 69/31
7	CH_2_Cl_2_	MgBr_2_ (1.2 equiv.)	10 min	90	2a, 70/30
8	CH_2_Cl_2_	—	10 min	97	2b, 91/9
9	CH_2_Cl_2_	—	10 min	88	2c, 100/0

a Reaction conditions: 1a (1 mmol), MeMgBr in 10 mL of solvent at 0 °C under air.

b Isolated yield after column chromatography.

c Determined by ^1^H NMR and X-ray crystallographic analysis.

d The reaction was conducted at 0 °C for 1 h, then at 25 °C for 9 h.

However, there was no significant improvement in the diastereoselectivity was observed after the addition of magnesium bromide^36^ (Table 1, entry 7). The experimental results show that the ring-opening reaction of *N*,*O* acetals were sensitive to size of substituent R^1^, and the *syn*-selectivity became better as the size of Grignard reagents increased (Table 1, entries 8, 9).

After establishing the optimized reaction conditions, we investigated the addition of Grignard reagents to a series of oxa-bridged azepine 1, and the results are listed in Table 2. We were pleased to observe that this reaction exhibited broad substrate scope, and the aliphatic and aromatic Grignard reagents reacted smoothly with 1a. These reactions generated aminoalcohols 2 in good chemical yields and with excellent selectivity and the structure of 2m was further confirmed by X-ray crystallography analysis. The compound 2m was recrystallized in an ethyl acetate/petroleum ether solution to obtain a single configuration compound *syn*-2m, which was tested by single crystal X-ray diffraction (Fig. 2). From the diffraction pattern, it could be clearly seen that the hydroxyl group and the methyl group are on the same side. The stereochemistry in the rest of the series could be unambiguously assigned by comparison of their NMR spectra with those of *syn*-2m.

**Table 2:** Nucleophilic addition with Grignard reagents on cyclic *N*,*O*-acetals^a^

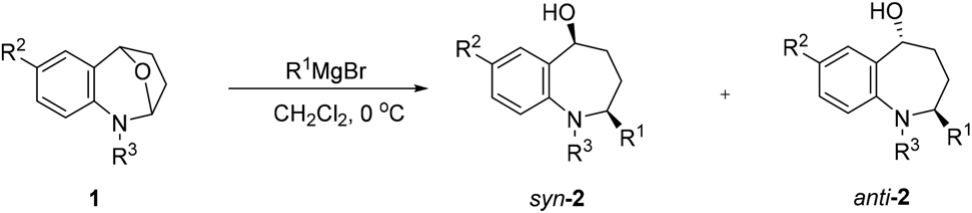								
Entry	*N*,*O*-Acetals	R^1^	R^2^	R^3^	Time	2	Yield (%)	*syn*/*anti*^b^
1	1a	Me	H	Allyl	10 min	2a	96	69/31
2	1a	Et	H	Allyl	10 min	2b	97	91/9
3	1a	*i*-Pr	H	Allyl	7 h	2c	88	100/0
4	1a	Cy	H	Allyl	18 h	2d	83	100/0
5	1a	Allyl	H	Allyl	10 min	2e	98	0/100
6	1a	Ph	H	Allyl	10 min	2f	98	100/0
7	1b	Me	Me	Allyl	10 min	2g	91	88/12
8	1b	Allyl	Me	Allyl	10 min	2h	90	0/100
9	1c	Me	Cl	Allyl	10 min	2i	92	67/33
10	1c	Allyl	Cl	Allyl	10 min	2j	91	0/100
11	1d	Me	H	Me	10 min	2k	92	68/32
12	1d	*i*-Pr	H	Me	5 min	2l	91	100/0
13	1e	Me	H	H	1 h	2m	80	91/9
14	1e	Allyl	H	H	10 min	2n	81	75/25

a Unless indicated otherwise, the reaction was carried out on 1.0 mmol scale in DCM (10 mL).

b Diastereoisomeric ratios were determined by ^1^H NMR analysis of the mixture, see the ESI for details.

**Fig. 2:**
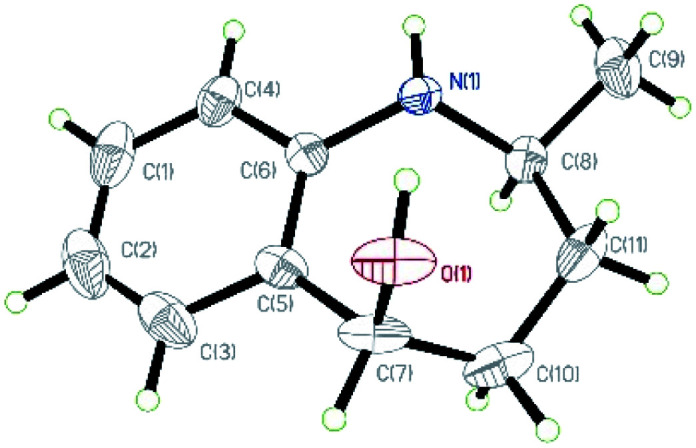
X-ray crystallographic structure of *syn*-2m.

In general, the improved diastereoselectivities were observed with increasing steric bulk of the Grignard reagents, the *syn*-adduct was the major product in all cases (Table 2, entries1-4). The anomalous diastereoselection shown by allyl Grignard reagent is to be underlined. Possibly this is due to the peculiar nature of allyl metals (Table 2, entries 5, 8, 10).^38–43^ Even phenylmagnesium bromide could be added to 1a and provided the adduct in 98% yield and a single *syn*-adduct (Table 2, entry 6), despite the considerably higher basicity of this Grignard reagent.^37^ 7-Substituented substrates (1b, 1c) were also tolerated by this process, as well as Me at the nitrogen atom of the substrate (1d) (Table 1, entries 7–12). To further expand the scope of this reaction, *N*-unsubstituted cyclic *N*,*O*-acetal 1e was employed under the established condition, the expected product was obtained smoothly and in good yield as well. Surprisingly, even lower proportions of *syn*-diastereoisomers were observed relative to methyl Grignard reagent correlated with increasing steric bulk of the Grignard reagent (Table 2, entries 13–14).

The observed diastereoselectivity for the Grignard reaction of *N*-substituted cyclic *N*,*O*-acetals 1 leading to 2 can be rationalized by assuming that the Grignard reagent coordinates with the oxygen atom of the cyclic *N*,*O*-acetal ring 1 and that the subsequent intramolecular delivery of the alkyl group occurs on the same face of the C–O bond of the incipient iminium salt A as shown in Fig. 3 (transition state A).^36,44,45^ However, this diastereoselectivity for Grignard addition to the *N*-unsubstituted cyclic *N*,*O*-acetal 1a may be attributed to a highly ordered transition state resulting from significant chelation of the alkoxy substituent and imino nitrogen to at least one magnesium cation as shown Fig. 3 (transition state B).^46,47^

**Fig. 3:**
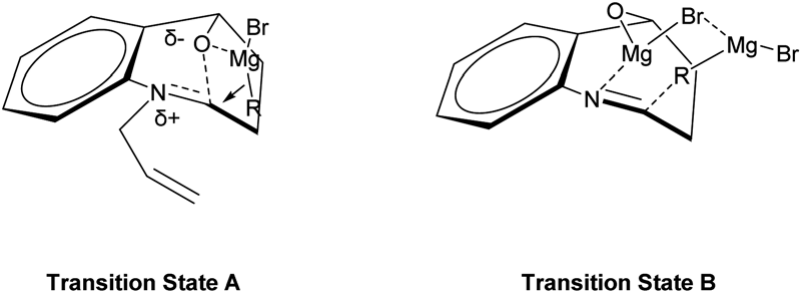
Proposed transition states accounting for the diastereoselectivity.

To give the intrinsic versatility of 2-substituted benzo[*b*]azepin-5-ol and as a complement to our recently published cascade reaction to prepare the benzazepinone scaffold, treatment of the above compounds 2 with Dess–Martin in CH_2_Cl_2_ gave the 2-substituted benzazepinones 3 in good to excellent yields (Table 3, entries 1–10), except the 3k (Table 3, entry 11).

**Table 3:** The synthesis of 2-substituted benzazepinones^a^

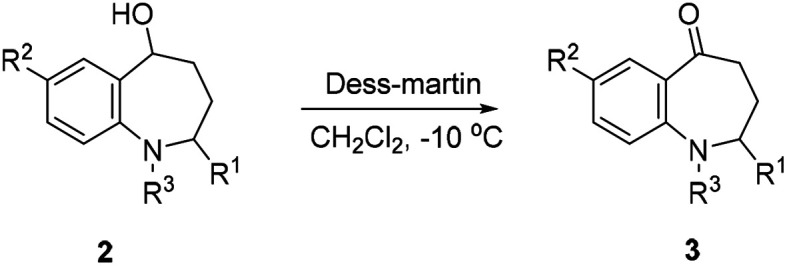						
Entry	R^1^	R^2^	R^3^	Time (min)	3	Yield (%)
1	H	Allyl	Me	30	3a	85
2	H	Allyl	Et	30	3b	83
3	H	Allyl	*i*-Pr	15	3c	86
4	H	Allyl	Cy	15	3d	80
5	H	Allyl	Allyl	15	3e	75
6	H	Allyl	Ph	15	3f	88
7	Me	Allyl	Me	20	3g	88
8	Me	Allyl	Allyl	45	3h	76
9	Cl	Allyl	Me	60	3i	84
10	Cl	Allyl	Allyl	60	3j	78
11	H	H	Me	20	3k	38

a Unless indicated otherwise, the reaction was carried out on 0.5 mmol scale in DCM (5 mL).

The synthetic versatility of 2-substituted benzazepinones has also been explored. The fused tricyclic compound 4 (ref. 48) could also be readily synthesized from 3e. Treatment of 3e with Grubbs II catalyst led to 4 in 95% yield [eqn (1)].1
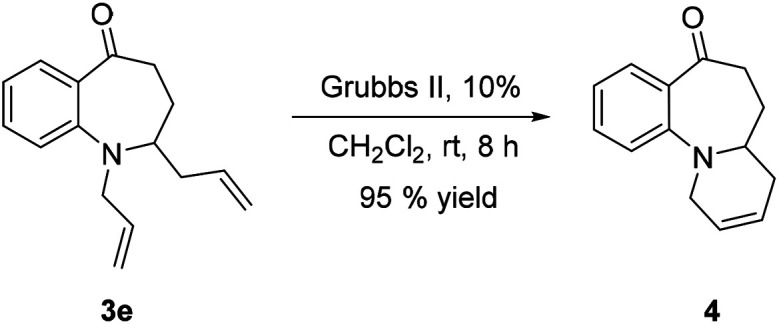


In conclusion, we have demonstrated that the cyclic *N*,*O*-acetals were successfully applied to the diastereoselective addition of various Grignard reagents with encouraging levels of stereoselection. In the formation of 2,5-substituted 1-benzazepine derivatives, the reaction proceeds through a ring-opening/nucleophilic addition pathway. These benzo[*b*]azepin-5-ols then undergo simple Dess–Martin oxidation to afford the 2-substituted benzazepinones in excellent yields. In respect to the easy availability of the starting materials, simple manipulation, mild conditions and high diastereoselectivity, this reaction will be synthetically useful in organic chemistry.

## Conflicts of interest

There are no conflicts to declare.

## Supplementary Material

RA-010-D0RA08758K-s001

RA-010-D0RA08758K-s002

## References

[cit1] Vitaku E., Smith D. T., Njardarson J. T. (2014). J. Med. Chem..

[cit2] Benes J., Parada A., Figueiredo A. A., Alves P. C., Freitas A. P., Learmonth D. A., Cunha R. A., Garrett J., Soares-da-Silva P. (1999). J. Med. Chem..

[cit3] Link A., Kunick C. (1998). J. Med. Chem..

[cit4] Seto M., Miyamoto N., Aikawa K., Aramaki Y., Kanzaki N., Iizawa Y., Baba M., Shiraishi M. (2005). Bioorg. Med. Chem..

[cit5] Schultz C., Link A., Leost M., Zaharevitz D. W., Gussio R., Sausville E. A., Meijer L., Kunick C. (1999). J. Med. Chem..

[cit6] Kunick C., Lauenroth K., Wieking K., Xie X., Schultz C., Gussio R., Zaharevitz D., Leost M., Meijer L., Weber A., Jorgensen F. S., Lemcke T. (2004). J. Med. Chem..

[cit7] Li Z., Lu N., Wang L., Zhang W. (2012). Eur. J. Org. Chem..

[cit8] Palma A., Yépes A. F., Leal S. M., Coronado C. A., Escobar P. (2009). Bioorg. Med. Chem. Lett..

[cit9] Gómez Ayala S. L., Stashenko E., Palma A., Bahsas A., Amaro-Luis J. M. (2006). Synlett.

[cit10] Ogawa H., Yamashita H., Kondo K., Yamamura Y., Miyamoto H., Kan K., Kitano K., Tanaka M., Nakaya K., Nakamura S., Mori T., Tominaga M., Yabuuchi Y. (1996). J. Med. Chem..

[cit11] Kondo K., Ogawa H., Shinohara T., Kurimura M., Tanada Y., Kan K., Yamashita H., Nakamura S., Hirano T., Yamamura Y., Mori T., Tominaga M., Itai A. (2000). J. Med. Chem..

[cit12] Kondo K., Kan K., Tanada Y., Bando M., Shinohara T., Kurimura M., Ogawa H., Nakamura S., Hirano T., Yamamura Y., Kido M., Mori T., Tominaga M. (2002). J. Med. Chem..

[cit13] Roszkowski P., Maurin J. K., Czarnocki Z. (2015). Beilstein J. Org. Chem..

[cit14] Kern C., Meyer T., Droux S., Schollmeyer D., Miculka C. (2009). J. Med. Chem..

[cit15] Kotha S., Shah V. R. (2008). Eur. J. Org. Chem..

[cit16] Cordero-Vargas A., Quiclet-Sire B., Zard S. Z. (2006). Bioorg. Med. Chem..

[cit17] Rickards R. W., Smith R. M. (1966). Tetrahedron Lett..

[cit18] Kondo K., Ogawa H., Yamashita H., Miyamoto H., Tanaka M., Nakaya K., Kitano K., Yamamura Y., Nakamura S., Onogawa T., Mori T., Tominaga M. (1999). Bioorg. Med. Chem..

[cit19] Kawakita Y., Seto M., Ohashi T., Tamura T., Yusa T., Miki H., Iwata H., Kamiguchi H., Tanaka T., Sogabe S., Ohta Y., Ishikawa T. (2013). Bioorg. Med. Chem..

[cit20] Dolman S. J., Schrock R. R., Hoveyda A. H. (2003). Org. Lett..

[cit21] Ghosh D., Thander L., Ghosh S. K., Chattopadhyay S. K. (2008). Synlett.

[cit22] Ohtani T., Kawano Y., Kitano K., Matsubara J., Komatsu M., Uchida M., Tabusa F., Nagao Y. (2005). Heterocycles.

[cit23] Qadir M., Cobb J., Sheldrake P. W., Whittall N., White A. J. P., Hii K. K., Horton P. N., Hursthouse M. B. (2005). J. Org. Chem..

[cit24] Singh V., Batra S. (2007). Eur. J. Org. Chem..

[cit25] He H., Liu W.-B., Dai L.-X., You S.-L. (2010). Angew. Chem., Int. Ed..

[cit26] Elek G. Z., Koppel K., Zubrytski D. M., Konrad N., Järving I., Lopp M., Kananovich D. G. (2019). Org. Lett..

[cit27] Idzik T. J., Myk Z. M., Sośnicki J. G. (2019). J. Org. Chem..

[cit28] Mortensen K. T., Osberger T. J., King T. A., Sore H. F., Spring D. R. (2019). Chem. Rev..

[cit29] Zhang Y., Zhu Y., Zheng L., Zhuo L.-G., Yang F., Dang Q., Yu Z.-X., Bai X. (2014). Eur. J. Org. Chem..

[cit30] Zhang Y., Zheng L., Yang F., Zhang Z., Dang Q., Bai X. (2015). Tetrahedron.

[cit31] Zhang Y., Yang F., Zheng L., Dang Q., Bai X. (2014). Org. Lett..

[cit32] Huang Y.-Y., Cai C., Yang X., Lv Z.-C., Schneider U. (2016). ACS Catal..

[cit33] Rong H.-J., Yao J.-J., Li J.-K., Qu J. (2017). J. Org. Chem..

[cit34] Wang X.-M., Liu Y.-W., Ma R.-J., Si C.-M., Wei B.-G. (2019). J. Org. Chem..

[cit35] Chen W.-L., Wang L.-Y., Li Y.-J. (2020). Eur. J. Org. Chem..

[cit36] Spero D. M., Kapadia S. R. (1997). J. Org. Chem..

[cit37] Steinig A. G., Spero D. M. (1999). J. Org. Chem..

[cit38] Yamamoto Y., Asao N. (1993). Chem. Rev..

[cit39] Nakamura H., Iwama H., Yamamoto Y. (1996). J. Am. Chem. Soc..

[cit40] Nakamura M., Hirai A., Nakamura E. (1996). J. Am. Chem. Soc..

[cit41] Bloch R. (1998). Chem. Rev..

[cit42] Bonanni M., Marradi M., Cicchi S., Faggi C., Goti A. (2004). Org. Lett..

[cit43] Kuduk S. D., DiPardo R. M., Chang R. K., Ng C., Bock M. G. (2004). Tetrahedron Lett..

[cit44] Higashiyama K., Inoue H., Takahashi H. (1994). Tetrahedron.

[cit45] Andrés C., Nieto J., Pedrosa R., Villamañán N. (1996). J. Org. Chem..

[cit46] Wu M. J., Pridgen L. N. (1991). J. Org. Chem..

[cit47] Ulrich Veith S. L. a. V. J. (1996). Chem. Commun..

[cit48] Pearson W. H., Fang W.-k. (2000). J. Org. Chem..

