# Supplementary material for: Stereocontrolled addition of Grignard reagents to oxa-bridged benzazepines: highly efficient synthesis of functionalized benzazepine scaffolds
Source: RSC Adv. 2020 Nov 17;10(68):41802–6. doi: 10.1039/d0ra08758k (PMC9057913; doi:10.1039/d0ra08758k)
Supplement: RA-010-D0RA08758K-s001 [file RA-010-D0RA08758K-s001.pdf]

Electronic Supplementary Material (ESI) for RSC Advances.
This journal is © The Royal Society of Chemistry 2020

Supporting Information

For

Stereocontrolled Addition of Grignard Reagents to Oxa-Bridged Benzazepines: Highly Efficient Synthesis of Functionalized Benzazepine Scaffolds

Yuewei Zhang [a], Qingqing Bao [a], Ning Zhang [a], Shuohang Wang [a], Xue Yu [b*]

([a] School of chemistry and pharmaceutical engineering, Jilin institute of chemical technology, Jilin 132022, China;
[b] Division of science and technology, Jilin institute of chemical technology, Jilin 132022, China;)

Table of Contents

General Experimental Procedures. 2

General Procedure for the Synthesis of **S2a**, **S2c**, **S2d** 3

2-(Diallylamino)-5-methylbenzaldehyde **S2b** 4

General Procedure for the Synthesis of **1a**, **1c**, **1d** 5

1-Allyl-7-methyl-2,3,4,5-tetrahydro-*1H*-2,5-epoxybenzo[*b*]azepine **1b** 6

2,3,4,5-tetrahydro-*1H*-2,5-epoxybenzo[*b*]azepine **1e** 8

General Procedure for the Synthesis of **2** 9

General Procedure for the Synthesis of **3** 19

Procedure for the Synthesis of **4** 26

NMR Spectra 28

X-ray Crystallographic data for Compound syn-**2m** 65

Confirmation of the ratio of stereoisomers **2** 65

General Experimental Procedures. Unless otherwise noted, reactions were carried out in air. Chemicals and solvents were either purchased from commercial suppliers or purified by standard procedures. Analytical thin-layer chromatography (TLC) was performed on silica gel plates with F-254 indicator and compounds were visualized by irradiation with UV light or by treatment with iodine. Flash column chromatography was performed with silica gel 200-300 mesh. The melting point was uncorrected. Mass spectra and HPLC data were recorded on a LC/MS system. ^{1}H NMR and ^{13}C NMR spectra were recorded on 300 MHz and 75 MHz spectrometers respectively. The spectra were recorded in $CDCl_3$ as the solvent at room temperature unless otherwise noted. ^{1}H and ^{13}C NMR chemical shifts are reported in ppm relative to TMS as an internal standard. Multiplicities are indicated as follows: s, singlet; d, doublet; t, triplet; q, quartet; m, multiplet; dd, doubled doublet; br, broad. HRMS was recorded on an LC TOF (ES).

Displacement of the 2-fluorobenzaldehyde **S1** using allylamine provided 2-(allylamino)benzaldehyde **S2**. Aldehyde **S2** was transformed into oxa-bridged azepine **1** by the carbonyl ene-type reaction in excellent yields. Subsequently, removal of the allyl group in **1a** with a palladium catalyst furnished amine **1e**, as shown in S-Scheme 1.

S-Scheme 1. Synthesis of Ring-Opening Precursors

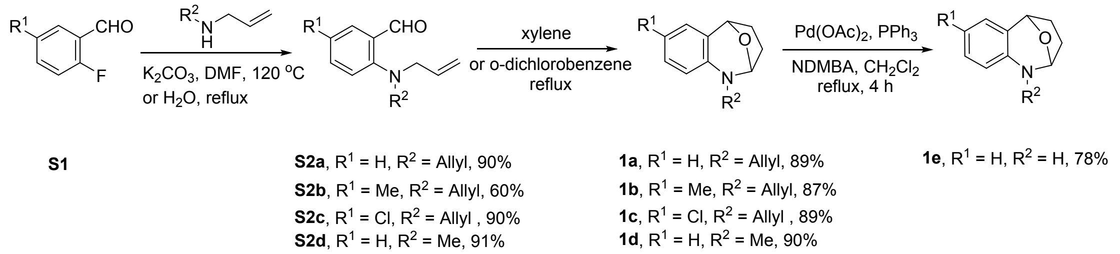

S1

S2a, R^1 = H, R^2 = Allyl, 90%
S2b, R^1 = Me, R^2 = Allyl, 60%
S2c, R^1 = Cl, R^2 = Allyl , 90%
S2d, R^1 = H, R^2 = Me, 91%

1a, R^1 = H, R^2 = Allyl, 89%
1b, R^1 = Me, R^2 = Allyl, 87%
1c, R^1 = Cl, R^2 = Allyl , 89%
1d, R^1 = H, R^2 = Me, 90%

1e, R^1 = H, R^2 = H, 78%

General Procedure for the Synthesis of S2a, S2c, S2d

To a solution of 2-fluorobenzaldehyde (248 mg, 2 mmol) in water (4 mL) was added secondary amine (2.4 mmol) and K_2CO_3 (0.414 mg, 3 mmol). The reaction mixture was heated at reflux until 2-fluorobenzaldehyde was consumed as monitored by TLC. The reaction solution was then diluted with water (10 mL), and extracted with EtOAc (3 × 15 mL). The combined organic extracts were washed with brine, dried over anhydrous Na_2SO_4 and concentrated *in vacuo*. Purification by flash column chromatography (petroleum ether/ethyl acetate = 50:1, v/v) afforded the desired product S**2**.

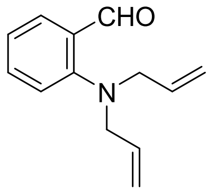

2-(Diallylamino)benzaldehyde (S2a)

Pale yellow oil (362 mg, 90% yield); ^{1}H NMR (300 MHz, $CDCl_3$) δ 10.35 (s, 1H, CHO), 7.80 (dd, J = 7.8, 1.8 Hz, 1H, phenyl-H), 7.55 – 7.36 (m, 1H, phenyl-H), 7.20 – 6.95 (m, 2H, phenyl-H), 5.95 – 5.70 (m, 2H, $CH_2C\mathit{H}{=}CH_2$), 5.32 – 5.12 (m, 4H, $2CH_2CH{=}C\mathit{H_2}$), 3.89 – 3.71 (m, 4H, $2C\mathit{H_2}CH{=}CH_2$); ^{13}C NMR (75 MHz, $CDCl_3$) δ 191.4, 154.1, 134.1, 133.8, 129.3, 129.2, 122.0, 121.2, 118.0, 57.18. HRMS (ESI-TOF): calcd. for $C_{13}H_{16}NO$ $[M + H]^+$ 202.1226; found 202.1223.

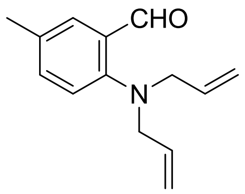

2-(Diallylamino)-5-methylbenzaldehyde (S2b)

To a solution of 2-fluoro-5-methylbenzaldehyde (276 mg, 2 mmol) in *N,N*-Dimethylformamide (4 mL) was added diallylamine (0.49 mL, 4 mmol) and K_2CO_3 (0.414 mg, 3 mmol). The reaction mixture was heated at reflux 4 d. The reaction solution was then diluted with water (40 mL), and extracted with EtOAc (3 × 15 mL). The combined organic extracts were washed with brine, dried over anhydrous Na_2SO_4 and concentrated *in vacuo*. Purification by flash column chromatography (petroleum ether/ethyl acetate = 30:1, v/v) afforded the desired product **2b** (258 mg, 60%) as a pale yellow oil. ^{1}H NMR (300 MHz, $CDCl_3$) δ 10.38 (s, 1H, CHO), 7.61 (d, J = 2.4 Hz, 1H, phenyl-H), 7.29 (ddd, J = 8.4, 2.4, 0.6 Hz, 1H, phenyl-H), 7.03 (d, J = 8.4 Hz, 1H, phenyl-H), 5.94 – 5.74 (m, 2H, $CH_2CH{=}CH_2$), 5.23 – 5.13 (m, 4H, $CH_2CH{=}CH_2$), 3.73 (dt, J = 6.0, 1.2 Hz, 4H, $CH_2CH{=}CH_2$), 2.32 (s, 3H, CH_3); ^{13}C NMR (75 MHz, $CDCl_3$) δ 191.9, 152.1, 135.1, 134.0, 132.1, 129.6, 129.1, 121.7, 117.9, 57.5, 20.4; HRMS (ESI-TOF): calcd. for $C_{14}H_{18}NO$ $[M + H]^+$ 216.1383; found 216.1389.

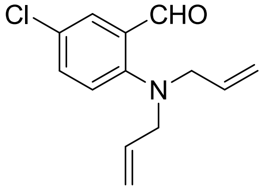

5-Chloro-2-(diallylamino)benzaldehyde (S2c)

Pale yellow oil (635 mg, 90% yield). ^{1}H NMR (300 MHz, $CDCl_3$) δ 10.29 (s, 1H, CHO), 7.75 (d, J = 2.7 Hz, 1H, phenyl-H), 7.40 (dd, J = 8.7, 2.7 Hz, 1H, phenyl-H), 7.05 (d, J = 8.7 Hz, 2H, phenyl-H), 5.93 – 5.66 (m, 2H, $2CH_2CH{=}CH_2$), 5.36 – 5.08

(m, 4H, $2CH_2CH{=}C\mathit{H_2}$), 3.76 (dt, J = 6.0, 1.2 Hz, 4H, $2C\mathit{H_2}CH{=}CH_2$); ^{13}C NMR (75 MHz, $CDCl_3$) δ 190.1, 152.5, 133.9, 133.4, 130.2, 128.7, 127.8, 122.9, 118.4, 57.3; HRMS (ESI-TOF): calcd. for $C_{13}H_{15}ClNO$ $[M + H]^+$ 236.0837; found 236.0841.

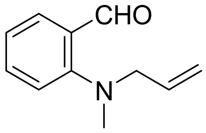

2-(Allyl(methyl)amino)benzaldehyde (S2d).

Yellow oil (319 mg, 91% yield); ^{1}H NMR ($CDCl_3$) δ 10.26 (s, 1H, CHO), 7.78 (dd, J = 7.7, 1.6 Hz, 1H, phenyl-H), 7.55 – 7.38 (m, 1H, phenyl-H), 7.08 (d, J = 8.4 Hz, 1H, phenyl-H), 7.06 – 6.96 (m, 1H, phenyl-H), 5.91 (m, 1H, $CH_2C\mathit{H}{=}CH_2$), 5.26 (m, 2H, $CH_2CH{=}C\mathit{H_2}$), 3.73 (d, J = 5.7 Hz, 2H, $\mathit{CH_2}CH{=}CH_2$), 2.85 (s, 3H, CH_3); ^{13}C NMR (75 MHz, $CDCl_3$) δ 191.2, 155.4, 134.4, 134.0, 130.1, 127.7, 121.1, 118.8, 117.8, 61.9, 40.9; HRMS (ESI-TOF): calcd. for $C_{11}H_{14}NO$ $[M + H]^+$ 176.1070; found 176.1078.

General Procedure for the Synthesis of 1a, 1c, 1d

The 2-(allylamino)nicotinaldehyde **S2** (1.0 mmol) was dissolved in xylene (10 mL). The resulting solution was stirred at reflux until **S2** had been consumed, as monitored by TLC. The reaction mixture was purified by flash column chromatography (petroleum ether/ethyl acetate, 30:1, v/v) afforded the desired product **1**.

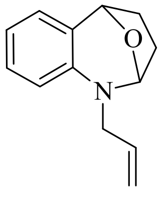

1-Allyl-2,3,4,5-tetrahydro-*1H*-2,5-epoxybenzo[*b*]azepine (1a).

Pale yellow oil (179 mg, 89% yield); ^{1}H NMR (300 MHz, $CDCl_3$) δ 7.09 (t, J = 7.5 Hz, 1H, phenyl-H), 6.89 (d, J = 7.2 Hz, 1H, phenyl-H), 6.73 – 6.60 (m, 2H, phenyl-H), 6.08 – 5.80 (m, 1H, CH_2CH=CH_2), 5.42 – 4.92 (m, 4H, CH_2CH=$C\textit{H}_2$ and 2CH), 4.10 – 3.70 (m, 2H, $C\textit{H}_2CH$=CH_2), 2.30 – 1.94 (m, 4H, $2CH_2$); ^{13}C NMR (75 MHz, $CDCl_3$) δ 141.8, 134.8, 128.2, 128.0, 124.2, 117.7, 116.3, 114.3, 90.3, 89.8, 77.9, 54.4, 36.8, 33.7. HRMS (ESI-TOF): calcd. for $C_{13}H_{16}NO$ $[M + H]^+$ 202.1226; found 202.1231.

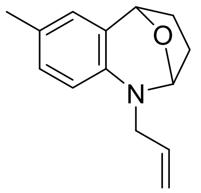

1-Allyl-7-methyl-2,3,4,5-tetrahydro-*1H*-2,5-epoxybenzo[*b*]azepine (1b)

The 2-(diallylamino)-5-methylbenzaldehyde **S2b** (215 mg, 1.0 mmol) was dissolved in orthodichlorobenzene (10 mL). The resulting solution was stirred at reflux 18 h. The reaction mixture was purified by flash column chromatography (petroleum ether/ethyl acetate, 20:1, v/v) afforded the desired product **1b** (187 mg, 87%) as a pale yellow oil. ^{1}H NMR (300 MHz, $CDCl_3$) δ 6.92 (dd, J = 8.1, 2.1 Hz, 1H, phenyl-H), 6.72 (d, J = 2.1 Hz, 1H, phenyl-H), 6.61 (d, J = 8.1 Hz, 1H, phenyl-H), 6.04 – 5.85 (m, 1H, $CH_2C\textit{H}$=CH_2), 5.35 – 5.24 (m, 1H, CH_2CH=$C\textit{H}_2$), 5.23 – 5.13 (m, 2H, CH_2CH=$C\textit{H}_2$ and CH), 5.00 – 4.92 (m, 1H, CH), 3.94 (ddt, J = 16.5, 6.0, 1.5 Hz, 1H, $C\textit{H}_2CH$=CH_2), 3.78 (ddt, J = 16.5, 4.8, 1.8 Hz, 1H, $C\textit{H}_2CH$=CH_2), 2.23 (s, 3H, CH_3), 2.19 – 1.93 (m, 4H, $2CH_2$); ^{13}C NMR (75 MHz, $CDCl_3$) δ 139.7, 135.3, 128.8, 128.5, 127.5, 124.9, 116.4, 115.6, 89.8, 77.6, 55.6, 37.0, 33.1, 20.4; MS (ESI) m/z 216.2 [M

+ H]+. HRMS (ESI-TOF): calcd. for $C_{14}H_{18}NO$ [M + H]+ 216.1383; found 216.1381.

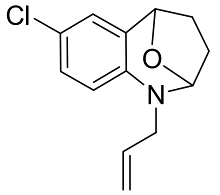

1-Allyl-7-chloro-2,3,4,5-tetrahydro-*1H*-2,5-epoxybenzo[*b*]azepine (1c)

Pale yellow oil (209 mg, 89% yield). ^{1}H NMR (300 MHz, $CDCl_3$) δ 7.04 (dd, J = 8.7, 2.4 Hz, 1H, phenyl-H), 6.87 (d, J = 2.4 Hz, 1H, phenyl-H), 6.54 (d, J = 8.7 Hz, 1H, phenyl-H), 5.96 – 5.80 (m, 1H, CH_2CH=CH_2), 5.32 – 5.23 (m, 1H, CH_2CH=CH_2), 5.22 – 5.12 (m, 2H, CH_2CH=CH_2 and CH), 5.02 – 4.95 (m, 1H, CH_2CH=CH_2), 3.93 (ddt, J = 16.8, 5.7, 1.5 Hz, 1H, CH_2CH=CH_2), 3.79 (ddt, J = 16.8, 4.8, 1.8 Hz, 1H, CH_2CH=CH_2), 2.25 – 2.08 (m, 3H, CH_2), 2.08 – 1.97 (m, 1H, CH_2); ^{13}C NMR (75 MHz, $CDCl_3$) δ 140.4, 134.2, 129.5, 127.8, 124.1, 122.3, 116.6, 115.4, 89.8, 77.5, 54.3, 36.6, 33.9; HRMS (ESI-TOF): calcd. for $C_{13}H_{15}ClNO$ [M + H]+ 236.0837; found 236.0839.

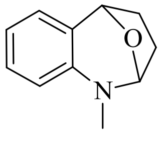

1-Methyl-2,3,4,5-tetrahydro-*1H*-2,5-epoxybenzo[*b*]azepine (1d)

Pale yellow oil (157 mg, 90% yield); ^{1}H NMR (300 MHz, $CDCl_3$) δ 7.18 – 7.04 (m, 1H, phenyl-H), 6.92 – 6.79 (m, 1H, phenyl-H), 6.75 – 6.56 (m, 2H, phenyl-H), 5.14 (d, J = 4.5 Hz, 1H, CH), 5.01 (d, J = 5.4 Hz, 1H, CH), 2.91 (s, 3H, CH_3), 2.26 – 1.99 (m, 4H, 2CH_2); ^{13}C NMR (75 MHz, $CDCl_3$) δ 142.8, 128.1, 124.0, 117.7, 113.9, 91.3,

78.1, 37.5, 36.8, 32.4; HRMS (ESI-TOF): calcd. for $C_{11}H_{14}NO$ $[M + H]^+$ 176.1070; found 176.1076.

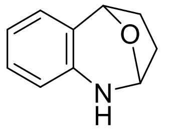

2,3,4,5-tetrahydro-*1H*-2,5-epoxybenzo[*b*]azepine (1e)

To a solution of compound **1a** (402 mg, 2.0 mmol) in dry CH_2Cl_2 (20 mL) was added *N,N'*-Dimethylbarbituric acid (936 mg, 6.0 mmol), palladium acetate (9 mg, 0.04 mmol) and triphenylphosphine (47 mg, 0.18 mmol) under argon. The reaction mixture was stirred at 35 ^oC for 4 h. After cooling, saturated sodium bicarbonate solution was added in the reaction mixture to adjust the pH to 9-10 and extracted with CH_2Cl_2 (3 × 20 mL). The combined organic extracts were washed with brine, dried over anhydrous Na_2SO_4 and concentrated *in vacuo*. Purification by flash column chromatography (petroleum ether/ethyl acetate = 5:1, v/v) afforded the desired product **1e** (251 mg, 78% yield) as a white solid, M.P. 70-72 ^oC. ^1H NMR (300 MHz, $CDCl_3$) δ 7.15 – 7.04 (m, 1H, phenyl-H), 6.95 – 6.91 (m, 1H, phenyl-H), 6.83 – 6.71 (m, 1H, phenyl-H), 6.66 – 6.61 (m, 1H, phenyl-H), 5.38 – 5.35 (m, 1H, CH), 5.04 – 5.01 (m, 1H, CH), 2.32 – 2.14 (m, 3H, CH_2), 2.07 – 2.00 (m, 1H, CH_2); ^{13}C NMR (75 MHz, $CDCl_3$) δ 139.1, 129.1, 127.8, 124.7, 119.2, 117.1, 84.4, 76.8, 37.1, 34.2; HRMS (ESI-TOF): calcd. for $C_{10}H_{12}NO$ $[M + H]^+$ 162.0913; found 162.0918.

General Procedure for the Synthesis of 2

To a solution of compound **1** (1.0 mmol) in dry CH_2Cl_2 (10 mL) at 0 °C was added

Grignard reagent (2.5 mmol). The resulting solution was stirred at 0 °C until compound **1** had been consumed, as monitored by TLC. Then it was treated with saturated aqueous NH_4Cl (5 mL) and extracted with CH_2Cl_2 (3 × 20 mL). The combined organic extracts were washed with brine, dried over anhydrous Na_2SO_4 and concentrated *in vacuo*. Purification by flash column chromatography (petroleum ether/ethyl acetate = 20:1, v/v) afforded the desired product **2**.

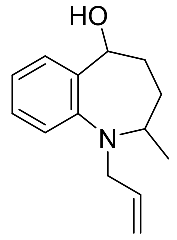

1-Allyl-2-methyl-2,3,4,5-tetrahydro-*1H*-benzo[*b*]azepin-5-ol (2a, syn/anti = 69/31)

Colourless oil (208 mg, 96%);

The major isomer syn-2a was obtained by preparative thin layer chromatography. ^{1}H NMR (300 MHz, $CDCl_3$) δ 7.41 (d, J = 7.5 Hz, 1H, phenyl-H), 7.19 (td, J = 7.5, 1.5 Hz, 1H, phenyl-H), 7.05 (t, J = 7.8 Hz, 1H, phenyl-H), 6.93 (d, J = 7.5 Hz, 1H, phenyl-H), 5.92 – 5.75 (m, 1H, $CH_2CH{=}CH_2$), 5.29 – 5.19 (m, 1H, $CH_2CH{=}CH_2$), 5.14 – 5.08 (m, 1H, $CH_2CH{=}CH_2$), 4.99 (dd, J = 8.1, 4.8 Hz, 1H, C*H* (HO)), 3.91 – 3.80 (m, 1H, $CH_2CH{=}CH_2$), 3.73 – 3.62 (m, 1H, $CH_2CH{=}CH_2$), 3.37 – 3.20 (m, 1H, C*H*(Me)), 2.55 (br. s, 1H, OH), 2.14 – 1.90 (m, 1H, CH_2), 1.87 – 1.55 (m, 2H, CH_2), 1.51 – 1.34 (m, 1H, CH_2), 0.81 (d, J = 6.6 Hz, 3H, CH_3); ^{13}C NMR (75 MHz, $CDCl_3$) δ 145.2, 139.3, 136.7, 126.8, 123.6, 122.5, 121.1, 116.7, 71.5, 54.7, 53.0, 32.6, 30.6, 15.2; HRMS (ESI-TOF): calcd. for $C_{14}H_{20}NO$ [M + H]$^+$ 218.1539; found 218.1547.

The minor isomer anti-2a was obtained by preparative thin layer chromatography. ^{1}H NMR (300 MHz, $CDCl_3$) δ 7.21 – 7.15 (m, 1H, phenyl-H), 7.13 – 7.08 (m, 1H, phenyl-H), 7.03 – 6.90 (m, 2H, phenyl-H), 5.96 – 5.79 (m, 1H, CH_2CH=CH_2), 5.32 – 5.24 (m, 1H, CH_2CH=CH_2), 5.22 – 5.13 (m, 1H, CH_2CH=CH_2), 4.79 (br. s, 1H, OH), 4.71 (t, J = 4.5 Hz, 1H, C*H* (HO)), 3.95 – 3.77 (m, 1H, CH_2CH=CH_2), 3.70 – 3.60 (m, 1H, CH_2CH=CH_2), 3.47 – 3.35 (m, 1H, C*H*(Me)), 2.23 – 2.01 (m, 1H, CH_2), 1.98 – 1.85 (m, 2H, CH_2), 1.42 – 1.30 (m, 1H, CH_2), 0.79 (d, J = 6.9 Hz, 3H); HRMS (ESI-TOF): calcd. for $C_{14}H_{20}NO$ $[M + H]^+$ 218.1539; found 218.1545.

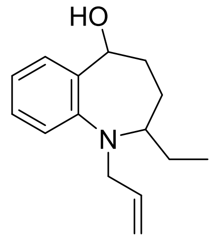

1-Allyl-2-ethyl-2,3,4,5-tetrahydro-*1H*-benzo[*b*]azepin-5-ol (2b, syn/anti = 91/9)

Colourless oil (224 mg, 97%); ^{1}H NMR (300 MHz, $CDCl_3$) δ 7.44 (d, J = 7.2 Hz, 1H, phenyl-H), 7.22 – 7.13 (m, 1.1H, phenyl-H), 7.12 – 7.07 (m, 0.1H, phenyl-H), 7.07 – 7.00 (m, 1H, phenyl-H), 6.99 – 6.91 (m, 1.3H, phenyl-H), 5.98 – 5.74 (m, 1H, CH_2CH=CH_2), 5.34 – 5.18 (m, 1.2H, CH_2CH=CH_2), 5.18–5.05 (m, 1.1H, CH_2CH=CH_2), 4.89 (dd, J = 9.6, 3.6 Hz, 1H, C*H* (HO)), 4.71 (d, J = 4.5 Hz, 0.1H, C*H* (HO)), 4.00 – 3.83 (m, 1.2H, CH_2CH=CH_2), 3.82 – 3.65 (m, 1.3H, CH_2CH=CH_2), 3.18 – 2.94 (m, 1.1H, C*H*(Et)), 2.29 (br. s, 1H, OH), 2.05 – 1.88 (m, 1.2H, CH_2), 1.85 – 1.55 (m, 3H, CH_2), 1.38 – 1.20 (m, 1.2H, CH_2), 1.05 – 0.85 (m, 1.3H, CH_2), 0.85 –

0.70 (m, 3.3H, CH_3); ^{13}C NMR (75 MHz, $CDCl_3$) δ 146.6, 145.7, 139.1, 136.9, 136.0, 127.6, 127.5, 126.6, 123.5, 122.6, 122.1, 121.0, 120.9, 117.6, 116.6, 74.9, 71.9, 60.2, 59.0, 56.0, 55.7, 30.3, 29.2, 27.5, 25.5, 22.5, 21.3, 11.7, 11.5; HRMS (ESI-TOF): calcd. for $C_{15}H_{22}NO$ $[M + H]^+$ 232.1696; found 232.1694.

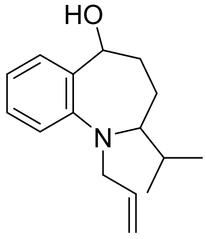

1-Allyl-2-isopropyl-2,3,4,5-tetrahydro-*1H*-benzo[*b*]azepin-5-ol (2c, syn)

Colourless oil (216 mg, 88%); ^1H NMR (300 MHz, $CDCl_3$) δ 7.48 (d, J = 7.5 Hz, 1H, phenyl-H), 7.17 (td, J = 7.5, 1.2 Hz, 1H, phenyl-H), 7.06 – 6.95 (m, 2H, phenyl-H), 5.90 – 5.73(m, 1H, $CH_2CH{=}CH_2$), 5.24 – 5.15 (m, 1H, $CH_2CH{=}CH_2$), 5.11 – 5.05 (m, 1H, $CH_2CH{=}CH_2$), 4.90 – 4.78 (m, 1H, C*H* (HO)), 4.11 – 3.97 (m, 1H, $CH_2CH{=}CH_2$), 3.90 – 3.78 (m, 1H, $CH_2CH{=}CH_2$), 2.93 – 2.83 (m, 1H, C*H*(*i*-Pr)), 2.08–1.92 (m, 1H, CH_2), 1.88 (br. s, 1H, OH), 1.82 – 1.40 (m, 4H, CH_2 and CH), 0.83 (d, J = 6.6 Hz, 3H), 0.69 (d, J = 6.9 Hz, 3H); ^{13}C NMR (75 MHz, $CDCl_3$) δ 145.7, 139.1, 137.2, 126.5, 123.4, 121.9, 121.7, 116.4, 72.0, 63.8, 57.7, 31.0, 28.9, 27.6, 20.7, 19.2; HRMS (ESI-TOF): calcd. for $C_{16}H_{24}NO$ $[M + H]^+$ 246.1852; found 246.1857.

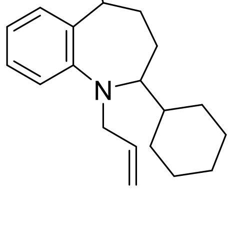

1-Allyl-2-cyclohexyl-2,3,4,5-tetrahydro-*1H*-benzo[*b*]azepin-5-ol (2d, syn)

Colourless oil (237 mg, 83%); ^{1}H NMR (300 MHz, $CDCl_3$) δ 7.48 (d, J = 7.5 Hz, 1H, phenyl-H), 7.17 (td, J = 7.5, 1.5 Hz, 1H, phenyl-H), 7.07 – 6.94 (m, 2H, phenyl-H), 5.90–5.68 (m, 1H, $CH_2CH{=}CH_2$), 5.27 – 5.15 (m, 1H, $CH_2CH{=}CH_2$), 5.12 – 5.03 (m, 1H, $CH_2CH{=}CH_2$), 4.82 (dd, J = 10.2, 3.3 Hz, 1H, C*H* (HO)), 4.05 (ddt, J = 14.1, 5.1, 1.5 Hz, 1H, $CH_2CH{=}CH_2$), 3.90 – 3.72 (m, 1H, $CH_2CH{=}CH_2$), 3.00 – 2.79 (m, 1H, C*H*(Cy)), 2.06 – 1.37 (m, 9H, CH and CH_2), 1.35 – 0.69 (m, 6H, CH_2); ^{13}C NMR (75 MHz, $CDCl_3$) δ 145.7, 139.2, 137.3, 126.5, 123.4, 121.8, 121.7, 116.4, 72.2, 62.5, 58.0, 38.4, 31.0, 29.4, 27.8, 26.4. 26.03, 25.91; MS (ESI) *m/z* 286.1 $[M + H]^+$. HRMS (ESI-TOF): calcd. for $C_{19}H_{28}NO$ $[M + H]^+$ 286.2165; found 286.2170.

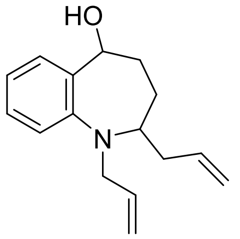

1,2-Diallyl-2,3,4,5-tetrahydro-1H-benzo[b]azepin-5-ol (2e, anti)

Colourless oil (238 mg, 98%); ^{1}H NMR (300 MHz, $CDCl_3$) δ 7.22 – 7.14 (m, 1H, phenyl-H), 7.14 – 7.08 (m, 1H, phenyl-H), 7.02 – 6.93 (m, 2H, phenyl-H), 6.00 – 5.80 (m, 1H, $NCH_2CH{=}CH_2$), 5.75 – 5.60 (m, 1H, $CH_2CH{=}CH_2$), 5.32 – 5.23 (m, 1H, $NCH_2CH{=}CH_2$), 5.23 – 5.16 (m, 1H, $NCH_2CH{=}CH_2$), 5.02 – 4.87 (m, 2H, $CH_2CH{=}CH_2$), 4.76 – 4.66 (m, 1H, C*H* (HO)), 4.46 (br. s, 1H, OH), 3.92 – 3.82 (m, 1H, $NCH_2CH{=}CH_2$), 3.77 – 3.67 (m, 1H, $NCH_2CH{=}CH_2$), 3.34 – 3.24 (m, 1H, C*H*(Me)), 2.22 – 2.02 (m, 2H, CH_2), 1.90 (m, 2H, CH_2), 1.78 – 1.50 (m, 2H, CH_2); ^{13}C NMR (75 MHz, $CDCl_3$) δ 146.3, 138.1, 136.1, 135.8, 127.62, 127.59, 122.9,

121.3, 117.9, 116.8, 74.8, 58.2, 55.5, 33.2, 27.7, 26.0; HRMS (ESI-TOF): calcd. for $C_{16}H_{22}NO$ $[M + H]^+$ 244.1696; found 244.1695.

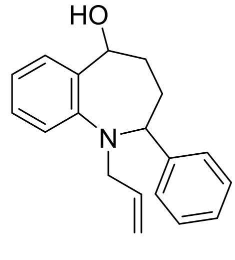

1-Allyl-2-phenyl-2,3,4,5-tetrahydro-*1H*-benzo[*b*]azepin-5-ol (2f, syn)

Colourless oil (273 mg, 98%); ^{1}H NMR (300 MHz, $CDCl_3$) δ 7.43 (d, J = 6.9 Hz, 1H, phenyl-H), 7.38 – 7.19 (m, 4H, phenyl-H), 7.18 – 7.06(m, 3H, phenyl-H), 6.95 (d, J = 7.8 Hz, 1H, phenyl-H), 5.86 – 5.62 (m, 1H, CH_2CH=CH_2), 5.36 (t, J = 8.1 Hz, 1H, C*H* (HO)), 5.14 – 4.95 (m, 2H, CH_2CH=CH_2), 4.22 (dd, J = 10.2, 3.6 Hz, 1H, C*H*(Ph)), 3.72 – 3.55 (m, 1H, CH_2CH=CH_2), 3.48 – 3.35 (m, 1H, CH_2CH=CH_2), 2.61 – 2.19 (m, 2H, OH and CH_2), 1.85 – 1.47 (m, 3H, CH_2); ^{13}C NMR (75 MHz, $CDCl_3$) δ 144.7, 144.3, 138.7, 135.7, 128.3, 127.24, 127.15, 126.9, 123.4, 122.7, 121.9, 116.9, 69.8, 63.9, 54.0, 32.7, 32.4; HRMS (ESI-TOF): calcd. for $C_{19}H_{22}NO$ $[M + H]^+$ 280.1696; found 280.1699.

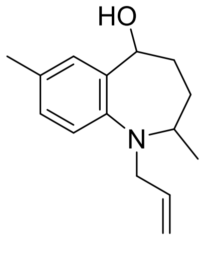

1-Allyl-2,7-dimethyl-2,3,4,5-tetrahydro-*1H*-benzo[*b*]azepin-5-ol (2g, syn/anti = 88/12)

Colourless oil (210 mg, 91%); ^{1}H NMR (300 MHz, $CDCl_3$) δ 7.21 (br. s, 1H, phenyl-

H), 7.05 – 6.95 (m, 1H, phenyl-H), 6.91 (d, *J* = 2.1 Hz, 0.2H, phenyl-H), 6.88 – 6.75 (m, 1H, phenyl-H), 5.98 – 5.70 (m, 1H, $CH_2CH{=}CH_2$), 5.34 – 5.16 (m, 1.1H, $CH_2CH{=}CH_2$), 5.16 – 4.94 (m, 1H, $CH_2CH{=}CH_2$), 4.98 (dd, *J* = 7.8, 4.8 Hz, 1H, C*H* (HO)), 4.64 (t, *J* = 4.5 Hz, 0.2H, C*H* (HO)), 3.90 – 3.50 (m, 2.3H, $CH_2CH{=}CH_2$), 3.42 – 3.33 (m, 0.17H, $CH(CH_3)$), 3.33 – 3.18 (m, 1H, $CH(CH_3)$), 2.69 (br. s, 1H, OH), 2.32 (s, 3H, $PhCH_3$), 2.28 (s, 0.37H, $PhCH_3$), 2.10 – 1.85 (m, 1.6H, CH_2), 1.85 – 1.55 (m, 2.5H, CH_2), 1.53 – 1.26 (m, 1.6H, CH_2), 0.92 – 0.72 (m, 3.8H, CH_3); ^{13}C NMR (75 MHz, $CDCl_3$) δ 142.4, 139.0, 136.8, 135.9, 131.9, 128.1, 127.9, 127.2, 124.4, 121.3, 121.0, 117.5, 116.6, 74.5, 71.5, 54.7, 54.6, 53.6, 53.1, 32.6, 30.8, 30.1, 28.8, 20.9, 20.6, 15.1, 13.8; HRMS (ESI-TOF): calcd. for $C_{15}H_{22}NO$ $[M + H]^+$ 232.1696; found 232.1700.

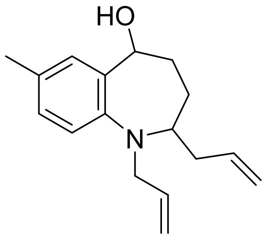

1,2-Diallyl-7-methyl-2,3,4,5-tetrahydro-*1H*-benzo[*b*]azepin-5-ol (2h, anti)

Colourless oil (231 mg, 90%); ^1H NMR (300 MHz, $CDCl_3$) δ 7.09 – 6.91 (m, 2H, phenyl-H), 6.88 – 6.80 (m, 1H, phenyl-H), 5.98 – 5.78 (m, 1H, $NCH_2CH{=}CH_2$), 5.76 – 5.56 (m, 1H, $CH_2CH{=}CH_2$), 5.33 – 5.12 (m, 2H, $NCH_2CH{=}CH_2$), 5.05 – 4.85 (m, 2H, $CH_2CH{=}CH_2$), 4.66 (br. s, 1H, C*H* (HO)), 4.51 (br. s, 1H, OH), 3.85 (ddt, *J* = 14.1, 4.8, 1.8 Hz, 1H, $CH_2CH{=}CH_2$), 3.69 (ddt, *J* = 14.1, 7.5, 0.9 Hz, 1H, $NCH_2CH{=}CH_2$), 3.34 – 3.16 (m, 1H, C*H*(Allyl)), 2.28 (s, 3H, CH_3), 2.18 – 2.01 (m, 2H, CH_2), 2.01 – 1.78 (m, 2H, CH_2), 1.78 – 1.51 (m, 2H, CH_2); ^{13}C NMR (75 MHz,

$CDCl_3$) δ 143.6, 138.0, 136.2, 136.0, 132.4, 128.5, 128.0, 121.2, 117.7, 116.8, 74.8, 58.1, 55.6, 33.0, 27.9, 26.1, 20.6; MS (ESI) *m/z* 258.0 $[M + H]^+$. HRMS (ESI-TOF): calcd. for $C_{17}H_{24}NO$ $[M + H]^+$ 258.1852; found 258.1861.

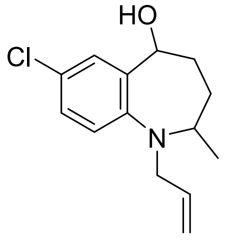

1-Allyl-7-chloro-2-methyl-2,3,4,5-tetrahydro-*1H*-benzo[*b*]azepin-5-ol (2i, syn/anti = 67/33)

Colourless oil (231 mg, 92%); ^1H NMR (300 MHz, $CDCl_3$) δ 7.44 (d, J = 2.1 Hz, 0.7H, phenyl-H), 7.17 – 7.10 (m, 1.6H, phenyl-H), 6.94 – 6.75 (m, 1.3H, phenyl-H), 5.95 – 5.70 (m, 1.1H, $CH_2CH{=}CH_2$), 5.32 – 5.07 (m, 2.9H, $CH_2CH{=}CH_2$), 4.96 (dd, J = 8.7, 4.5 Hz, 1H, C*H* (HO)), 4.68 (br. s, 0.4H, C*H* (HO)), 4.47 (br. s, 0.4 H, OH), 3.87 – 3.71 (m, 1.5H, $CH_2CH{=}CH_2$), 3.70 – 3.55 (m, 1.5H, $CH_2CH{=}CH_2$), 3.44 – 3.24 (m, 1.4H, $CH(CH_3)$), 2.29 (br. s, 0.7H, OH), 2.16 – 1.91 (m, 1.5H, CH_2), 1.92 – 1.74 (m, 1.6H, CH_2), 1.73 – 1.53 (m, 1.3H, CH_2), 1.53 – 1.29 (m, 1.5H, CH_2), 0.83 (d, J = 6.6 Hz, 1.6H, CH_3), 0.76 (d, J = 6.6 Hz, 3H, CH_3); ^{13}C NMR (75 MHz, $CDCl_3$) δ 144.5, 143.5, 141.3, 139.7, 136.3, 135.3, 127.9, 127.3, 127.1, 126.5, 123.9, 122.4, 122.3, 118.0, 117.0, 73.5, 70.8, 55.0, 54.6, 53.8, 52.7, 32.6, 30.5, 29.5, 28.2, 14.8, 14.3; MS (ESI) *m/z* 252.4 $[M + H]^+$. HRMS (ESI-TOF): calcd. for $C_{14}H_{19}ClNO$ $[M + H]^+$ 252.1150; found 252.1159.

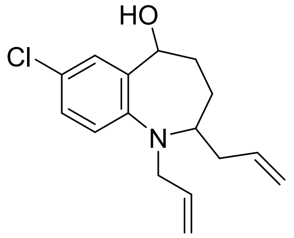

1,2-Diallyl-7-chloro-2,3,4,5-tetrahydro-*1H*-benzo[*b*]azepin-5-ol (2j, anti)

Colourless oil (252 mg, 91%); ^{1}H NMR (300 MHz, $CDCl_3$) δ 7.17 – 7.09 (m, 2H, phenyl-H), 6.92 – 6.82 (m, 1H, phenyl-H), 5.90 – 5.76 (m, 1H, $NCH_2CH{=}CH_2$), 5.75 – 5.55 (m, 1H, $CH_2CH{=}CH_2$), 5.32 – 5.14 (m, 1H, $NCH_2CH{=}CH_2$), 5.08 – 4.87 (m, 1H, $CH_2CH{=}CH_2$), 4.69 (br. s, 1H, C*H* (HO)), 4.20 (br. s, 1H, OH), 3.83 (ddt, *J* = 14.1, 4.8, 1.5 Hz, 1H, $NCH_2CH{=}CH_2$), 3.76 – 3.65 (m, 1H, $NCH_2CH{=}CH_2$), 3.32 – 3.22 (m, 1H, C*H*(Allyl)), 2.22 – 1.98 (m, 2H, CH_2), 1.95 – 1.71 (m, 3H, CH_2 and $CH_2CH{=}CH_2$), 1.67 – 1.52 (m, 1H, CH_2); ^{13}C NMR (75 MHz, $CDCl_3$) δ 144.8, 139.5, 135.7, 135.3, 127.8, 127.4, 127.3, 122.4, 118.2, 117.1, 73.8, 58.2, 55.5, 33.5, 27.4, 25.7; HRMS (ESI-TOF): calcd. for $C_{16}H_{21}ClNO$ $[M + H]^+$ 278.1306; found 278.1309.

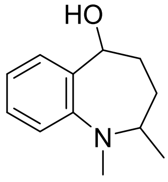

1,2-Dimethyl-2,3,4,5-tetrahydro-*1H*-benzo[*b*]azepin-5-ol (2k, syn/anti = 68/32)

Colourless oil (176 mg, 92%); ^{1}H NMR (300 MHz, $CDCl_3$) δ 7.31 – 7.12 (m, 2.6H, phenyl-H), 7.07 – 6.90 (m, 2.4H, phenyl-H), 4.80 (t, *J* = 4.5 Hz, 0.8 H, C*H* (HO)), 4.77 (d, *J* = 6.3 Hz, 0.4H, C*H* (HO)) 4.12 (br. s, 1H, OH), 3.25 – 3.13 (m, 0.4H, C*H*(CH_3)), 3.12 – 2.97 (m, 0.8H, C*H*(CH_3)), 2.88 – 2.78 (m, 4.6H, NCH_3), 2.12 – 1.64 (m, 4.8H, CH_2), 1.62 – 1.31 (m, 1.5H, CH_2), 1.05 (d, *J* = 6.6 Hz, 3H, CH_3), 0.91

(d, J = 6.6 Hz, 2H, CH_3); ^{13}C NMR (75 MHz, $CDCl_3$) δ 147.1, 146.8, 137.6, 137.4, 127.5, 127.3, 126.3, 124.7, 122.4, 122.2, 119.10, 119.09, 73.1, 72.6, 57.3, 39.9, 39.6, 31.6, 29.8, 29.2, 28.1, 16.1, 14.5; MS (ESI) m/z 192.3 $[M + H]^+$. HRMS (ESI-TOF): calcd. for $C_{12}H_{18}NO$ $[M + H]^+$ 192.1383; found 192.1387.

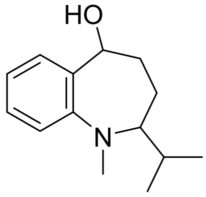

2-Isopropyl-1-methyl-2,3,4,5-tetrahydro-*1H*-benzo[*b*]azepin-5-ol (2l, syn)

Colourless oil (199 mg, 91%); ^1H NMR (300 MHz, $CDCl_3$) δ 7.49 – 7.40 (m, 1H, phenyl-H), 7.18 (td, J = 7.5, 1.2 Hz, 1H, phenyl-H), 7.06 – 6.91 (m, 2H, phenyl-H), 4.80 (dd, J = 9.9, 4.2 Hz, 1H, C*H*(HO)), 3.01 (s, 3H, NCH_3), 2.89 – 2.72 (m, 1H, C*H*(*i*-Pr)), 2.11 – 1.91 (m, 1H, CH_2), 1.85 – 1.53 (m, 4H, CH and CH_2), 0.85 (d, J = 6.6 Hz, 3H, CH_3), 0.71 (d, J = 6.9 Hz, 3H, CH_3); ^{13}C NMR (75 MHz, $CDCl_3$) δ 147.1, 137.9, 126.8, 123.7, 121.2, 120.3, 71.8, 66.8, 41.6, 31.3, 29.3, 26.2, 20.6, 18.8; HRMS (ESI-TOF): calcd. for $C_{14}H_{22}NO$ $[M + H]^+$ 220.1696; found 220.1706.

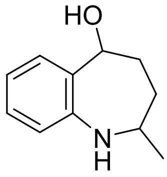

2-Methyl-2,3,4,5-tetrahydro-*1H*-benzo[*b*]azepin-5-ol (2m, syn/anti = 91/9)

White solid (142 mg, 80%); M.p. 103-105 ^oC; ^1H NMR (300 MHz, $CDCl_3$) δ 7.45 – 7.38 (m, 0.1H, phenyl-H), 7.16 (dd, J = 7.5, 1.5 Hz, 0.81H, phenyl-H), 7.10 (td, J =

7.5, 1.5 Hz, 0.85H, phenyl-H), 7.00 – 6.87 (m, 0.85H, phenyl-H), 6.80 – 6.72 (m, 0.9H, phenyl-H), 4.80 (d, *J* = 8.7, 1.8 Hz, 0.1H, C*H*(OH)), 4.76 (d, *J* = 6.6 Hz, 0.9H, C*H*(OH)), 3.52 (br. s, 2H, OH and NH), 3.12 – 2.86 (m, 1.2H, C*H*(CH_3)), 2.29 – 2.11 (m, 1H, CH_2), 2.04 – 1.85 (m, 1.2H, CH_2), 1.81 – 1.63 (m, 2.1H, CH_2), 1.28 (d, *J* = 6.6 Hz, 3H, CH_3), 1.21 (d, *J* = 6.6 Hz, 0.3H, CH_3); ^{13}C NMR (75 MHz, $CDCl_3$) δ 146.9, 135.6, 128.9, 128.1, 127.2, 126.4, 122.0, 121.1, 120.8, 120.1, 74.5, 72.5, 54.6, 52.6, 34.5, 33.5, 32.4, 32.0, 23.9, 23.0; HRMS (ESI-TOF): calcd. for $C_{11}H_{16}NO$ [M + H]$^+$ 178.1226; found 178.1232.

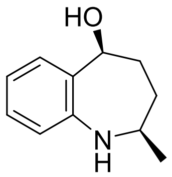

2-Methyl-2,3,4,5-tetrahydro-*1H*-benzo[*b*]azepin-5-ol syn-2m

^1H NMR (300 MHz, $CDCl_3$) δ 7.16 (dd, *J* = 7.5, 1.5 Hz, 1H, phenyl-H), 7.10 (td, *J* = 7.5, 1.5 Hz, 1H, phenyl-H), 6.92 (td, *J* = 7.5, 1.2 Hz, 1H, phenyl-H), 6.75 (dd, *J* = 7.5, 1.2 Hz, 1H, phenyl-H), 4.76 (d, *J* = 6.6 Hz, 1H, C*H*(OH)), 3.56 (br. s, 2H, OH and NH), 3.08 – 2.81 (m, 1H, C*H*(CH_3)), 2.34 – 2.07 (m, 1H, CH_2), 2.06 – 1.85 (m, 1H, CH_2), 1.82 – 1.59 (m, 2H, CH_2), 1.28 (d, *J* = 6.6 Hz, 3H, CH_3); MS (ESI) *m/z* 177.9 [M + H]$^+$. ^{13}C NMR (75 MHz, $CDCl_3$) δ 146.9, 135.6, 128.9, 128.1, 122.1, 120.9, 74.6, 54.7, 32.4, 32.0, 24.0. HRMS (ESI-TOF): calcd. for $C_{11}H_{16}NO$ [M + H]$^+$ 178.1226; found 178.1230. CCDC 1033762 contains the supplementary crystallographic data for this compound.

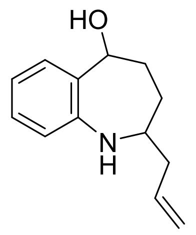

2-Allyl-2,3,4,5-tetrahydro-*1H*-benzo[*b*]azepin-5-ol (2n, syn/anti = 75/25)

Colourless oil (164 mg, 81%); ^{1}H NMR (300 MHz, $CDCl_3$) δ 7.45 – 7.38 (m, 0.3H, phenyl-H), 7.16 (dd, J = 7.5, 1.5 Hz, 1H, phenyl-H), 7.13 – 7.05 (m, 1.2H, phenyl-H), 6.99 – 6.87 (m, 1.2H, phenyl-H), 6.75 – 6.68 (m, 1.3H, phenyl-H), 5.94 – 5.70 (m, 1.1H, $CH_2CH{=}CH_2$), 5.29 – 5.11 (m, 2.7H, $CH_2CH{=}CH_2$), 4.80 (dd, J = 9.0, 1.8 Hz, 0.3H, C*H*(OH)), 4.77 (d, J = 6.6 Hz, 1H, C*H*(OH)), 3.63 (br.s, 1.4H, NH), 2.98 – 2.75 (m, 1.4H, C*H*(Allyl)), 2.42 – 2.31 (m, 1.3H, CH_2), 2.31 – 2.14 (m, 2.6H, CH_2), 2.14 – 1.87 (m, 2H, CH_2), 1.84 – 1.56 (m, 2.6H, CH_2); ^{13}C NMR (75 MHz, CDC_3) δ 146.8, 146.2, 135.8, 135.43, 135.37, 135.2, 128.9, 128.1, 127.3, 126.4, 122.1, 121.2, 120.8, 120.1, 118.5, 118.2, 74.5, 72.4, 57.6, 55.8, 41.8, 41.1, 33.6, 33.0, 32.4, 30.6; MS (ESI) *m*/*z* 203.9 $[M + H]^+$. HRMS (ESI-TOF): calcd. for $C_{13}H_{18}NO$ $[M + H]^+$ 204.1383; found 204.1391.

General Procedure for the Synthesis of 3

To a solution of compound **2** (0.5 mmol) in dry CH_2Cl_2 (10 mL) at -10 °C was added Dess-Martin (0.6 mmol). The resulting solution was stirred at -10 °C until compound **2** had been consumed, as monitored by TLC. The mixture was extracted with CH_2Cl_2 (3 × 15 mL). The combined organic extracts were washed with brine, dried over anhydrous Na_2SO_4 and concentrated *in vacuo*. Purification by flash column chromatography (petroleum ether/ethyl acetate = 10:1, v/v) afforded the desired

product **3**.

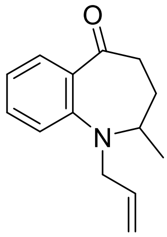

1-Allyl-2-methyl-3,4-dihydro-*1H*-benzo[*b*]azepin-5(*2H*)-one (3a)

Colourless oil (91 mg, 85%); ^{1}H NMR (300 MHz, $CDCl_3$) δ 7.79 – 7.66 (m, 1H, phenyl-H), 7.47 – 7.30 (m, 1H, phenyl-H), 6.95 (dd, J = 11.1, 4.2 Hz, 2H, phenyl-H), 5.98 – 5.70 (m, 1H, $CH_2CH{=}CH_2$), 5.36 – 5.09 (m, 2H, $CH_2CH{=}CH_2$), 4.03 – 3.83 (m, 2H, $CH_2CH{=}CH_2$), 3.65 – 3.39 (m, 1H, $CH(CH_3)$), 2.85 – 2.60 (m, 2H, CH_2), 2.34 – 2.08 (m, 1H, CH_2), 1.85 – 1.64 (m, 1H, CH_2), 1.04 (d, J = 6.3 Hz, 3H, CH_3); ^{13}C NMR (75 MHz, $CDCl_3$) δ 204.2, 150.0, 136.0, 132.6, 131.9, 129.3, 121.0, 120.8, 117.1, 56.4, 52.5, 40.3, 34.4, 16.4; HRMS (ESI-TOF): calcd. for $C_{14}H_{18}NO$ $[M + H]^+$ 216.1383; found 216.1376.

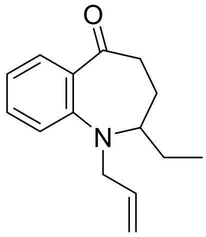

1-Allyl-2-ethyl-3,4-dihydro-*1H*-benzo[*b*]azepin-5(*2H*)-one (3b)

Colourless oil (95 mg, 83%); ^{1}H NMR (300 MHz, $CDCl_3$) δ 7.74 (dd, J = 7.8, 1.8 Hz, 1H, phenyl-H), 7.42 – 7.30 (m, 1H, phenyl-H), 6.98 (d, J = 8.1 Hz, 1H, phenyl-H), 6.91 (t, J = 7.5 Hz, 1H, phenyl-H), 6.03–5.77 (m, 1H, $CH_2CH{=}CH_2$), 5.27 (dq, J = 17.1, 1.5 Hz, 1H, $CH_2CH{=}CH_2$), 5.18 (dq, J = 10.2, 1.5 Hz, 1H, $CH_2CH{=}CH_2$), 4.14 – 3.93 (m, 2H, $CH_2CH{=}CH_2$), 3.38 – 3.22 m, 1H), 2.82 – 2.66 (m, 2H), 2.49 – 2.27 (m,

1H), 1.94 – 1.61 (m, 2H), 1.39 – 1.19 (m, 1H), 0.79 (t, J = 7.5 Hz, 3H); ^{13}C NMR (75 MHz, $CDCl_3$) δ 203.8, 151.1, 136.1, 132.6, 130.5, 129.2, 120.1, 119.9, 117.3, 62.9, 53.0, 40.4, 33.4, 25.1, 11.1; HRMS (ESI-TOF): calcd. for $C_{15}H_{20}NO$ $[M + H]^+$ 230.1539; found 230.1552.

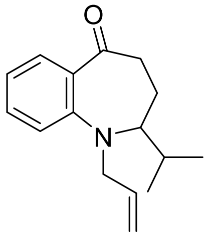

1-Allyl-2-isopropyl-3,4-dihydro-*1H*-benzo[*b*]azepin-5(*2H*)-one (3c)

Colourless oil (91 mg, 86%); ^{1}H NMR (300 MHz, $CDCl_3$) δ 7.80 – 7.69 (m, 1H, phenyl-H), 7.38 – 7.29 (m, 1H, phenyl-H), 7.06 – 6.94 (m, 1H, phenyl-H), 6.91 – 6.80 (m, 1H, phenyl-H), 6.05 – 5.75 (m, 1H, $CH_2CH{=}CH_2$), 5.35 – 5.11 (m, 2H, $CH_2CH{=}CH_2$), 4.22 – 4.01 (m, 2H, $CH_2CH{=}CH_2$), 3.13 – 2.97 (m, 1H, *CH*(*i*-Pr)), 2.84 – 2.64 (m, 2H, CH_2), 2.45 – 2.25 (m, 1H, CH_2), 2.15 – 1.98 (m, 1H, CH_2), 1.98 – 1.85 (m, 1H, CH), 0.82 (d, J = 6.6 Hz, 3H, CH_3), 0.78 (d, J = 6.6 Hz, 3H, CH_3); ^{13}C NMR (75 MHz, $CDCl_3$) δ 203.2, 152.3, 136.0, 132.6, 129.5, 129.2, 119.7, 119.4, 117.6, 76.6, 68.7, 53.6, 40.6, 31.9, 30.4, 20.8, 19.8; HRMS (ESI-TOF): calcd. for $C_{16}H_{22}NO$ $[M + H]^+$ 244.1696; found 244.1687.

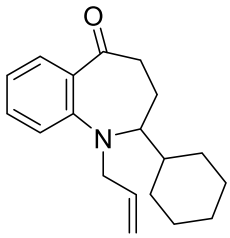

1-Allyl-2-cyclohexyl-3,4-dihydro-*1H*-benzo[*b*]azepin-5(*2H*)-one (3d)

Colourless oil (113 mg, 80%); ^{1}H NMR (300 MHz, $CDCl_3$) δ 7.74 (dd, J = 7.8, 1.8 Hz,

1H, phenyl-H), 7.38 – 7.28 (m, 1H, phenyl-H), 6.99 (d, *J* = 8.4 Hz, 1H, phenyl-H), 6.85 (t, *J* = 7.5 Hz, 1H, phenyl-H), 6.05 – 5.75 (m, 1H, $CH_2C\textit{H}=CH_2$), 5.40 – 5.10 (m, 2H, $CH_2CH=C\textit{H}_2$), 4.21 – 3.95 (m, 2H, $\textit{CH}_2CH=CH_2$), 3.18 – 3.01 (m, 1H, C*H*(Cy)), 2.86 – 2.63 (m, 2H, CH_2), 2.45 – 2.23 (m, 1H, CH_2), 2.19 – 1.97 (m, 1H, CH_2), 1.74 – 1.47 (m, 6H, CH_2), 1.30 – 0.96 (m, 3H, CH_2 and CH), 0.95 – 0.70 (m, 2H, CH_2); ^{13}C NMR (75 MHz, $CDCl_3$) δ 203.2, 152.7, 136.1, 132.5, 129.4, 129.2, 119.5, 119.3, 117.5, 67.8, 53.2, 40.6, 40.1, 32.1, 31.1, 30.2, 26.2, 26.0, 25.9; HRMS (ESI-TOF): calcd. for $C_{19}H_{26}NO$ $[M + H]^+$ 284.2009; found 284.2002.

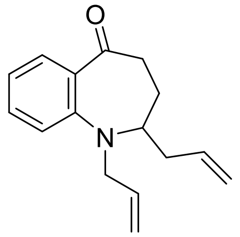

1,2-Diallyl-3,4-dihydro-*1H*-benzo[*b*]azepin-5(*2H*)-one (3e)

Light brown oil (90 mg, 75%); ^1H NMR (300 MHz, $CDCl_3$) δ 7.75 (dd, *J* = 7.8, 1.8 Hz, 1H, phenyl-H), 7.45 – 7.30 (m, 1H, phenyl-H), 7.07 – 6.88 (m, 2H, phenyl-H), 5.98 – 5.76 (m, 1H, $NCH_2C\textit{H}=CH_2$), 5.75 – 5.54 (m, 1H, $CH_2C\textit{H}=CH_2$), 5.40 – 5.12 (m, 2H, $NCH_2CH=C\textit{H}_2$), 5.04 – 4.88 (m, 2H, $CH_2CH=C\textit{H}_2$), 4.14 – 3.90 (m, 2H, $N\textit{CH}_2CH=CH_2$), 3.56 – 3.34 (m, 1H, C*H*(Allyl)), 2.80 – 2.66 (m, 2H, CH_2), 2.55 – 2.40 (m, 1H, $\textit{CH}_2CH=CH_2$), 2.39 – 2.21 (m, 1H, CH_2), 2.05 – 1.88 (m, 1H, $\textit{CH}_2CH=CH_2$), 1.85 – 1.66 (m, 1H, CH_2); ^{13}C NMR (75 MHz, $CDCl_3$) δ 203.8, 150.2, 135.9, 135.0, 132.8, 131.5, 129.3, 121.0, 120.9, 117.5, 117.1, 60.7, 53.4, 40.3, 36.3, 32.4; HRMS (ESI-TOF): calcd. for $C_{16}H_{20}NO$ $[M + H]^+$ 242.1539; found 242.1537.

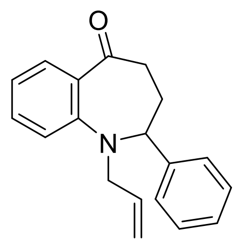

1-Allyl-2-phenyl-3,4-dihydro-*1H*-benzo[*b*]azepin-5(*2H*)-one (3f)

Colourless oil (122 mg, 88%); ^{1}H NMR (300 MHz, $CDCl_3$) δ 7.79 (dd, J = 7.8, 1.5 Hz, 1H, phenyl-H), 7.40 (ddd, J = 8.1, 7.2, 1.8 Hz, 1H, phenyl-H), 7.31 – 7.22 (m, 3H, phenyl-H), 7.07 (ddd, J = 7.8, 7.2, 1.2 Hz, 1H, phenyl-H), 7.03 – 6.94 (m, 2H, phenyl-H), 6.75 (dd, J = 8.1, 0.9 Hz, 1H, phenyl-H), 5.92 – 5.63 (m, 1H, $CH_2CH{=}CH_2$), 5.23 – 5.08 (m, 2H, $CH_2CH{=}CH_2$), 4.36 (dd, J = 11.7, 6.0 Hz, 1H, C*H*(Ph)), 3.80 – 3.35(m, 2H, $CH_2CH{=}CH_2$), 2.94 – 2.69 (m, 2H, CH_2), 2.44 – 2.26 (m, 1H, CH_2), 2.25 – 2.02 (m, 1H, CH_2); ^{13}C NMR (75 MHz, $CDCl_3$) δ 204.8, 149.0, 140.4, 135.5, 133.12, 133.08, 129.6, 128.2, 127.8, 127.4, 122.7, 122.1, 117.8, 65.3, 53.7, 40.0, 31.1; HRMS (ESI-TOF): calcd. for $C_{19}H_{20}NO$ $[M + H]^+$ 278.1539; found 278.1545.

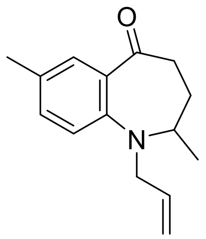

1-Allyl-2,7-dimethyl-3,4-dihydro-*1H*-benzo[*b*]azepin-5(*2H*)-one (3g)

Colourless oil (101 mg, 88%); ^{1}H NMR (300 MHz, $CDCl_3$) δ 7.52 (d, J = 2.1 Hz, 1H, phenyl-H), 7.20 (ddd, J = 8.1, 2.4, 0.6 Hz, 1H, phenyl-H), 6.87 (d, J = 8.4 Hz, 1H, phenyl-H), 5.95 – 5.69 (m, 1H, $CH_2CH{=}CH_2$), 5.35 – 5.05 (m, 2H, $CH_2CH{=}CH_2$), 4.04 – 3.76 (m, 2H, $CH_2CH{=}CH_2$), 3.56 – 3.35 (m, 1H, C*H*(CH_3)), 2.78 – 2.62 (m, 2H, CH_2), 2.29 (s, 3H, $PhCH_3$), 2.23 – 2.06 (m, 1H, CH_2), 1.71 – 1.52 (m, 1H, CH_2), 1.00

(d, J = 6.3 Hz, 3H, CH_3); ^{13}C NMR (75 MHz, $CDCl_3$) δ 204.9, 147.0, 136.1, 133.6, 132.4, 130.9, 129.4, 121.5, 117.0, 56.0, 52.9, 40.3, 33.5, 20.3, 15.7; HRMS (ESI-TOF): calcd. for $C_{15}H_{20}NO$ $[M + H]^+$ 230.1539; found 230.1544.

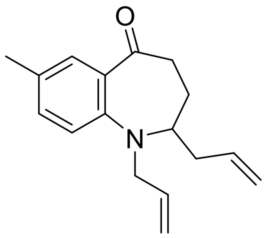

1,2-Diallyl-7-methyl-3,4-dihydro-*1H*-benzo[*b*]azepin-5(*2H*)-one (3h)

Colourless oil (97 mg, 76%); ^{1}H NMR (300 MHz, $CDCl_3$) δ 7.55 (d, J = 2.4 Hz, 1H, phenyl-H), 7.19 (ddd, J = 8.4, 2.4, 0.6 Hz, 1H, phenyl-H), 6.88 (d, J = 8.4 Hz, 1H, phenyl-H), 5.90 – 5.75 (m, 1H, $NCH_2CH{=}CH_2$), 5.74 – 5.57 (m, 1H, $CH_2CH{=}CH_2$), 5.34 – 5.10 (m, 2H, $NCH_2CH{=}CH_2$), 5.01 – 4.90 (m, 2H, $CH_2CH{=}CH_2$), 4.15 – 3.82 (m, 2H, $NCH_2CH{=}CH_2$), 3.48 – 3.31 (m, 1H, *CH*(Allyl)), 2.82 – 2.63 (m, 2H, CH_2), 2.55 – 2.38 (m, 1H, $CH_2CH{=}CH_2$), 2.34 – 2.16 (m, 1H, CH_2), 2.29 (s, 3H, CH_3), 1.97 – 1.82 (m, 1H, $CH_2CH{=}CH_2$), 1.74 – 1.58 (m, 1H, CH_2); ^{13}C NMR (75 MHz, $CDCl_3$) δ 204.3, 147.3, 136.0, 135.1, 133.7, 132.0, 130.9, 129.4, 121.5, 117.3, 117.0, 60.4, 53.7, 40.3, 35.9, 31.6, 20.3; MS (ESI) m/z 256.1 $[M + H]^+$. HRMS (ESI-TOF): calcd. for $C_{17}H_{22}NO$ $[M + H]^+$ 256.1696; found 256.1705.

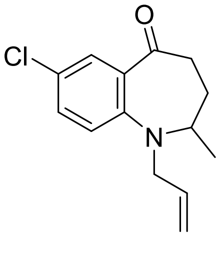

1-Allyl-7-chloro-2-methyl-3,4-dihydro-*1H*-benzo[*b*]azepin-5(*2H*)-one (3i)

White solid (148 mg, 84%); M.P. 56-58 °C; ^{1}H NMR (300 MHz, $CDCl_3$) δ 7.68 (d, J

= 2.7 Hz, 1H, phenyl-H), 7.30 (dd, J = 8.7, 2.7 Hz, 1H, phenyl-H), 6.90 (d, J = 8.7 Hz, 1H, phenyl-H), 5.90 – 5.71 (m, 1H, $CH_2CH{=}CH_2$), 5.32 – 5.12 (m, 2H, $CH_2CH{=}CH_2$), 4.03 – 3.81 (m, 2H, $CH_2CH{=}CH_2$), 3.62 – 3.37 (m, 1H, $CH(CH_3)$), 2.88 – 2.54 (m, 2H, CH_2), 2.35 – 2.12 (m, 1H, CH_2), 1.82 – 1.63 (m, 1H, CH_2), 1.04 (d, J = 6.3 Hz, 3H, CH_3); ^{13}C NMR (75 MHz, $CDCl_3$) δ 202.8, 148.4, 135.4, 132.6, 132.4, 128.8, 126.3, 122.2, 117.5, 56.6, 52.4, 40.1, 34.2, 16.4; HRMS (ESI-TOF): calcd. for $C_{14}H_{17}ClNO$ $[M + H]^+$ 250.0993; found 250.0999.

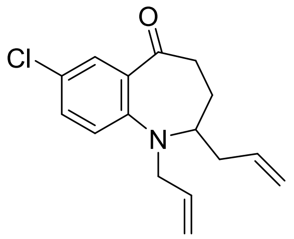

1,2-Diallyl-7-chloro-3,4-dihydro-*1H*-benzo[*b*]azepin-5(*2H*)-one (3j)

White solid (107 mg, 78%); M.P. 49-51 °C; ^1H NMR (300 MHz, $CDCl_3$) δ 7.71 (d, J = 2.7 Hz, 1H, phenyl-H), 7.30 (dd, J = 8.7, 2.7 Hz, 1H, phenyl-H), 6.91 (d, J = 8.7 Hz, 1H, phenyl-H), 5.94 – 5.74 (m, 1H, $NCH_2CH{=}CH_2$), 5.73 – 5.53 (m, 1H, $CH_2CH{=}CH_2$), 5.36 – 5.12 (m, 2H, $NCH_2CH{=}CH_2$), 5.04 – 4.89 (m, 2H, $CH_2CH{=}CH_2$), 4.12 – 4.86 (m, 2H, $NCH_2CH{=}CH_2$), 3.56–3.35 (m, 1H, CH(Allyl)), 2.83 – 2.63 (m, 2H, CH_2), 2.54 – 2.38 (m, 1H, $CH_2CH{=}CH_2$), 2.38 – 2.21 (m, 1H, CH_2), 2.06 – 1.88 (m, 1H, $CH_2CH{=}CH_2$), 1.83 – 1.65 (m, 1H, CH_2); ^{13}C NMR (75 MHz, $CDCl_3$) δ 202.3, 148.6, 135.3, 134.6, 132.5, 132.2, 128.8, 126.3, 122.3, 117.9, 117.4, 60.9, 53.5, 40.1, 36.3, 32.3; HRMS (ESI-TOF): calcd. for $C_{16}H_{19}ClNO$ $[M + H]^+$ 276.1150; found 276.1152.

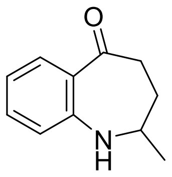

2-Methyl-3,4-dihydro-*1H*-benzo[*b*]azepin-5(*2H*)-one (3k)

Pale yellow oil (33 mg, 38%); ^{1}H NMR (300 MHz, $CDCl_3$) δ 7.71 (dd, J = 7.8, 1.5 Hz, 1H, phenyl-H), 7.27 – 7.19 (m, 1H, phenyl-H), 6.81 (ddd, J = 8.1, 7.2, 0.9 Hz, 1H, phenyl-H), 6.73 (dd, J = 8.1, 0.6 Hz, 1H, phenyl-H), 4.16 (br. s, 1H, NH), 3.35 – 3.12 (m, 1H, C*H*(CH_3)), 3.11 – 2.92 (m, 1H, CH_2), 2.60 (ddd, J = 11.4, 6.6, 2.1 Hz, 1H, CH_2), 2.27 – 2.09 (m, 1H, CH_2), 2.04 – 1.86 (m, 1H, CH_2), 1.33 (d, J = 6.3 Hz, 3H, CH_3); ^{13}C NMR (75 MHz, $CDCl_3$) δ 202.8, 152.3, 132.3, 129.1, 125.1, 118.6, 117.7, 64.0, 54.8, 40.9, 39.0, 21.9; HRMS (ESI-TOF): calcd. for $C_{11}H_{14}NO$ $[M + H]^+$ 176.1070; found 176.1077.

Procedure for the synthesis of 4

To a solution of **3e** (121 mg, 0.5 mmol) in CH_2Cl_2 (5 mL) was added Grubbs II catalyst (42.5 mg, 0.05 mmol) under an Ar atmosphere. The mixture was stirred at room temperature for 8 h. After compound **3e** had been consumed, the reaction mixture was cooled to room temperature. Water was added and the mixture was extracted with ethyl acetate (3 × 15 mL). The combined organic extracts were washed with brine, dried over anhydrous Na_2SO_4 and concentrated *in vacuo*. Purification by flash column chromatography (petroleum ether/ethyl acetate = 10:1, v/v) afforded the desired product **4**. ^{1}H NMR (300 MHz, $CDCl_3$) δ 7.69 (dd, J = 7.5, 1.8 Hz, 1H, phenyl-H), 7.45 – 7.33 (m, 1H, phenyl-H), 7.05 (d, J = 8.4 Hz, 1H, phenyl-H), 7.02 –

6.93 (m, 1H, phenyl-H), 6.03 – 5.82 (m, 2H, CH=CH), 4.11 – 3.99 (m, 1H, CH_2), 3.84 – 3.72 (m, 1H, CH_2), 3.60 – 3.44 (m, 1H, CH), 2.84 – 2.72 (m, 1H, CH_2), 2.67 – 2.57 (m, 1H, CH_2), 2.30 – 2.08 (m, 2H, CH_2), 2.06 – 1.91 (m, 1H, CH_2), 1.86 – 1.73 (m, 1H, CH_2). ^{13}C NMR (75 MHz, $CDCl_3$) δ 204.8, 150.3, 133.1, 132.0, 129.4, 125.8, 125.5, 121.4 120.3, 57.0, 49.1, 39.4, 31.3, 29.6. HRMS (ESI-TOF): calcd. for $C_{14}H_{16}NO$ $[M + H]^+$ 214.1226; found 214.1221.

NMR Spectra

NMR spectrum of Compound **S2a**

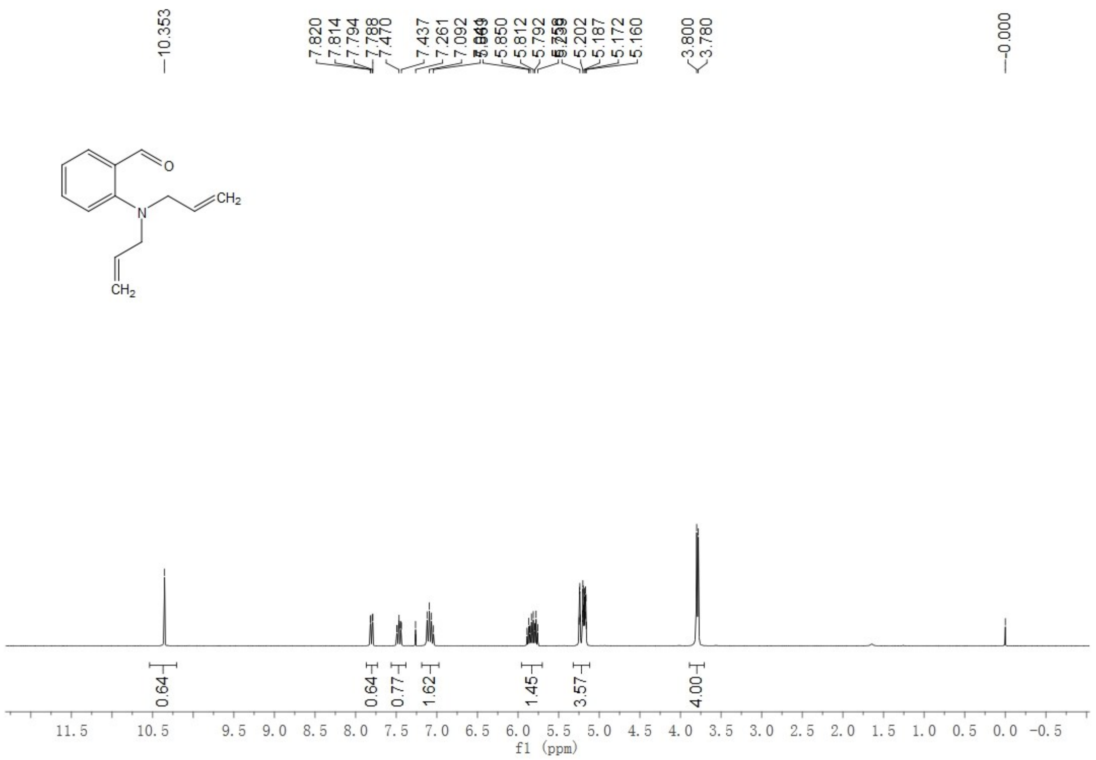

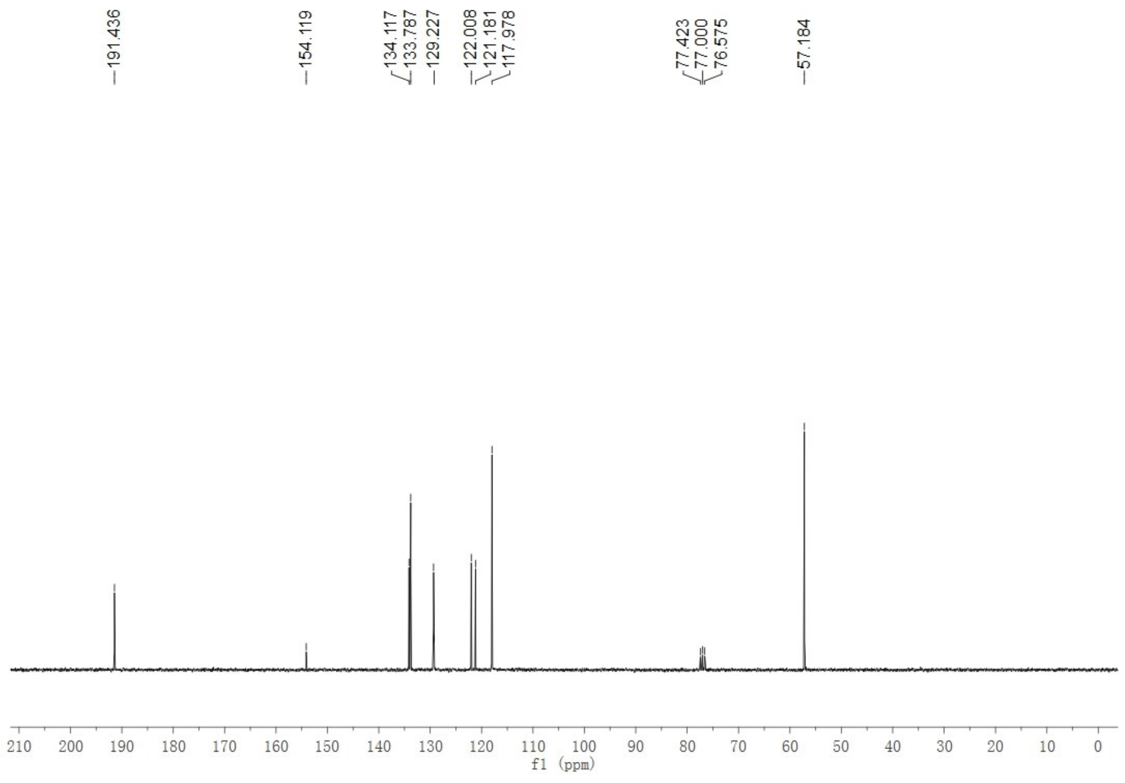

NMR spectrum of Compound **S2b**

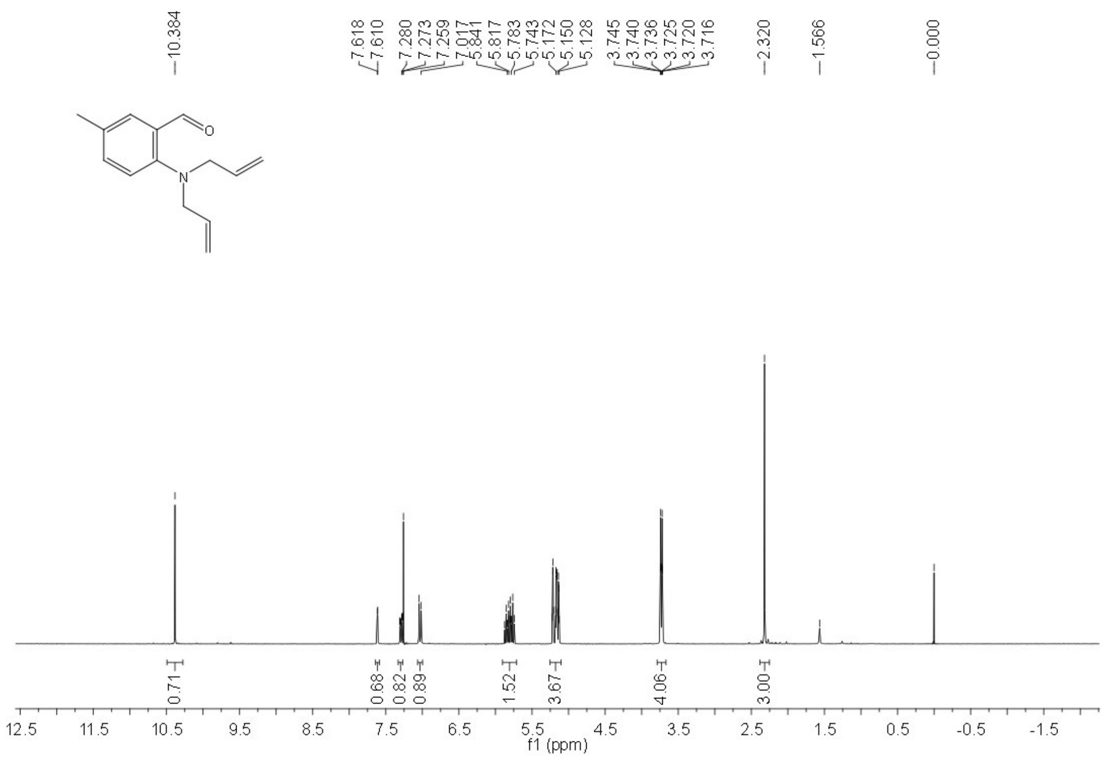

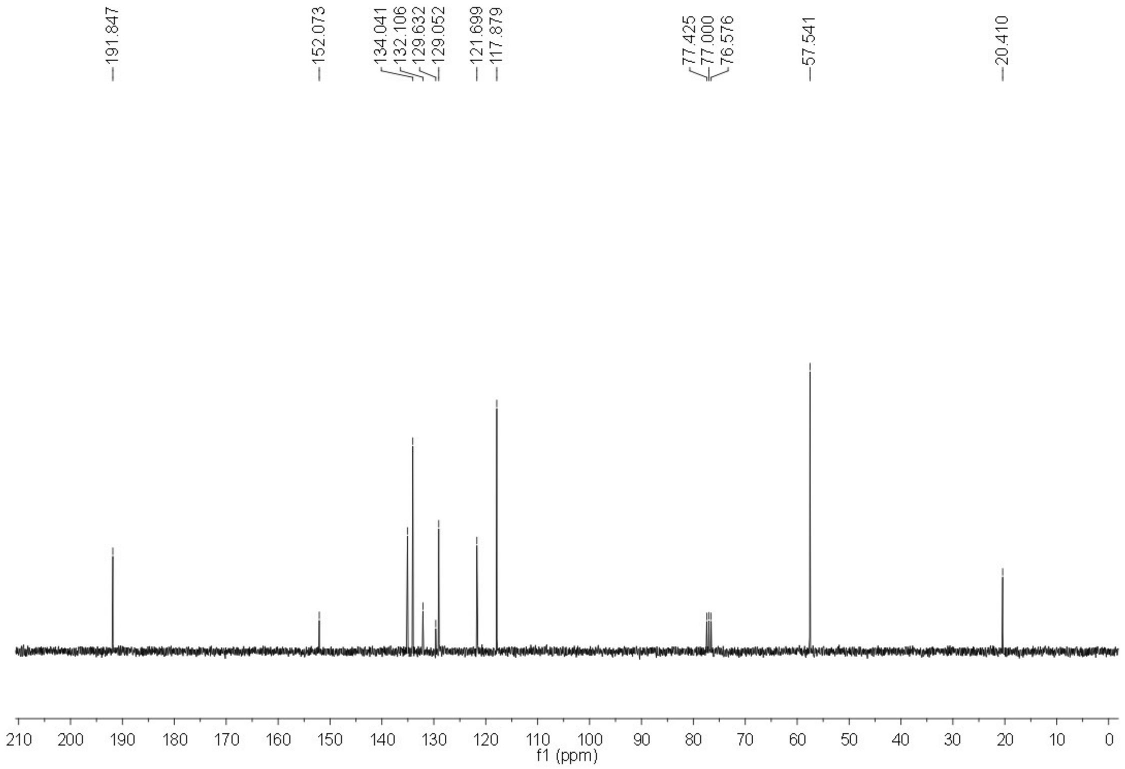

NMR spectrum of Compound **S2c**

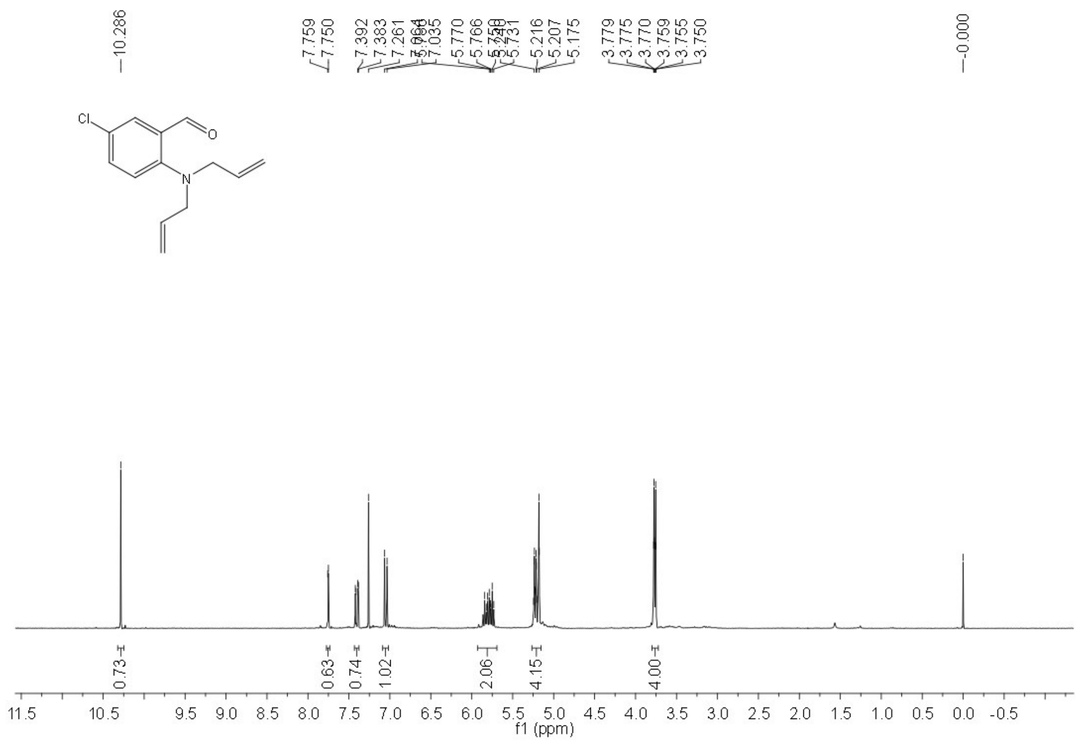

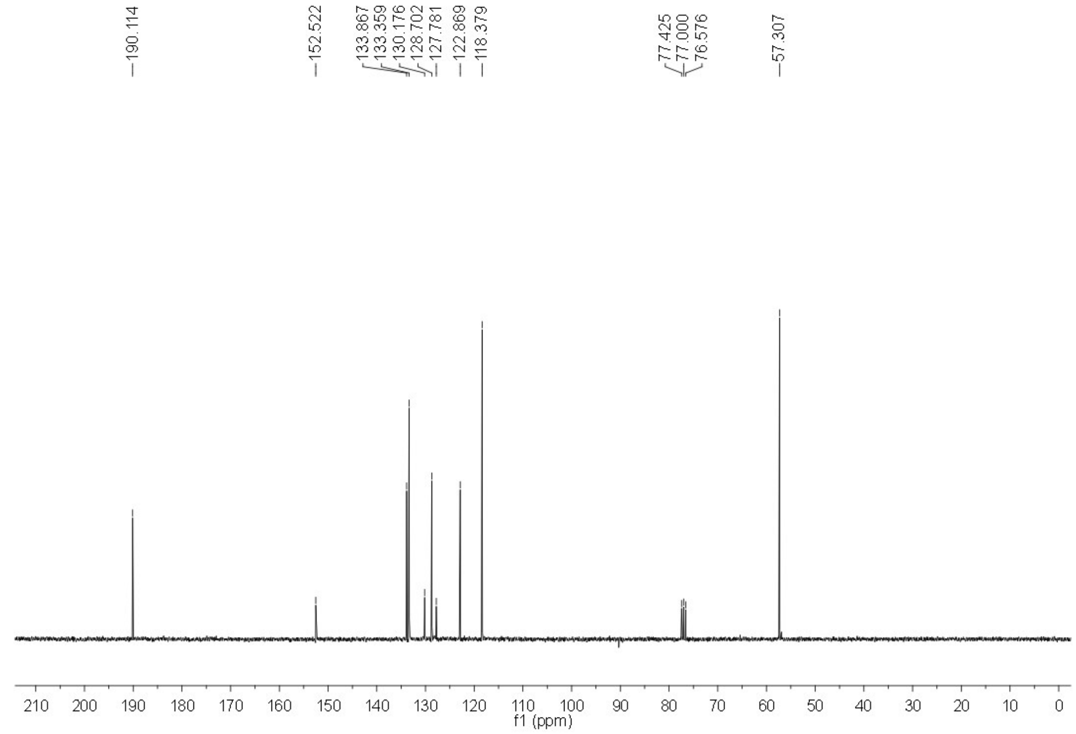

NMR spectrum of Compound **S2d**

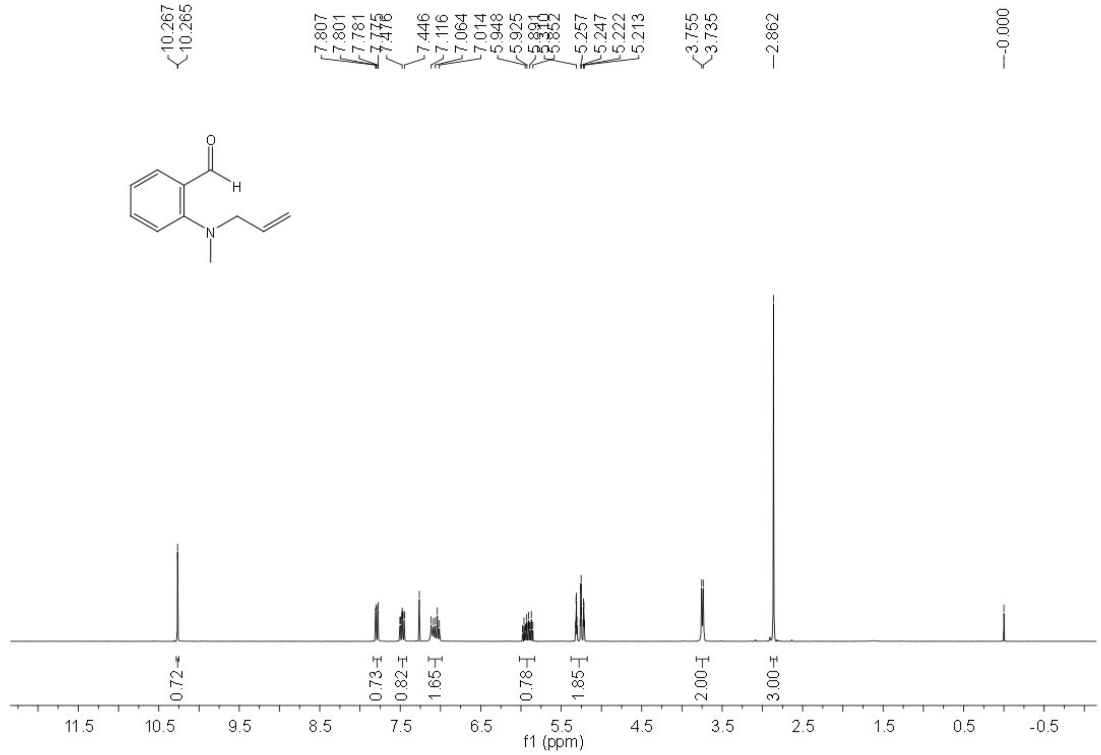

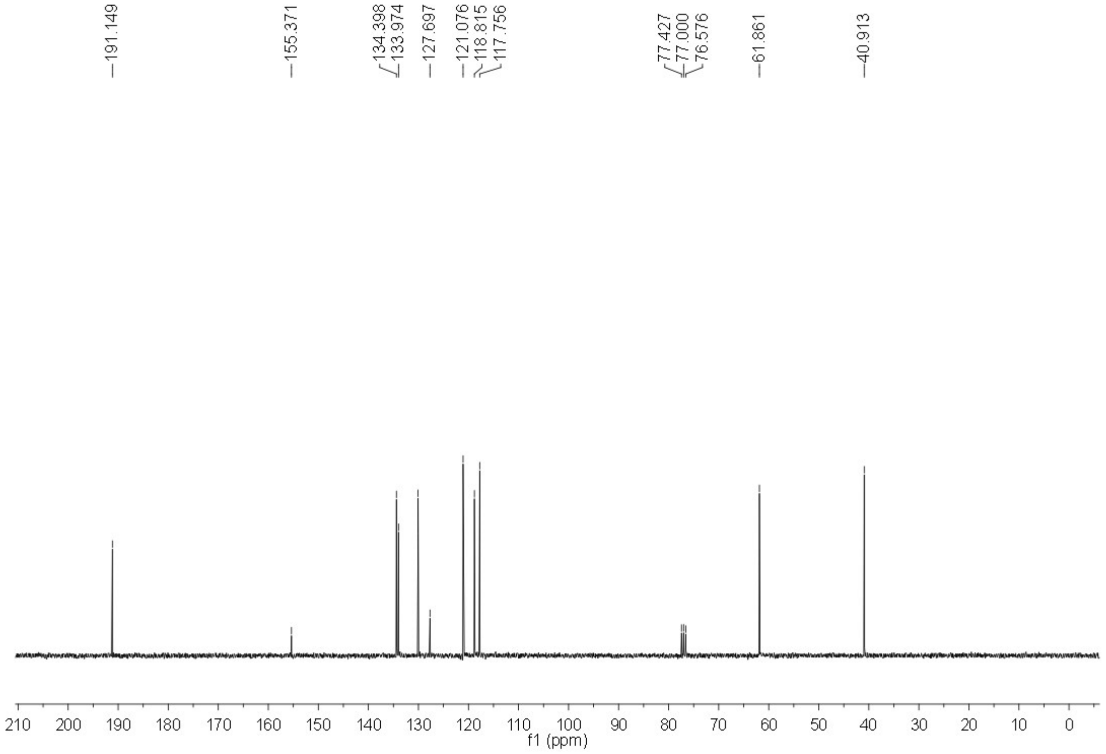

NMR spectrum of Compound **1a**

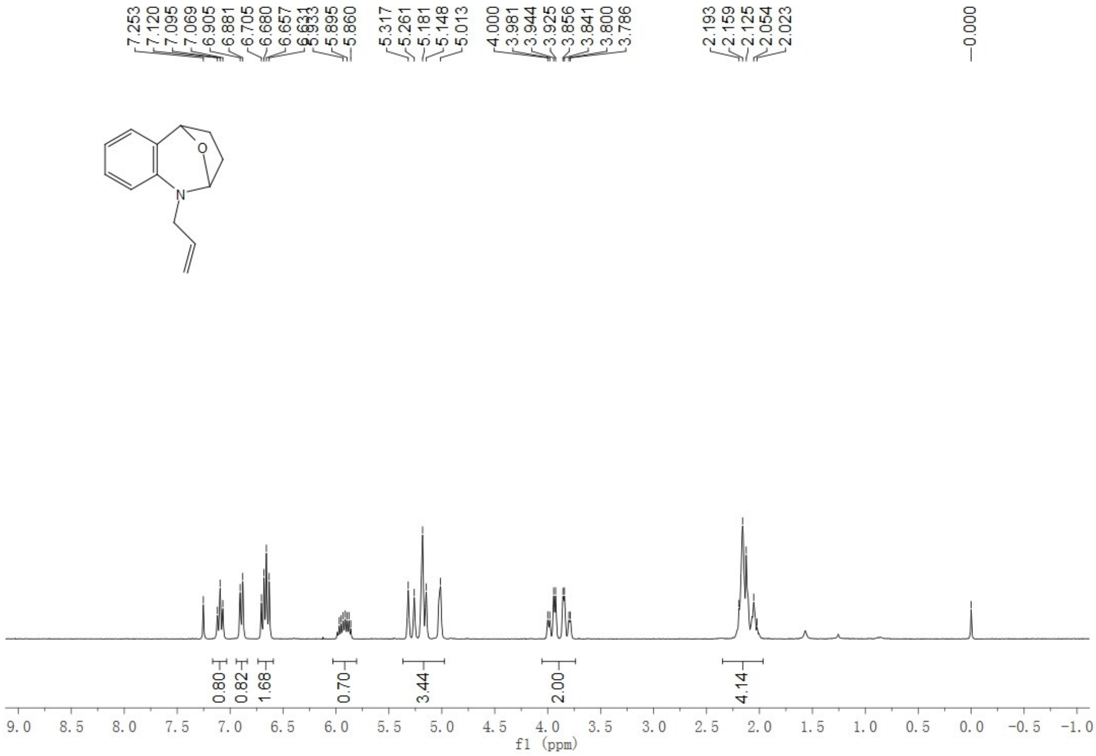

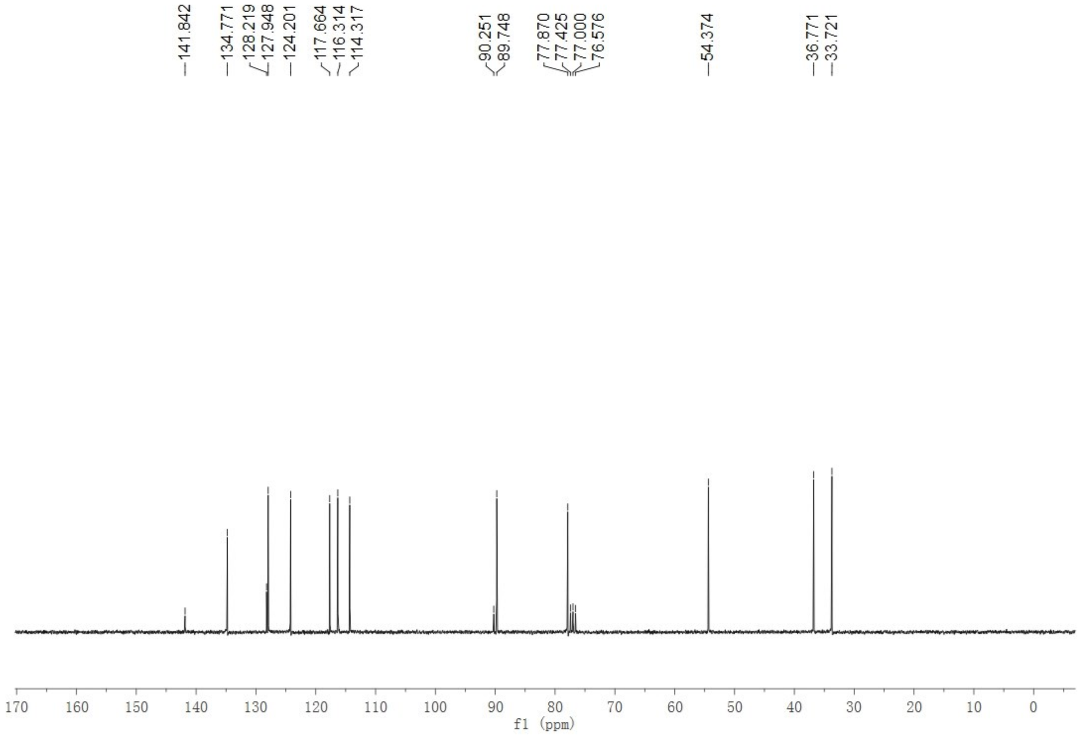

NMR spectrum of Compound **1b**

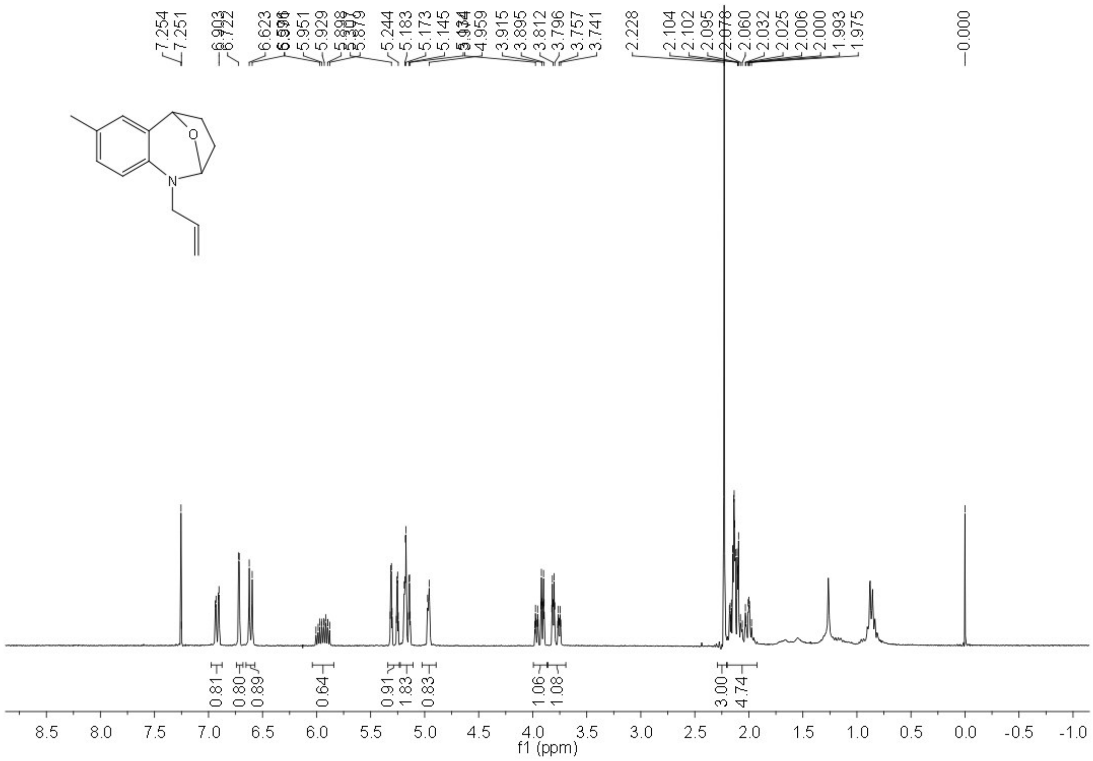

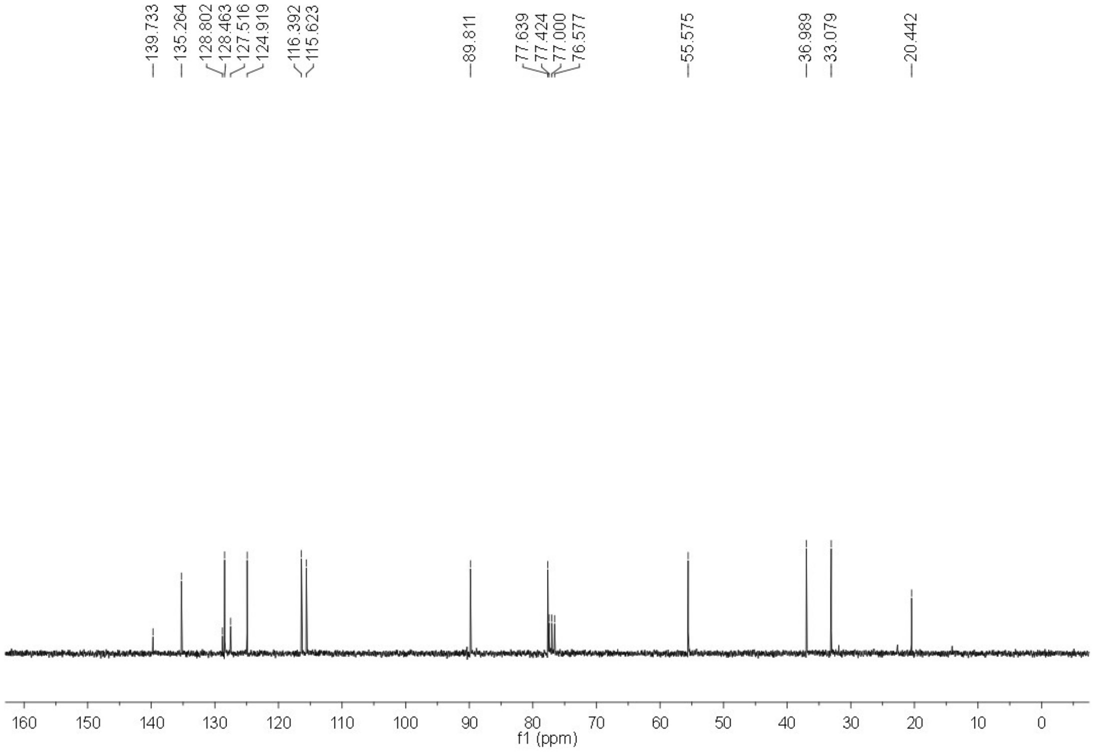

NMR spectrum of Compound **1c**

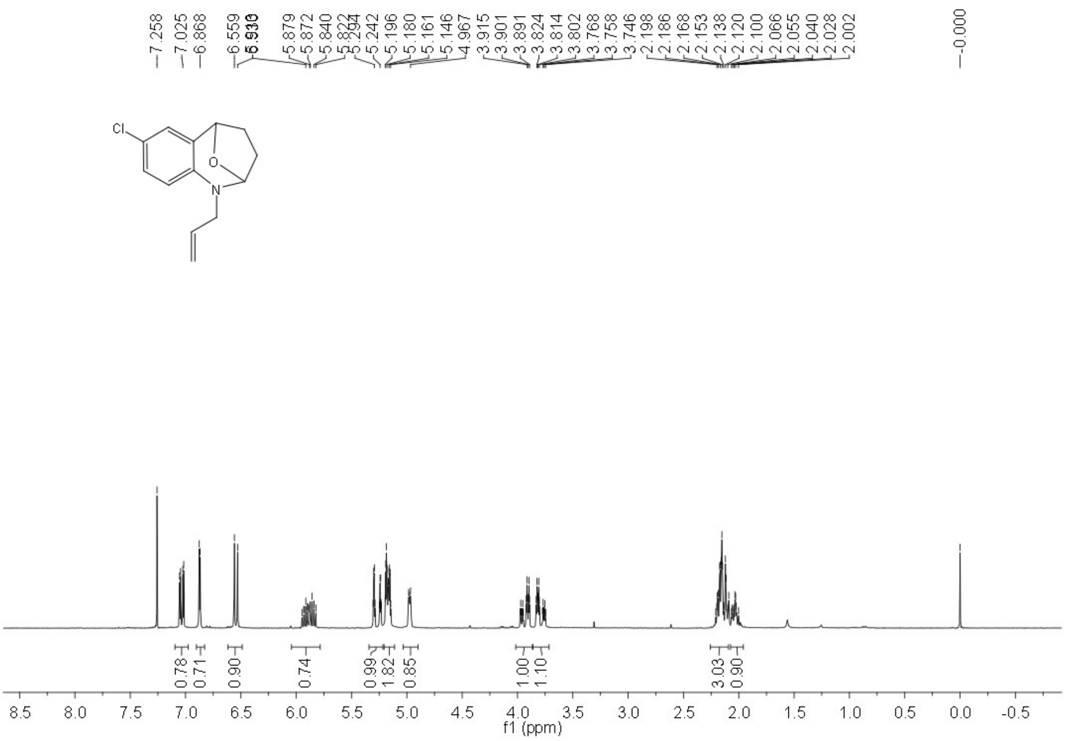

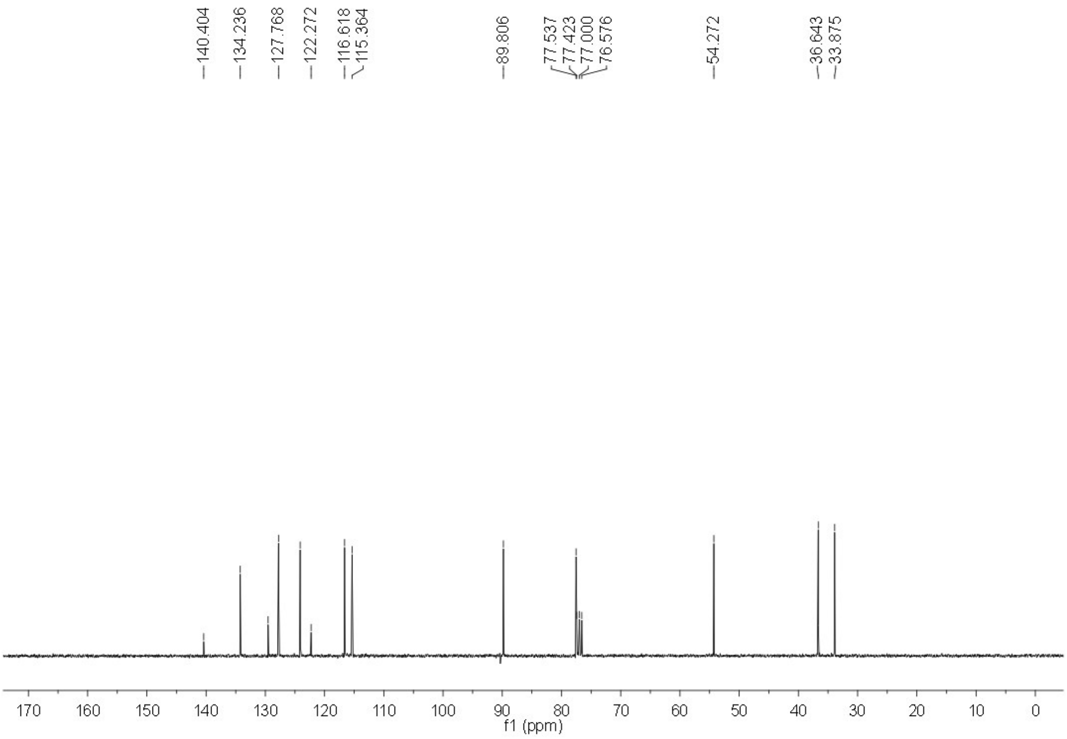

NMR spectrum of Compound **1d**

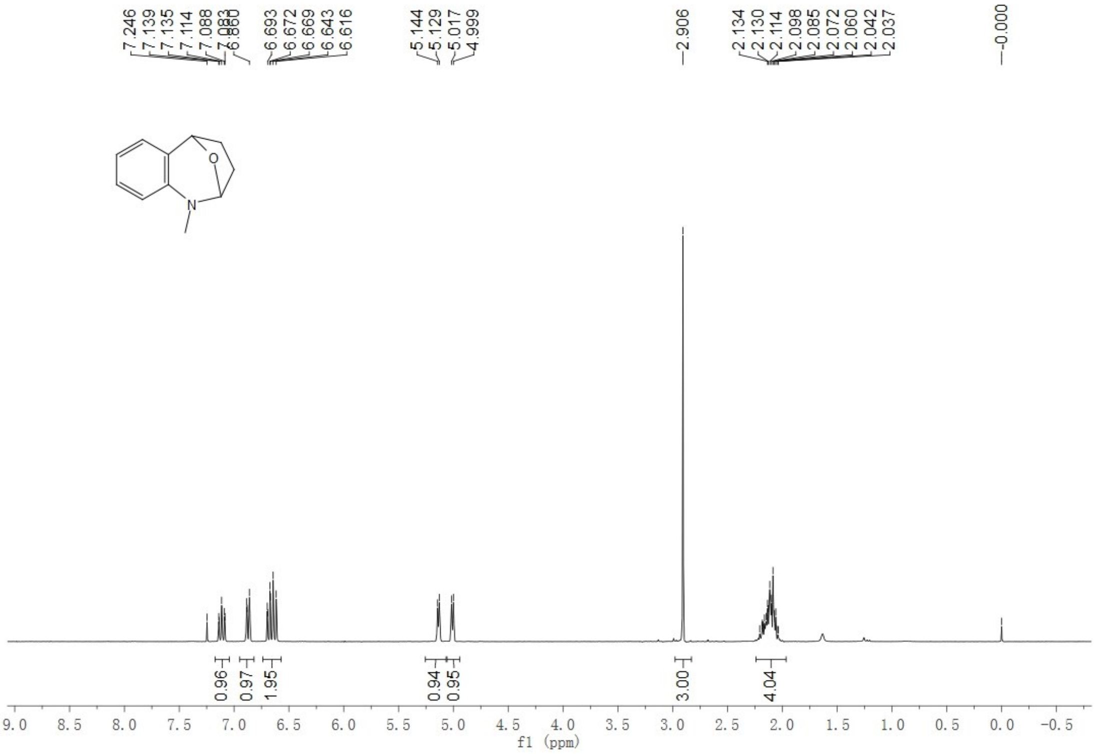

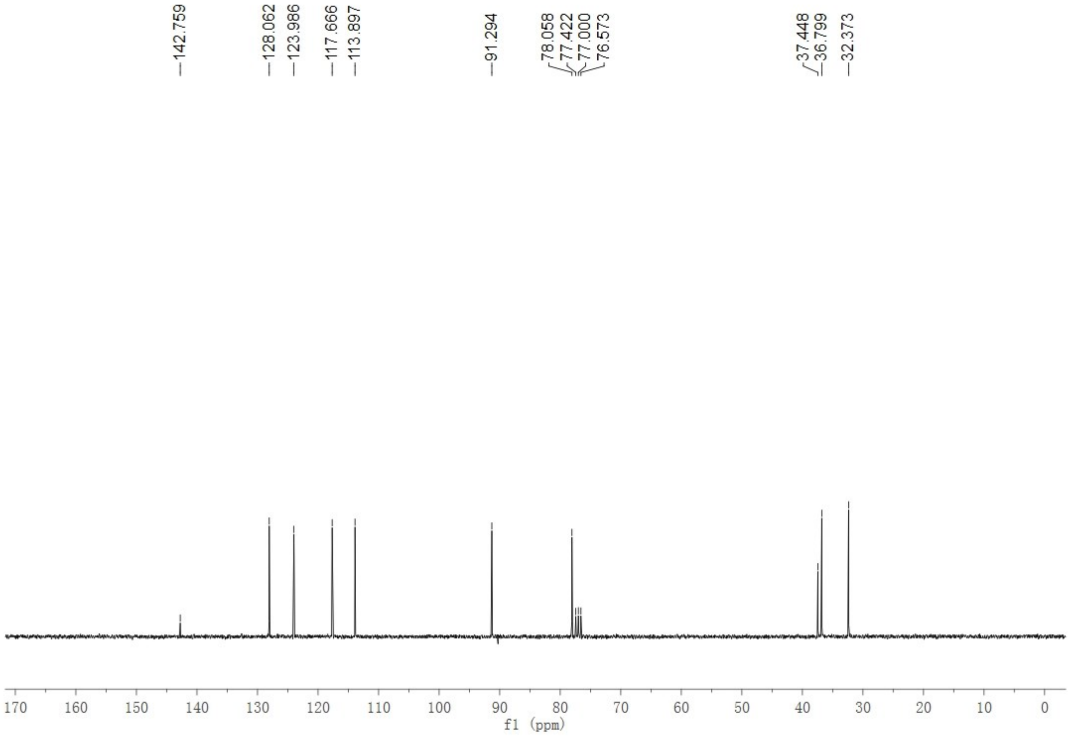

NMR spectrum of Compound **1e**

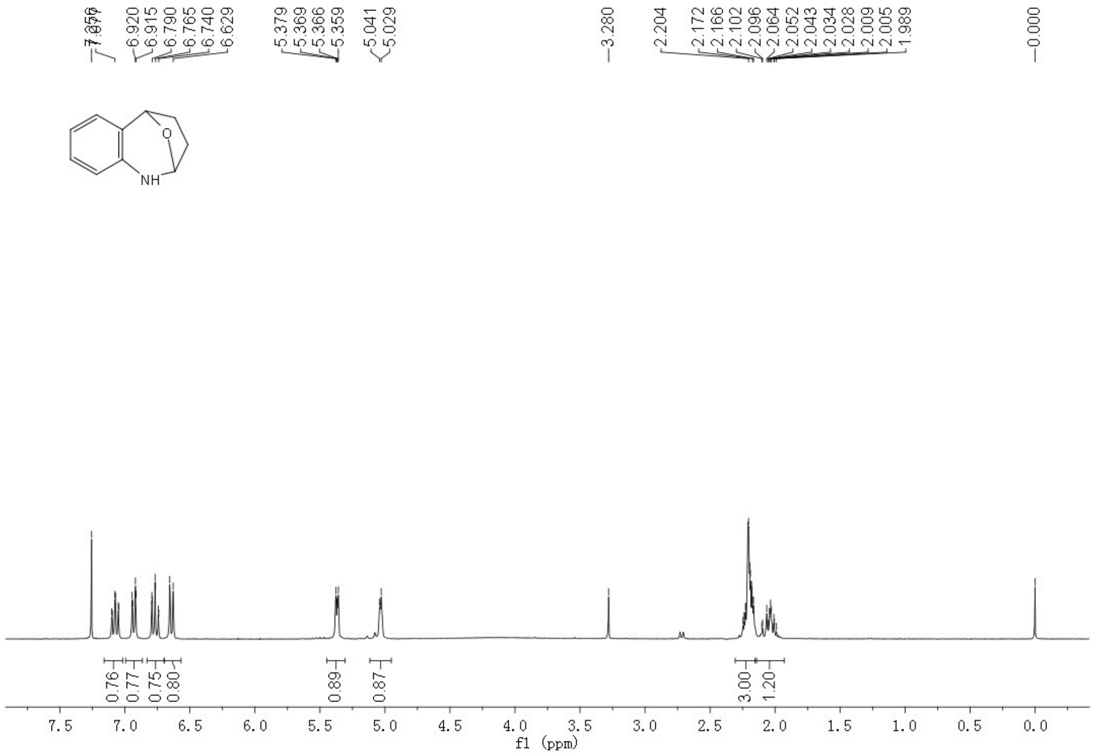

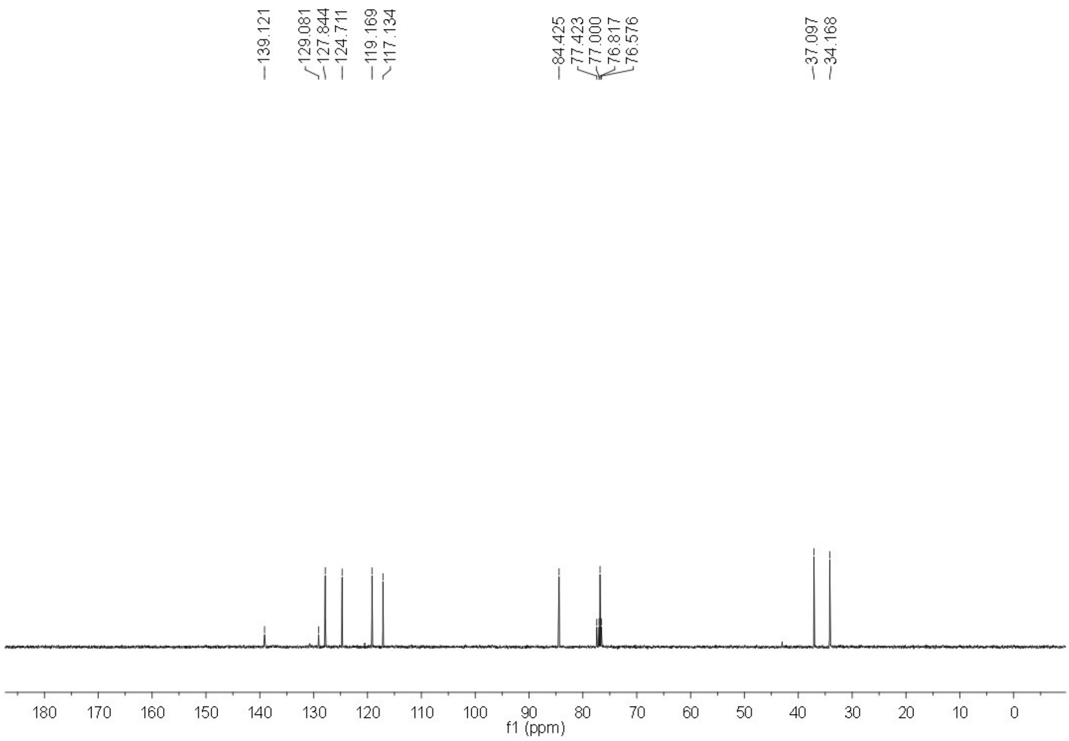

NMR spectrum of Compound syn-**2a**

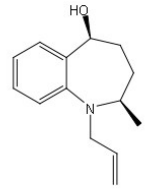

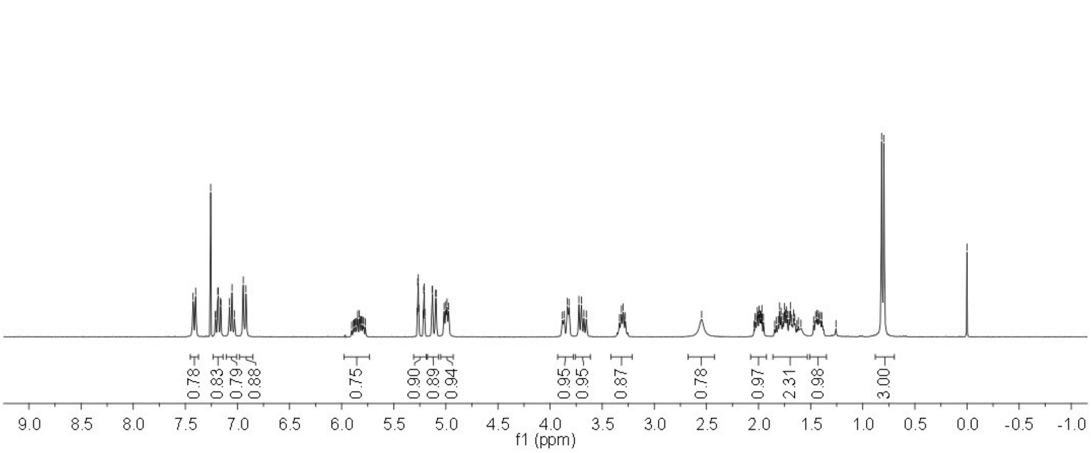

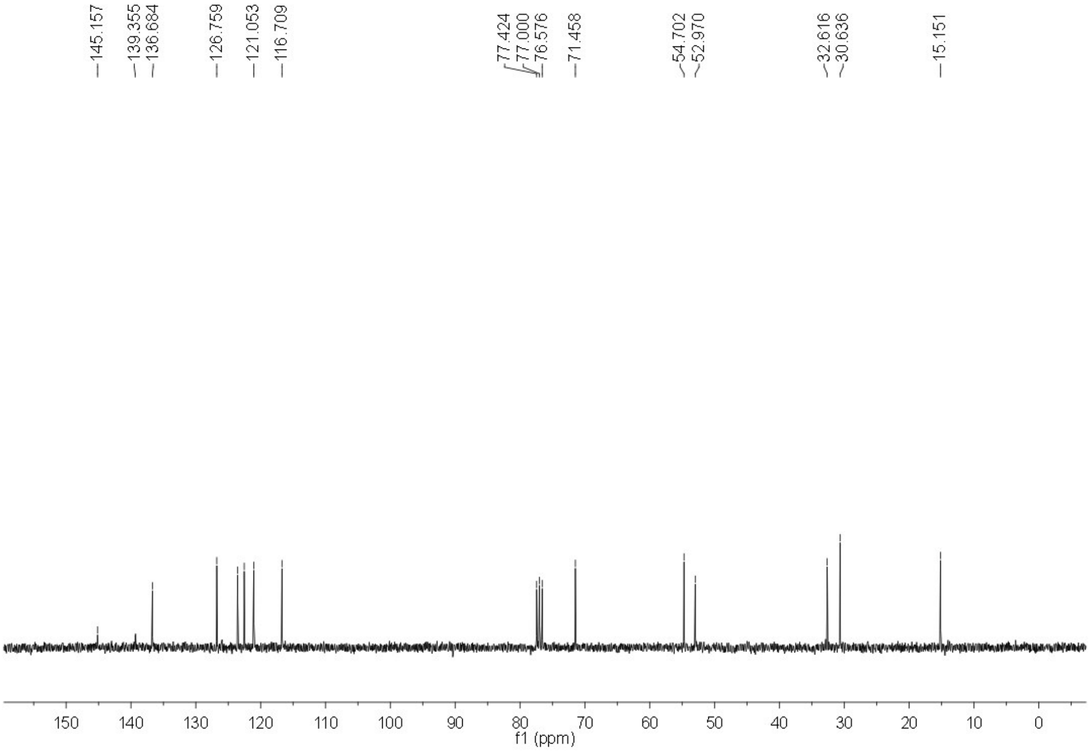

^{1}H NMR spectrum of Compound anti-**2a**

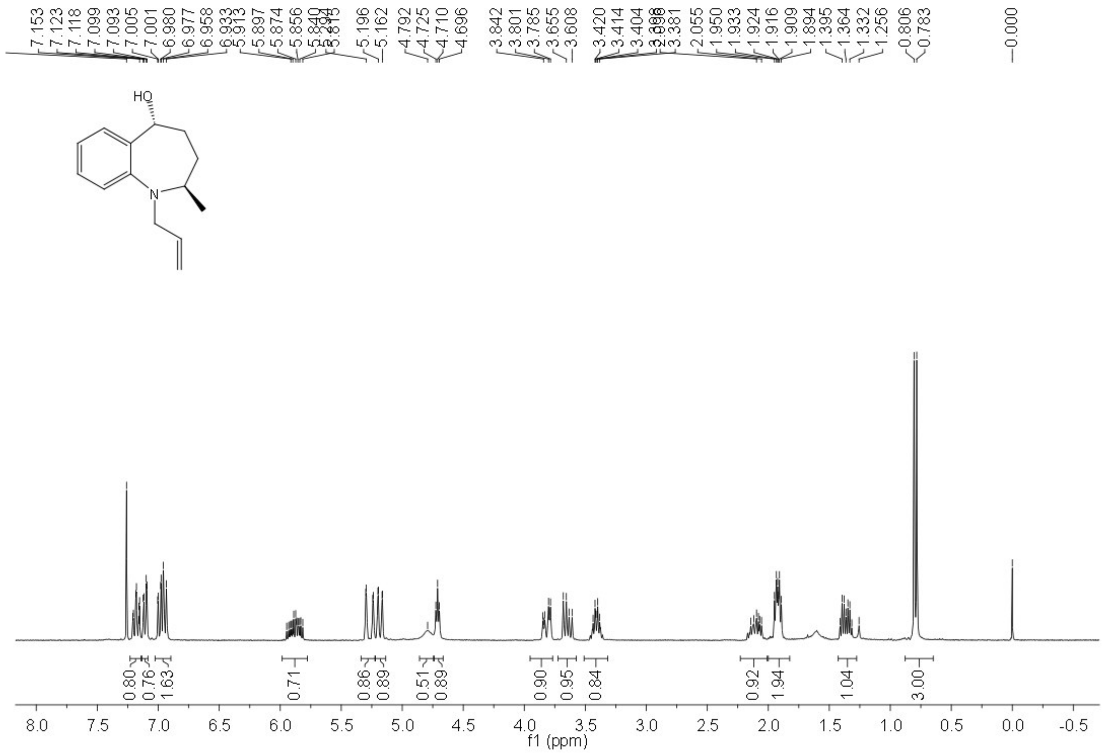

NMR spectrum of Compound **2b** (syn/anti = 91/9)

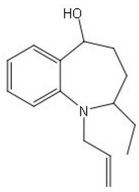

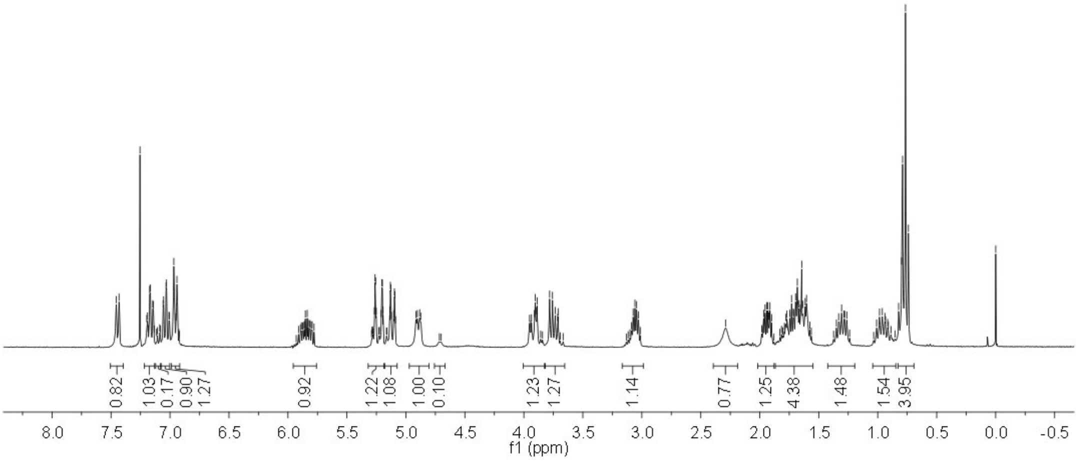

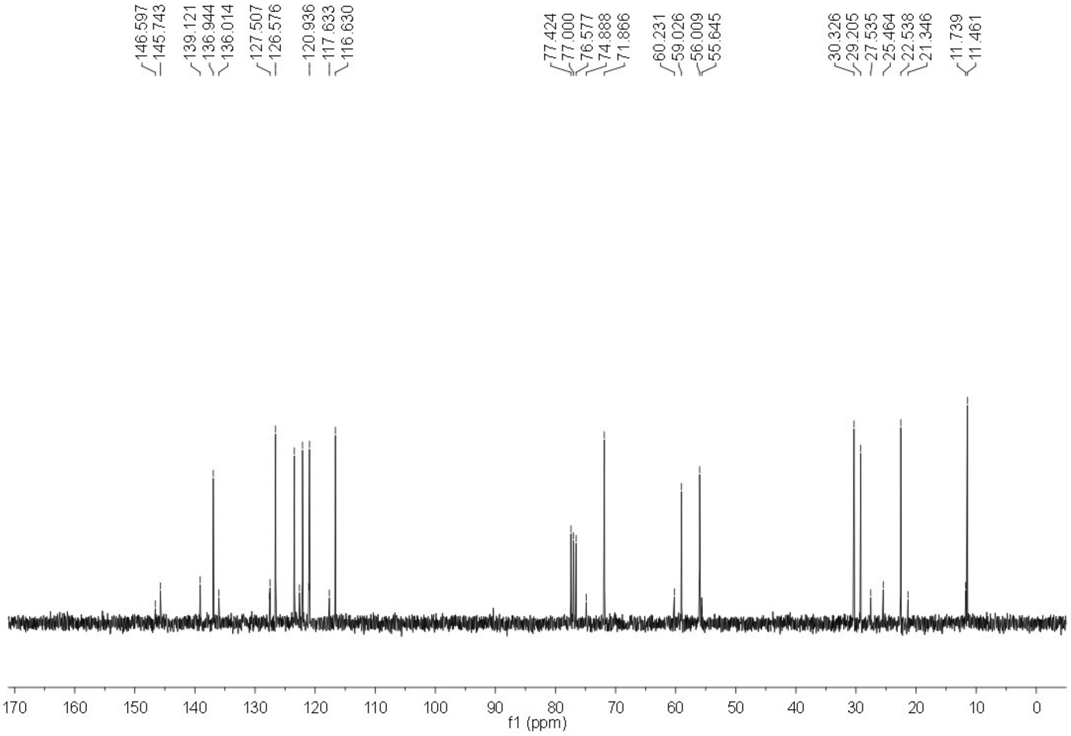

NMR spectrum of Compound **2c** (syn)

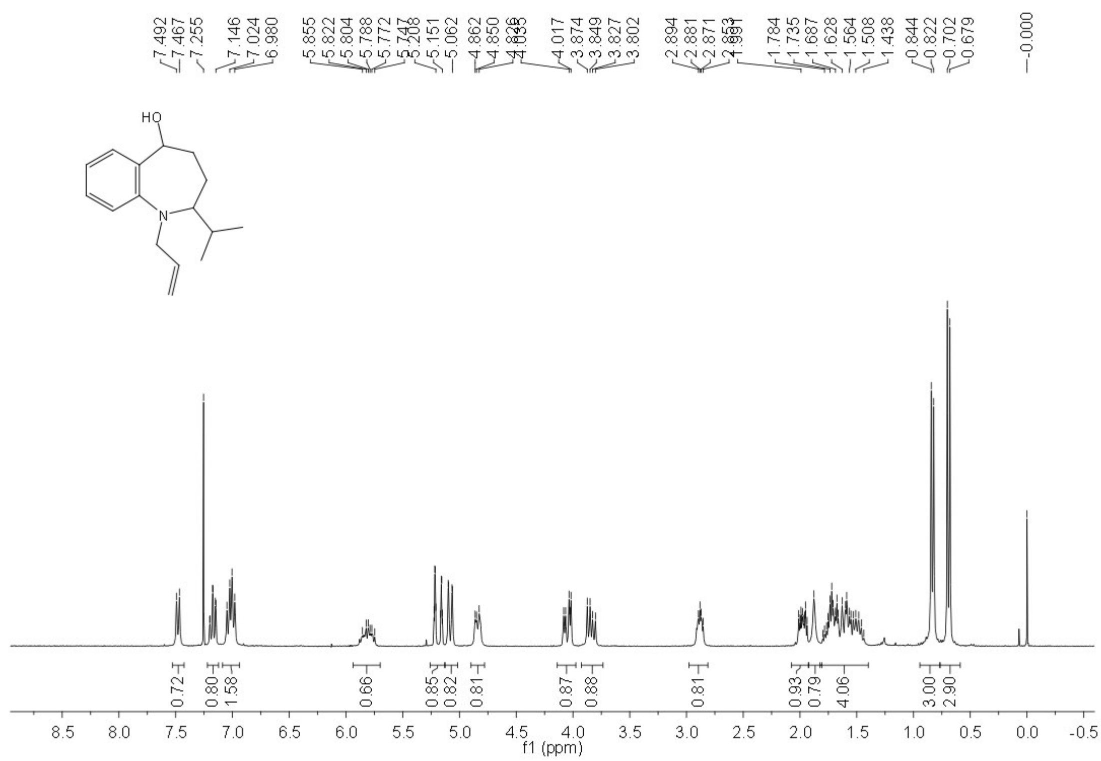

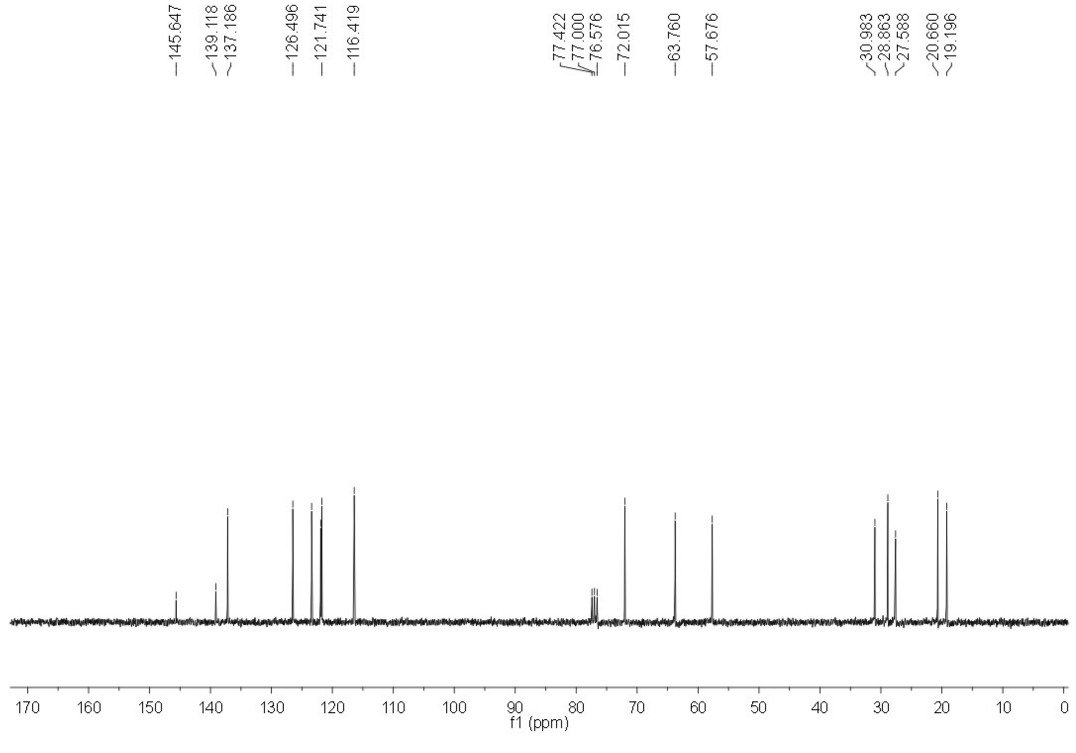

NMR spectrum of Compound **2d** (syn)

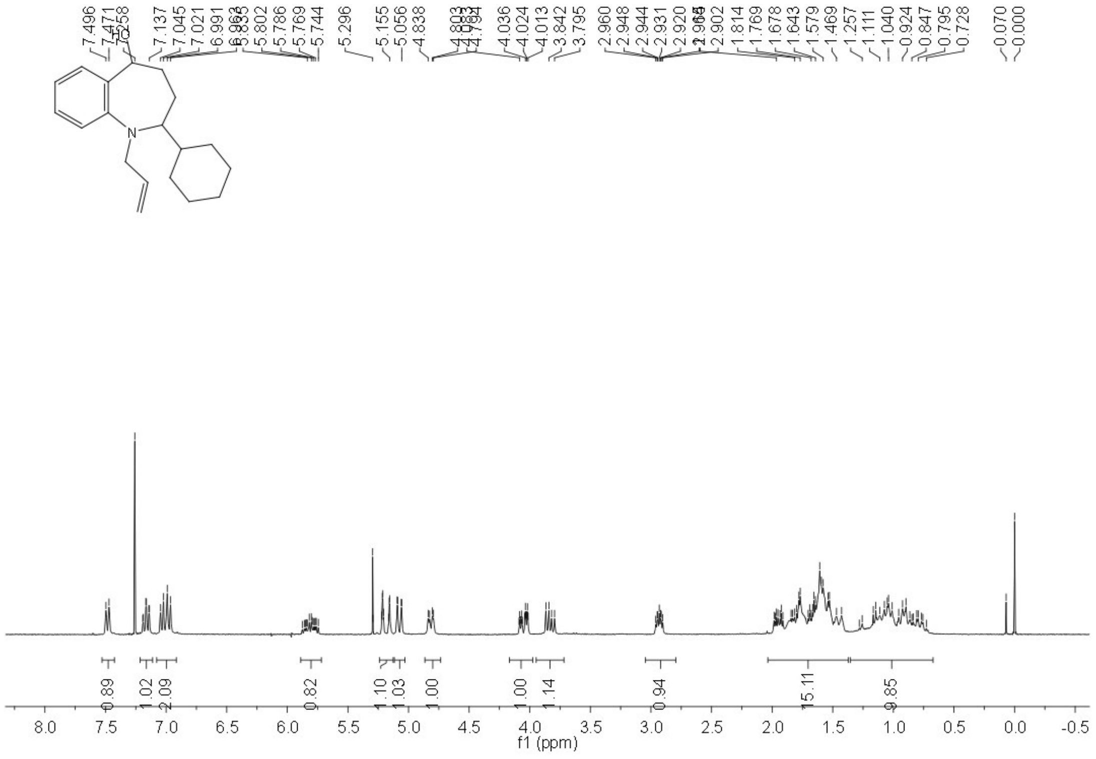

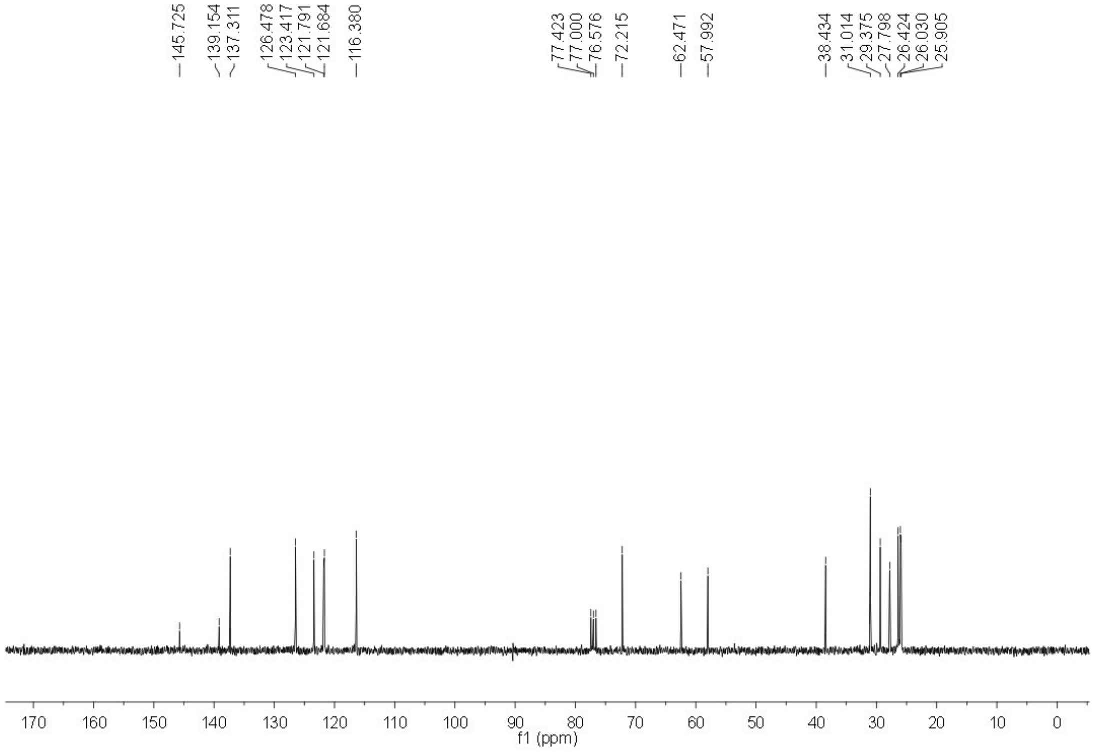

NMR spectrum of Compound **2e** (anti)

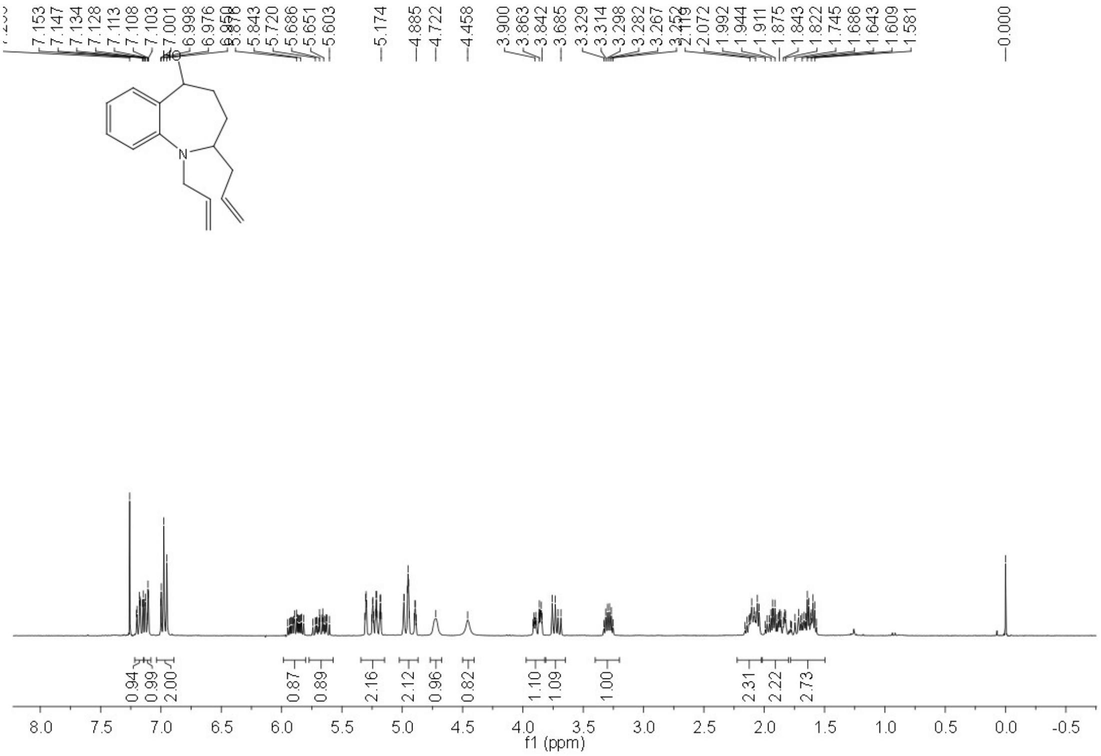

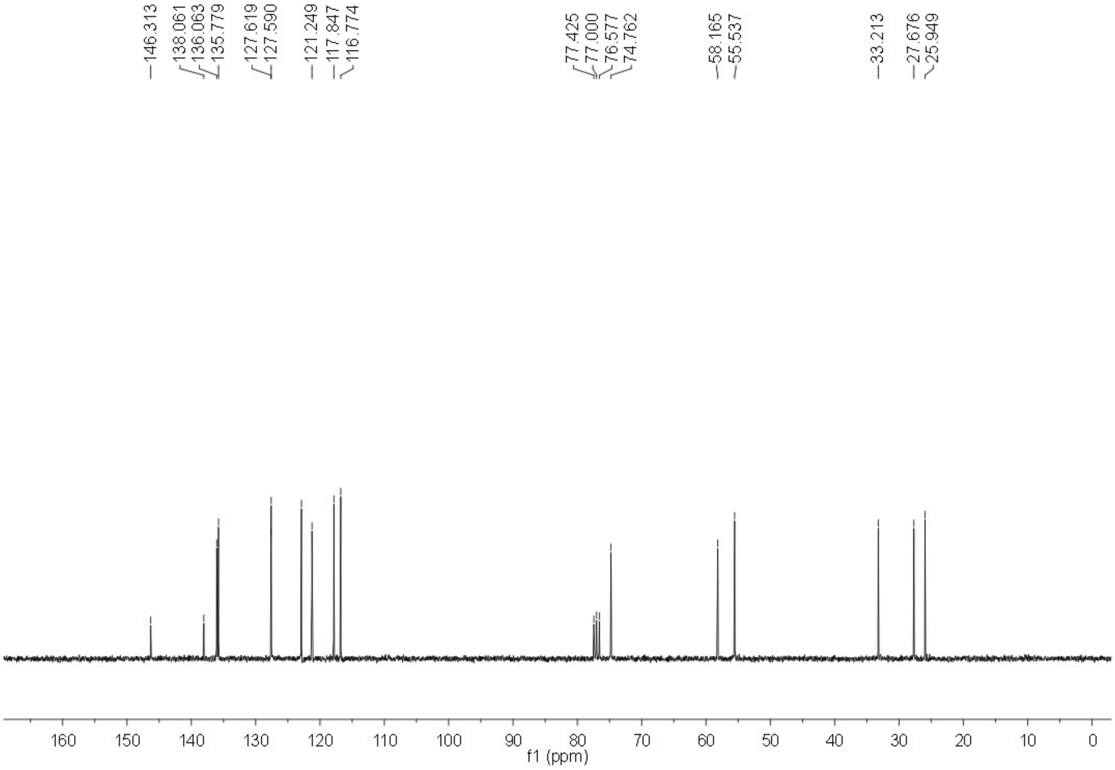

NMR spectrum of Compound **2f** (syn/anti = 95/5)

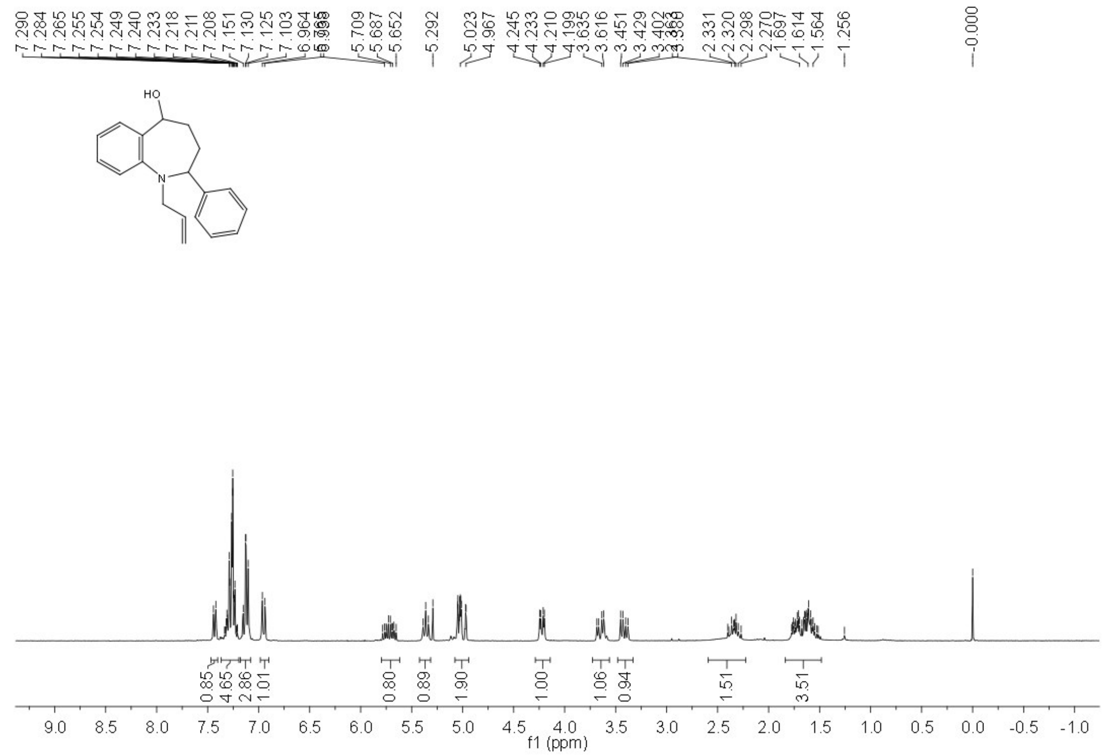

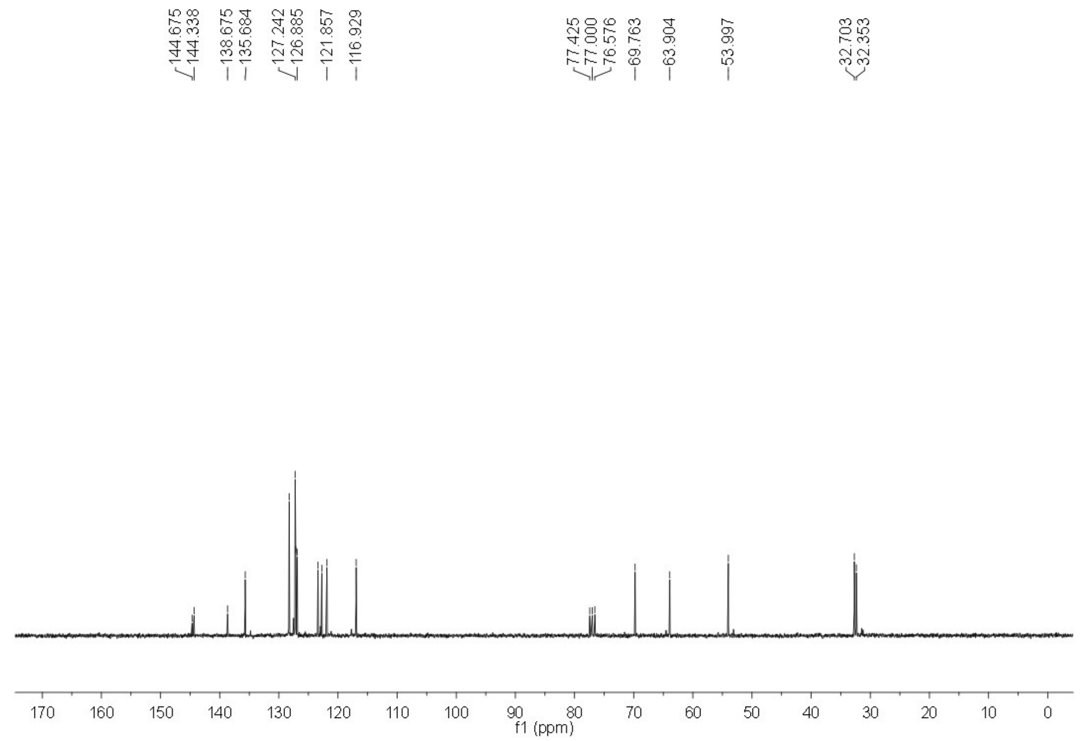

NMR spectrum of Compound **2g** (syn/anti = 88/12)

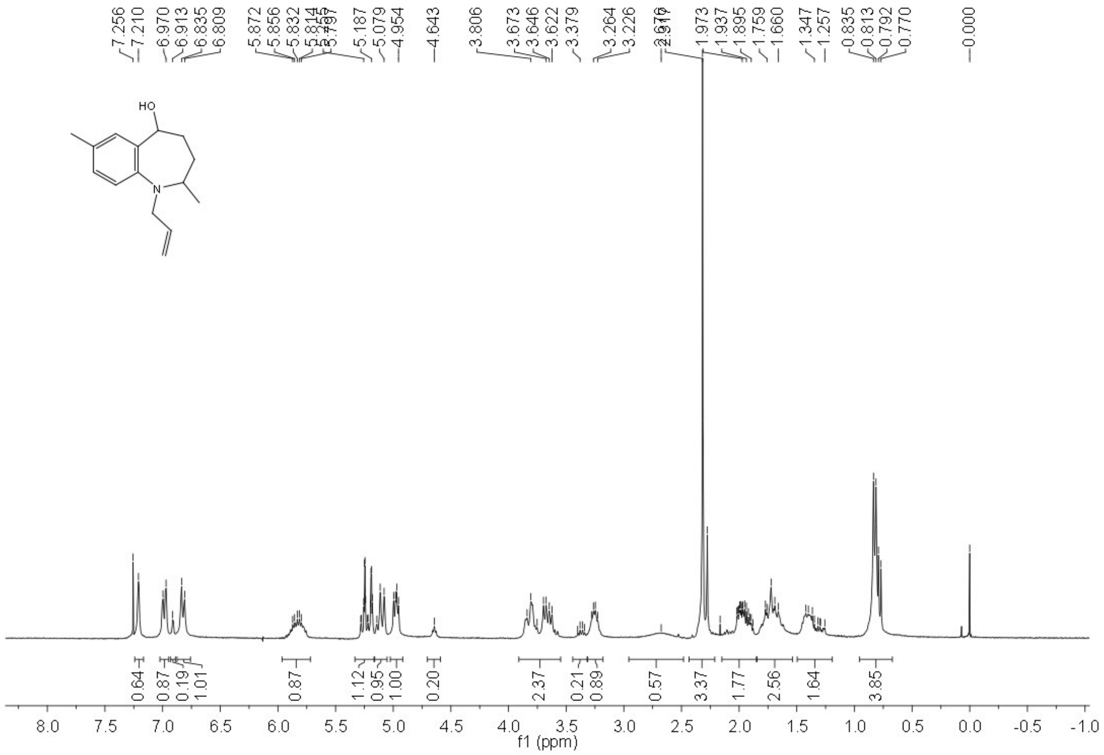

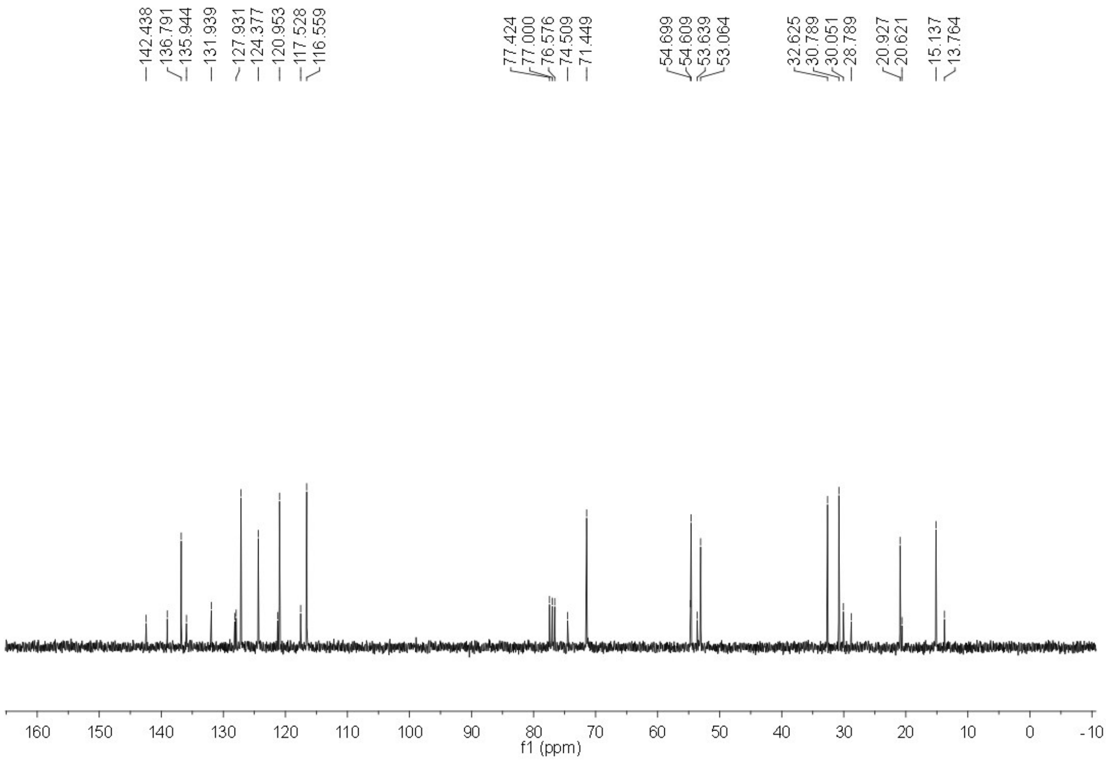

NMR spectrum of Compound **2h** (anti)

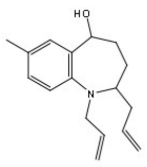

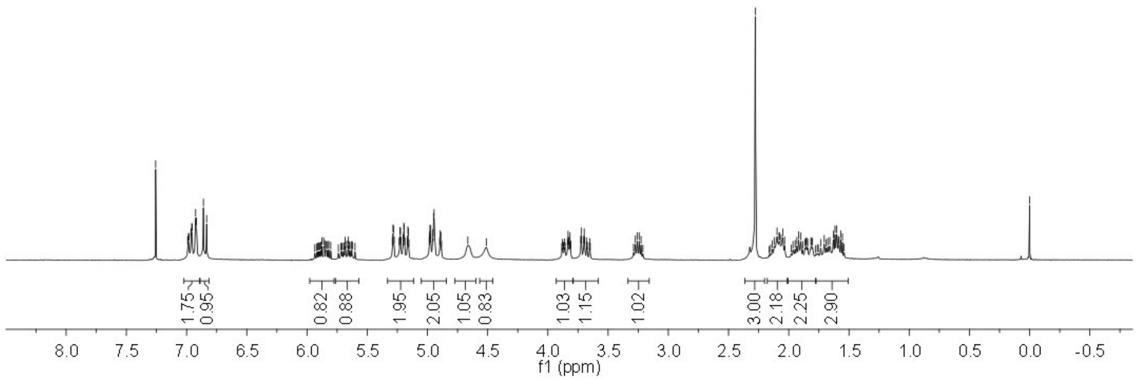

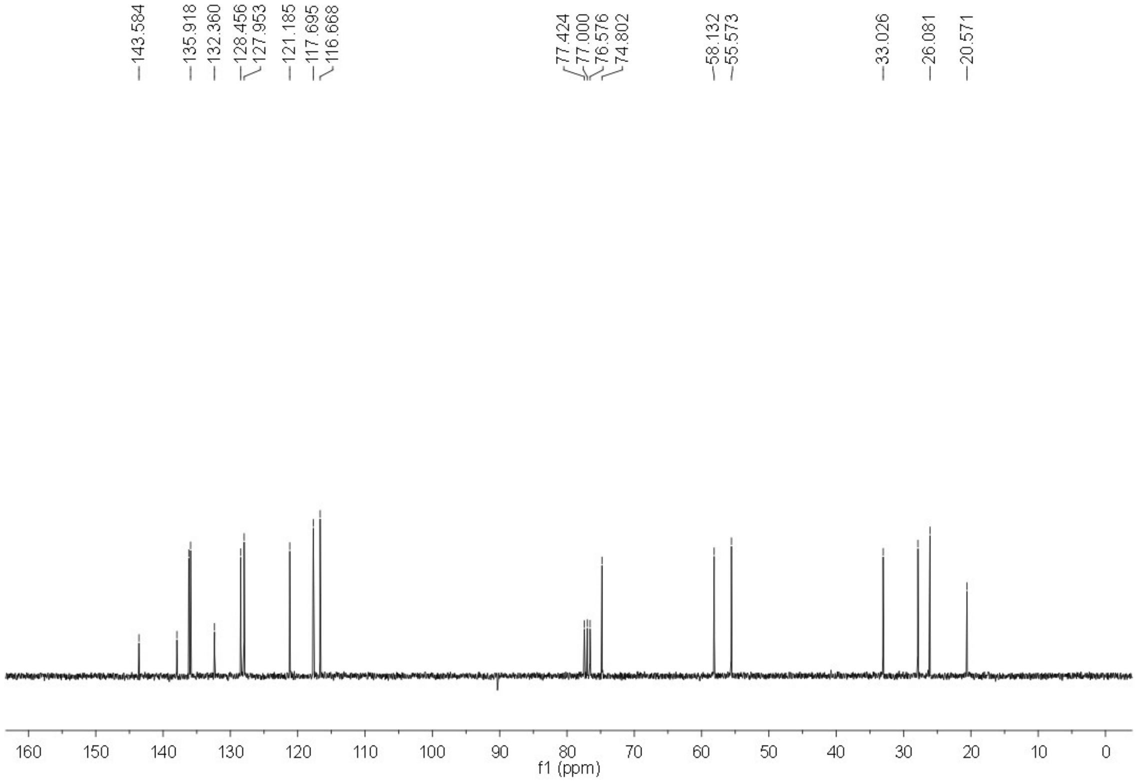

NMR spectrum of Compound **2i** (syn/anti = 67/33)

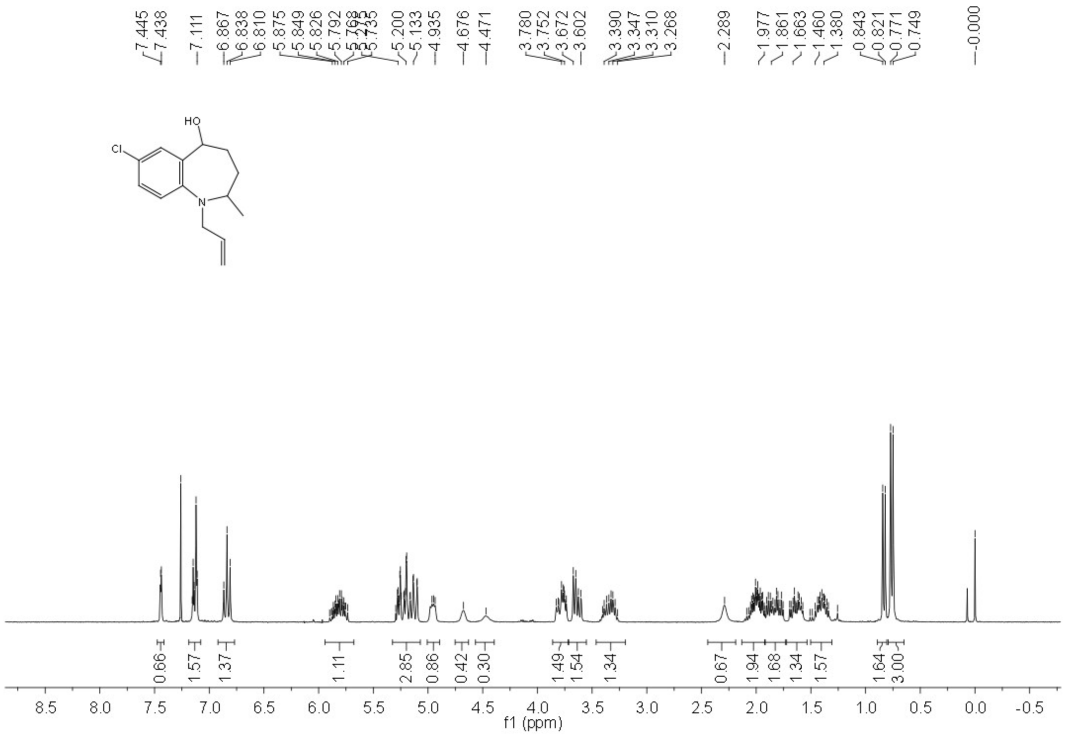

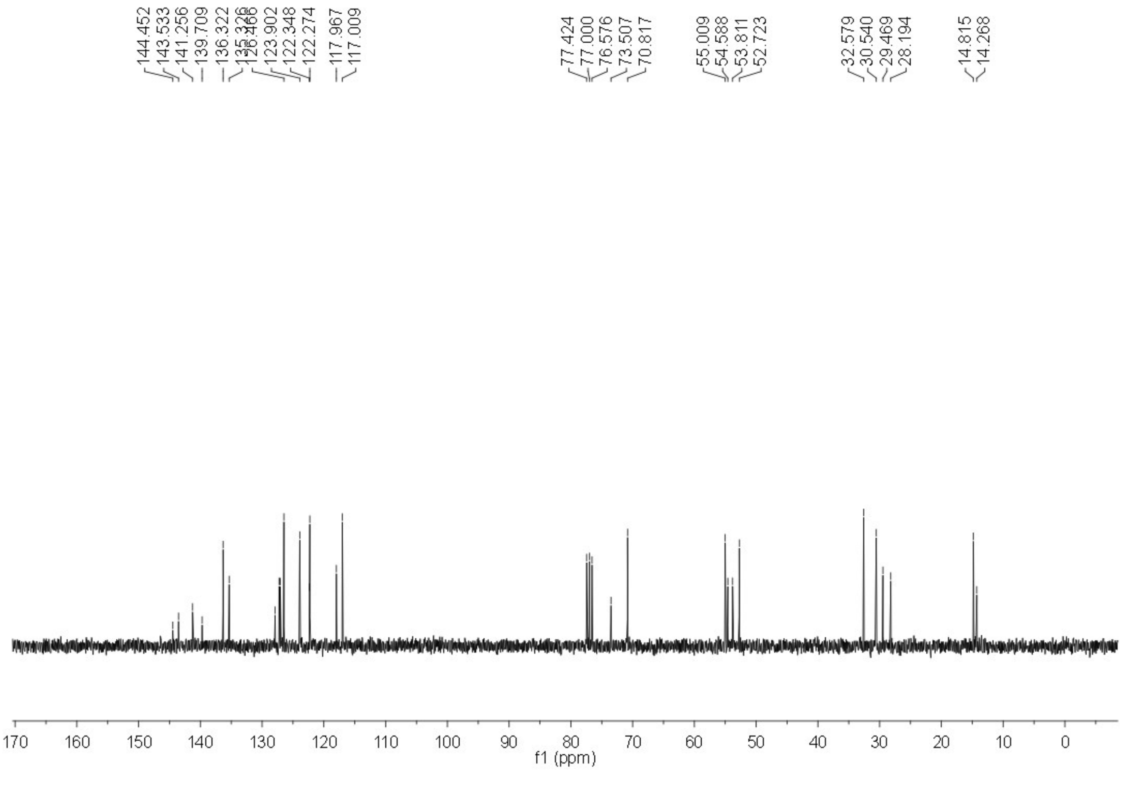

NMR spectrum of Compound **2j** (anti)

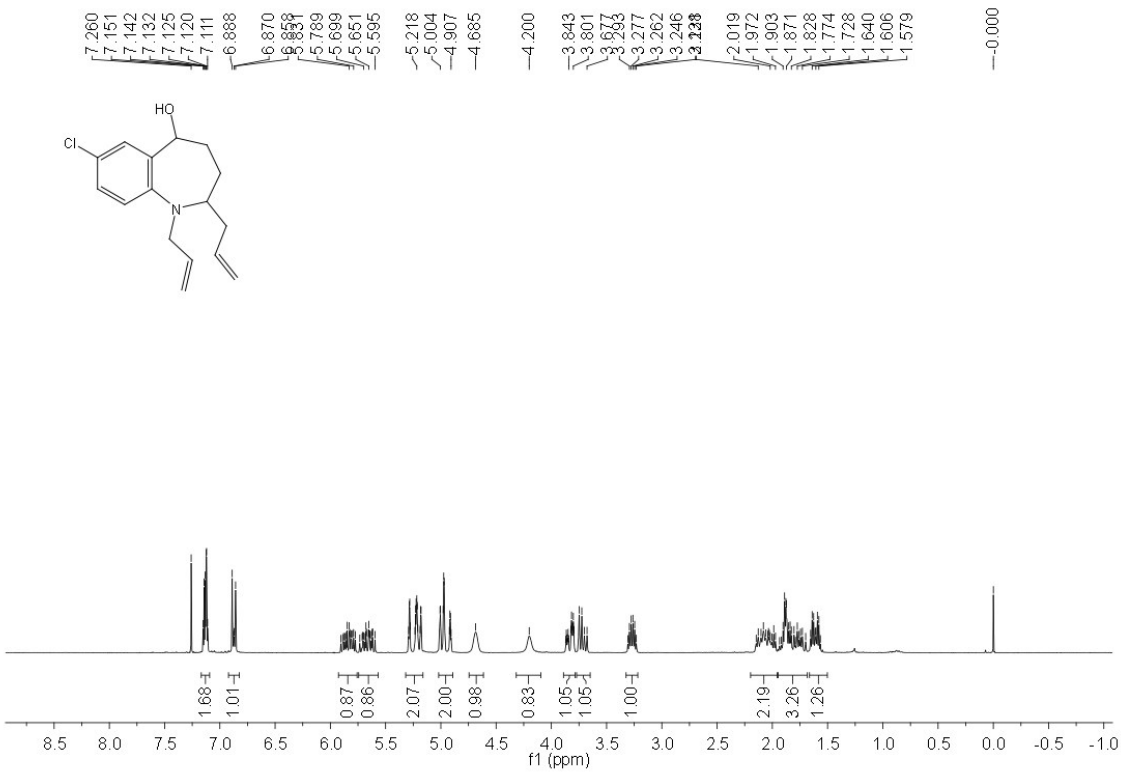

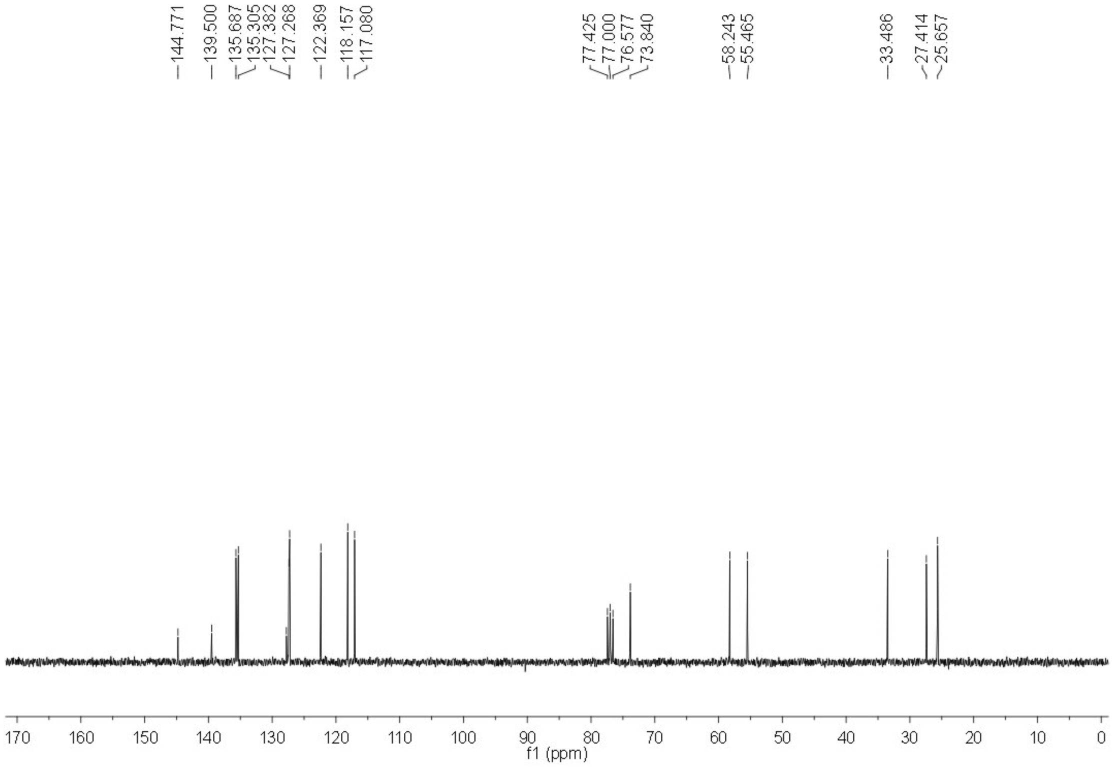

NMR spectrum of Compound **2k** (syn/anti = 68/32)

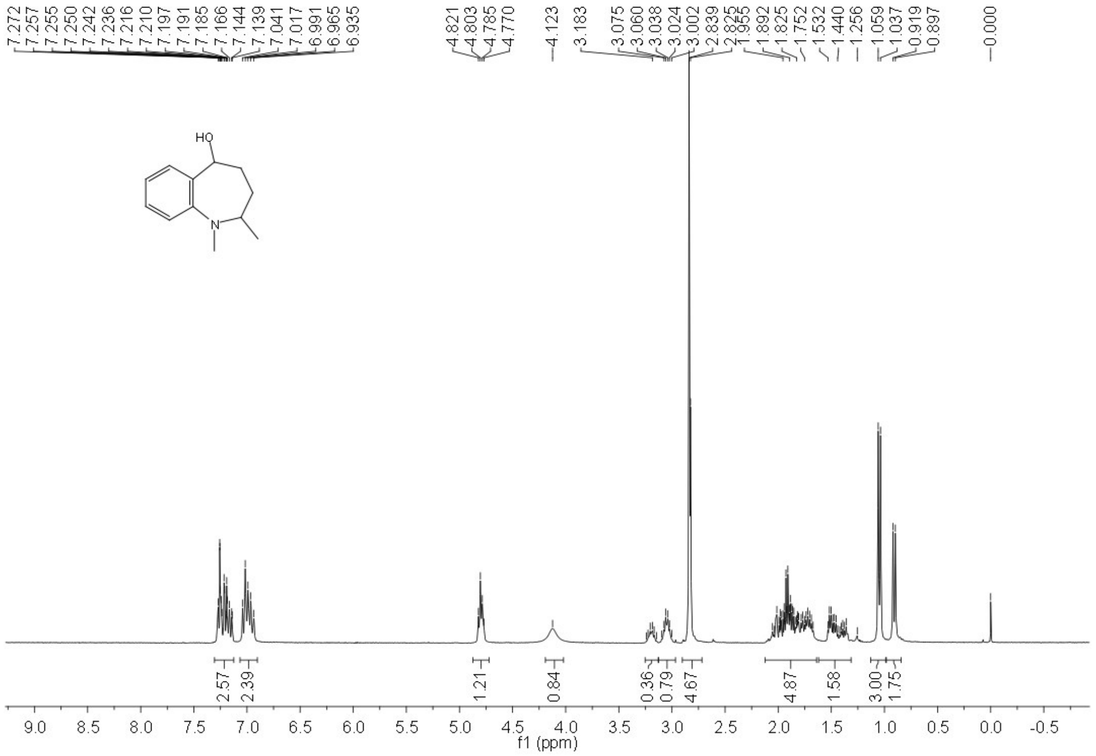

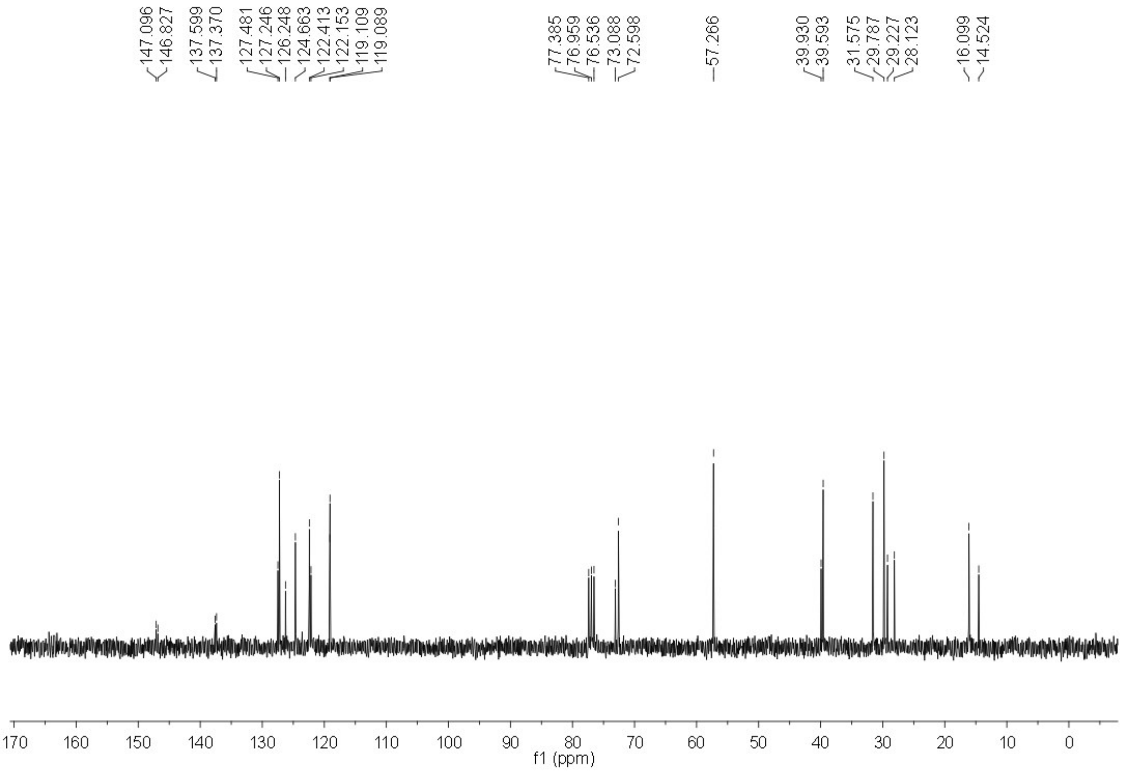

NMR spectrum of Compound **2l** (syn)

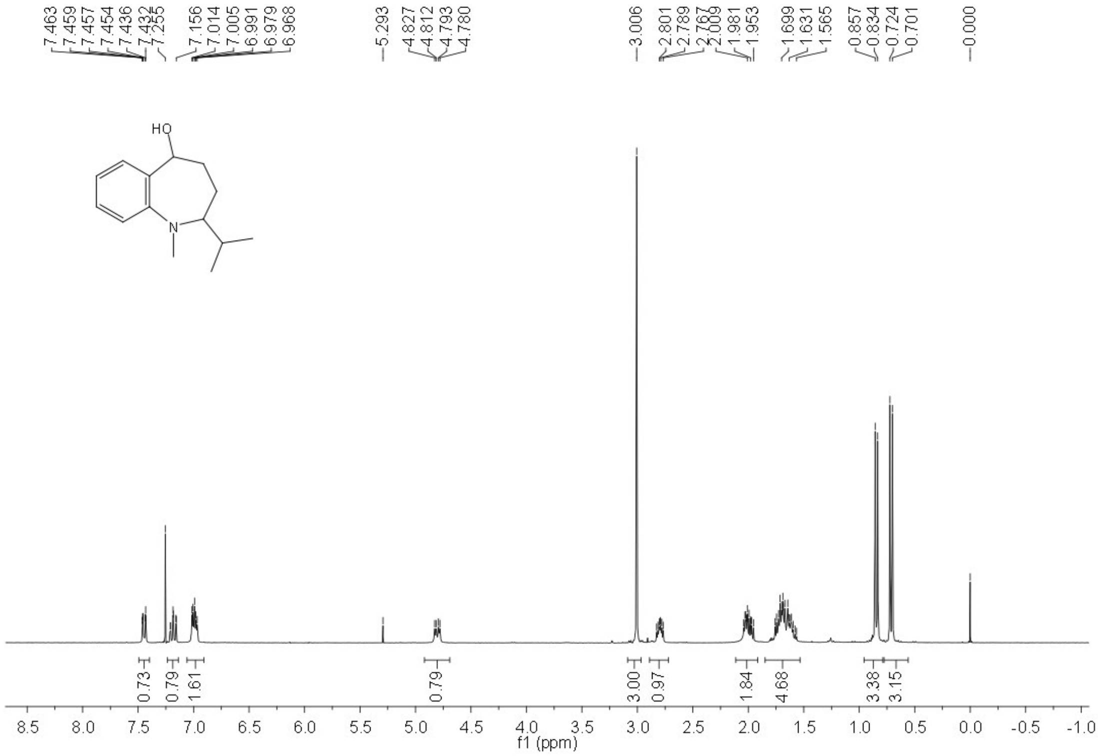

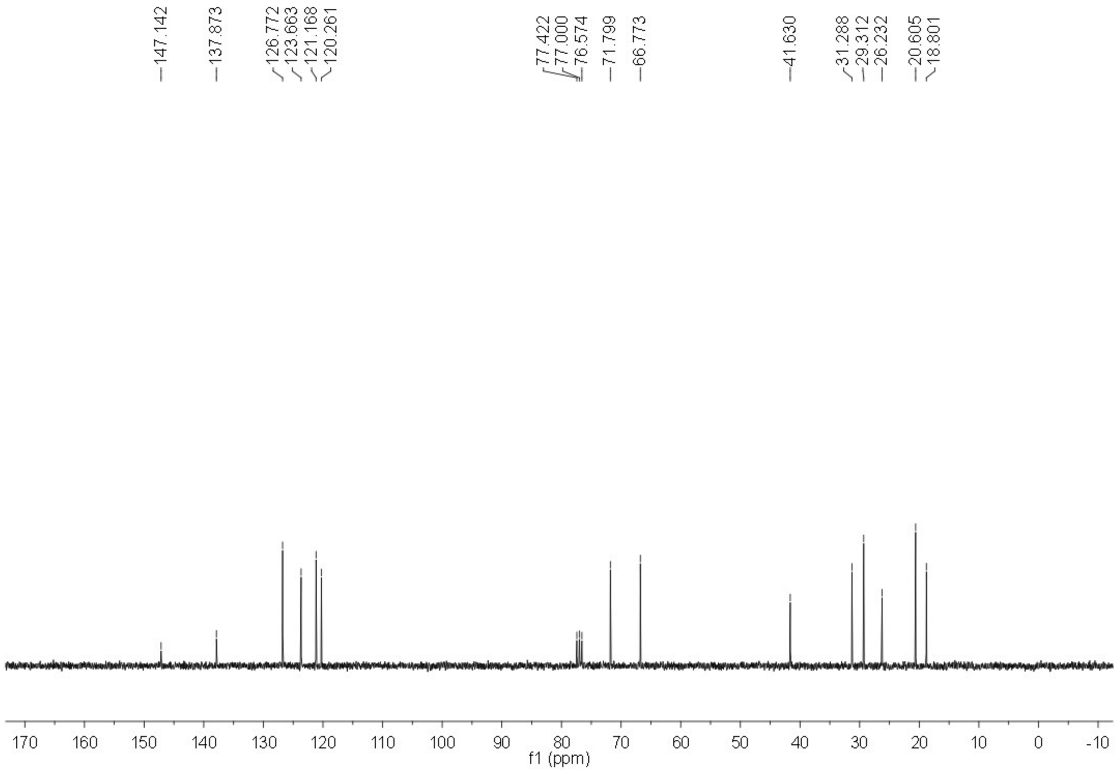

NMR spectrum of Compound **2m** (syn/anti = 91/9)

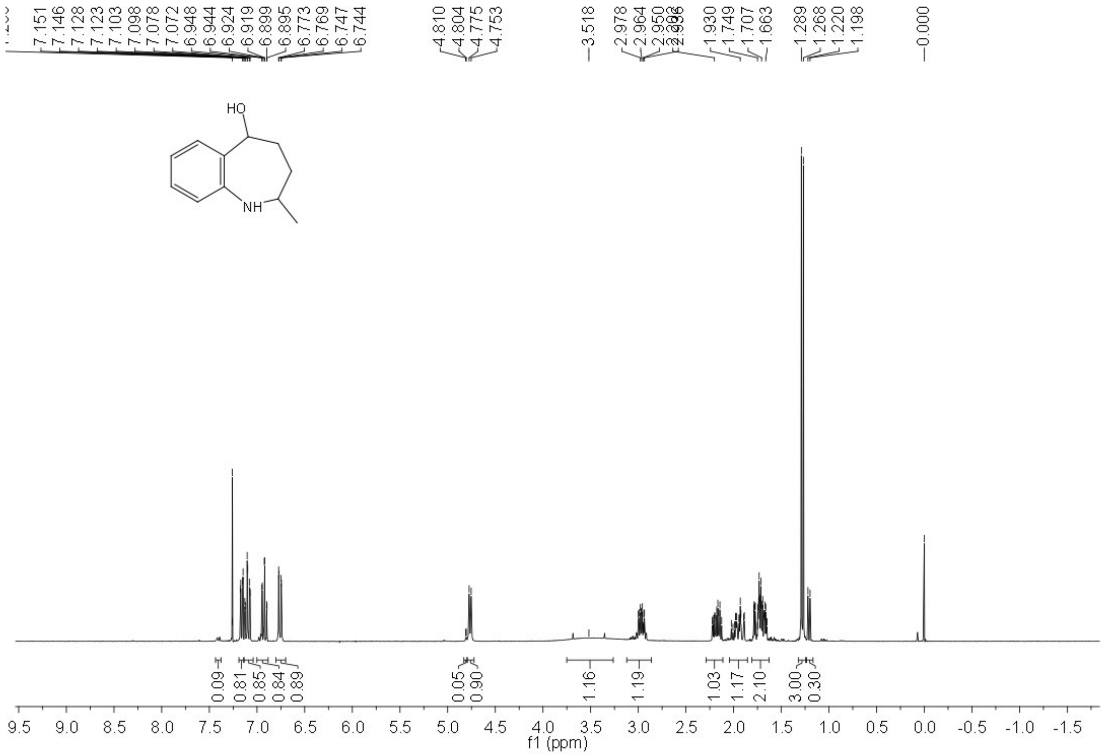

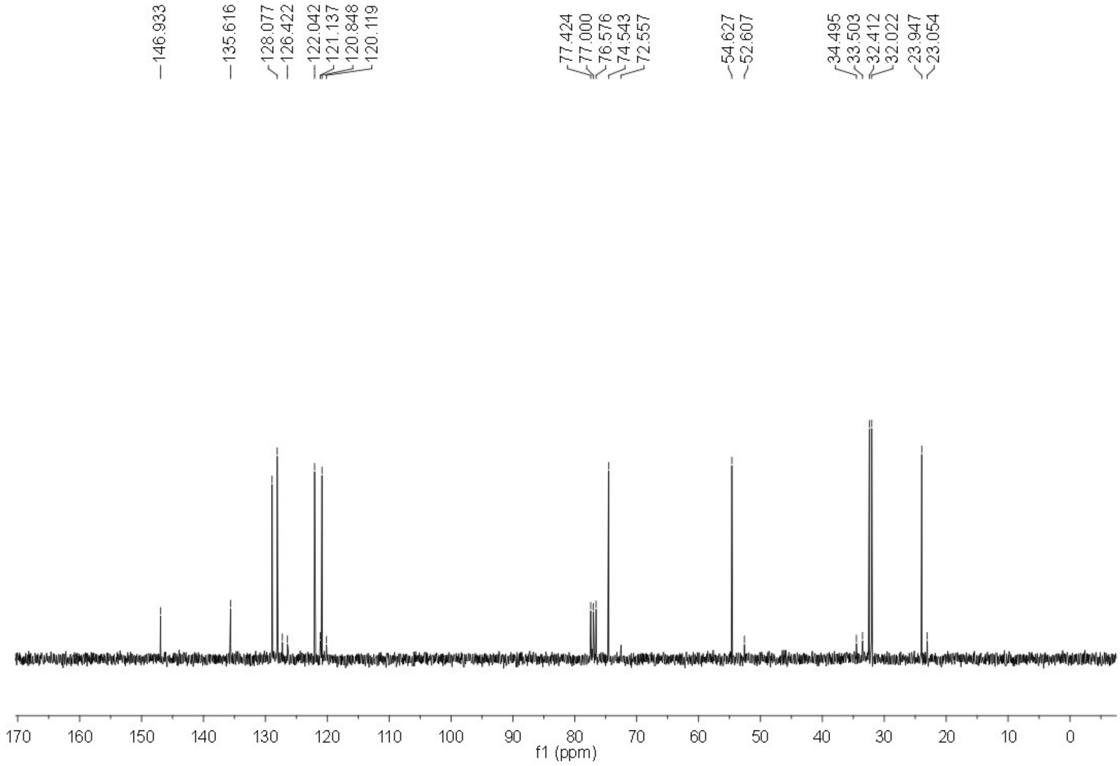

NMR spectrum of Compound syn-**2m**

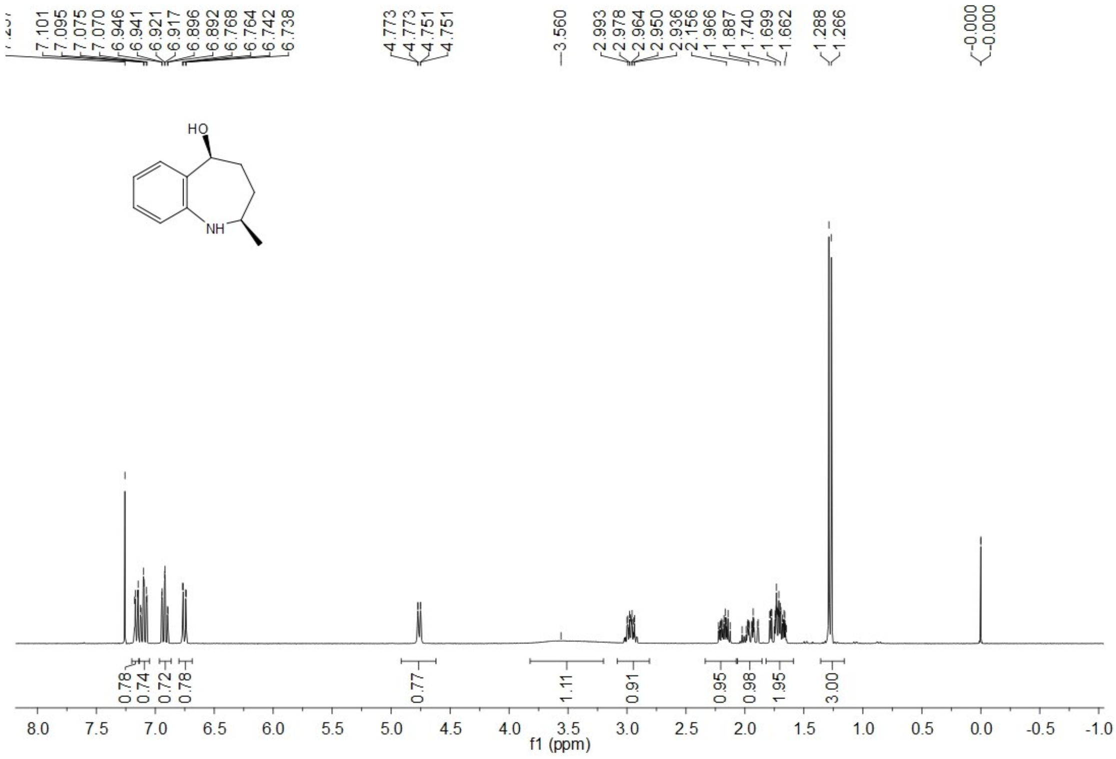

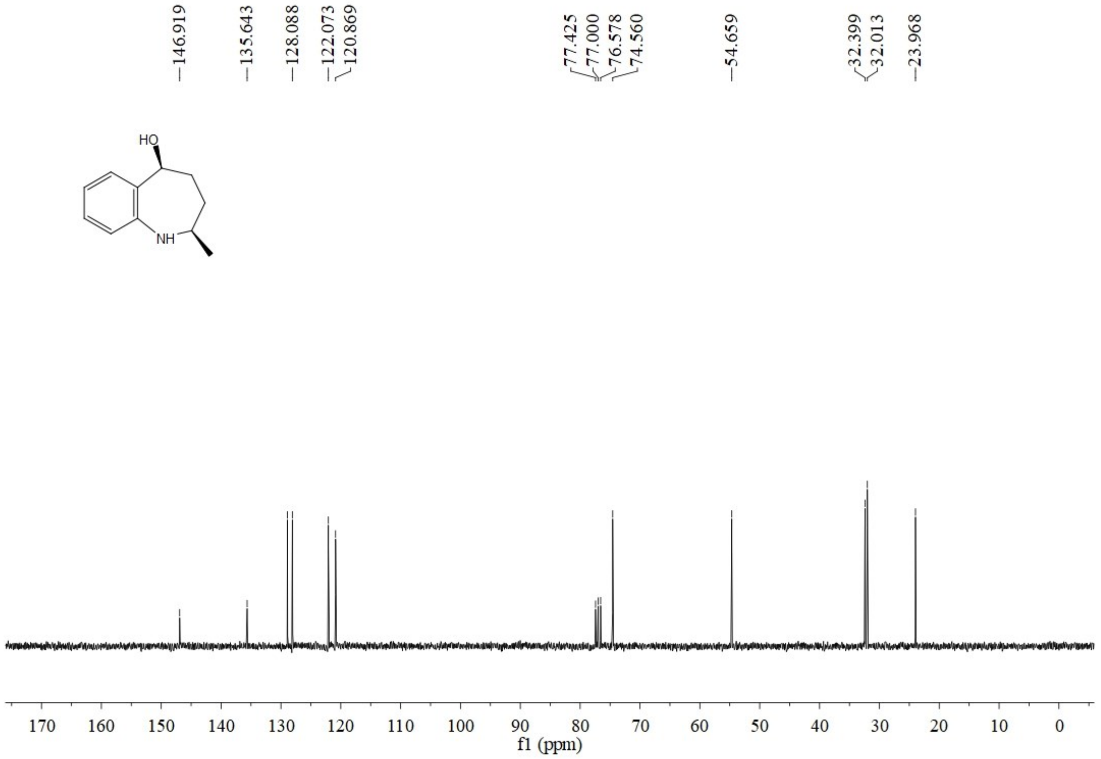

NMR spectrum of Compound **2n** (syn/anti = 75/25)

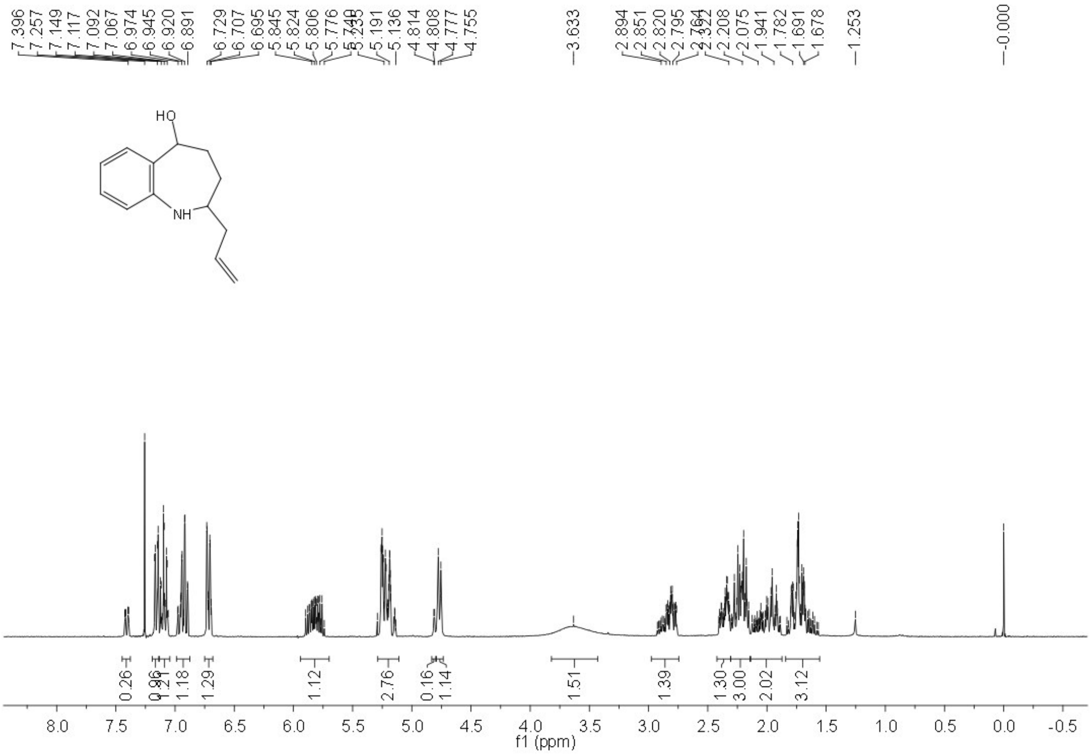

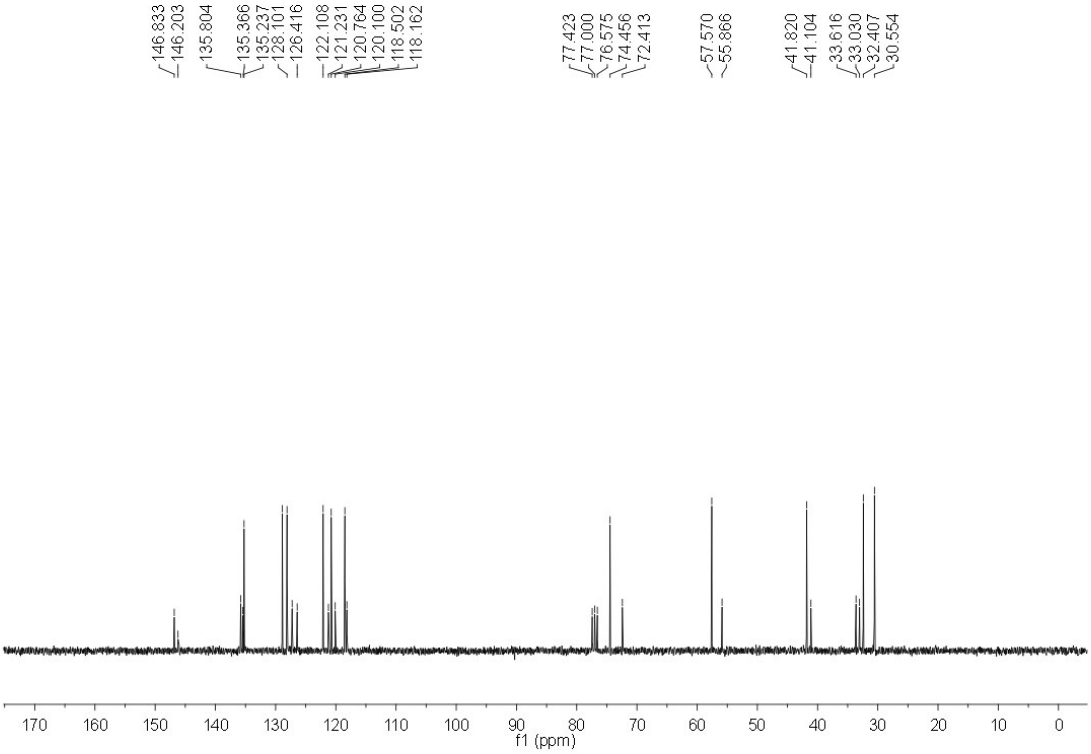

NMR spectrum of Compound **3a**

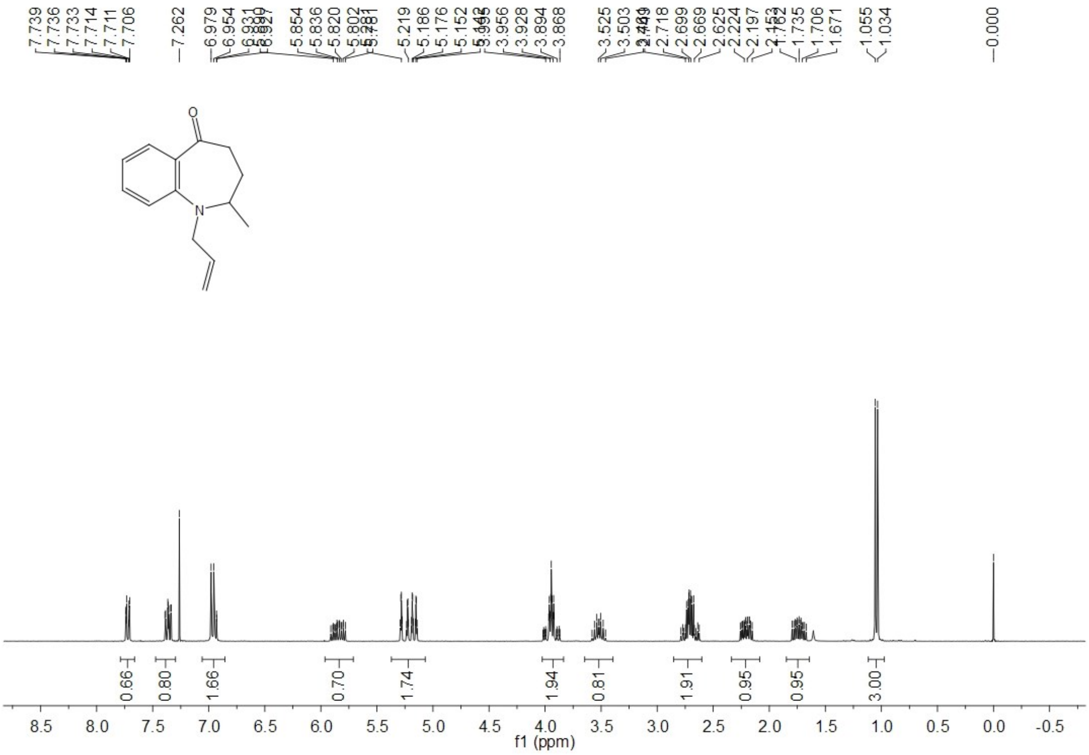

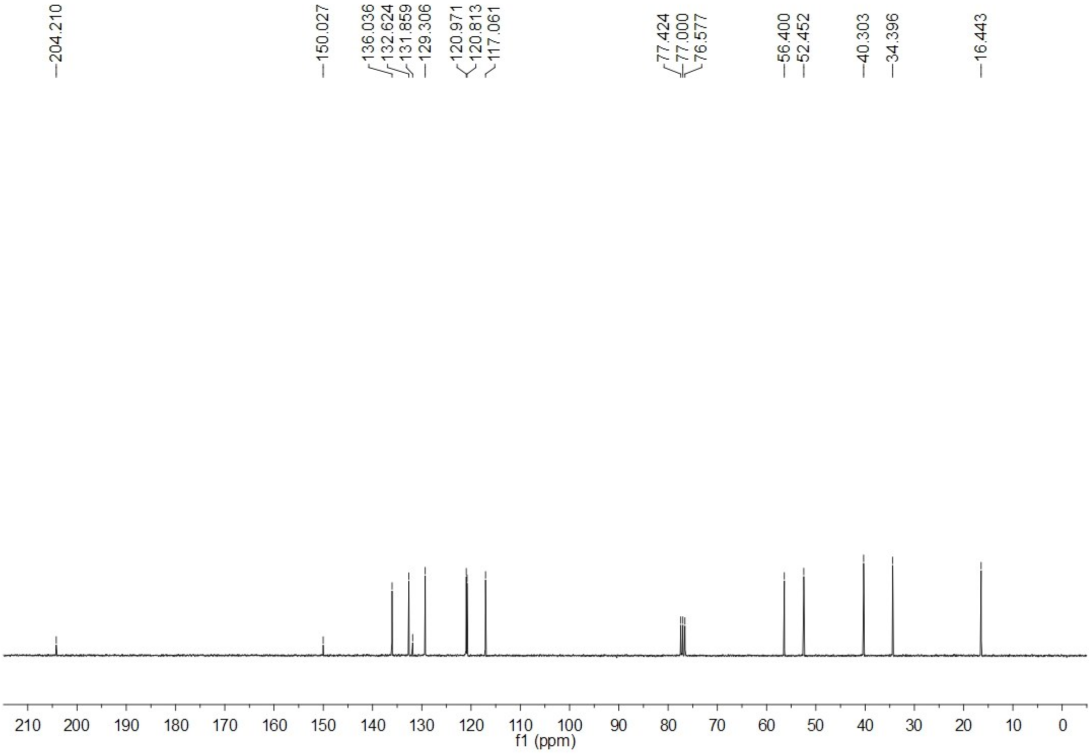

NMR spectrum of Compound **3b**

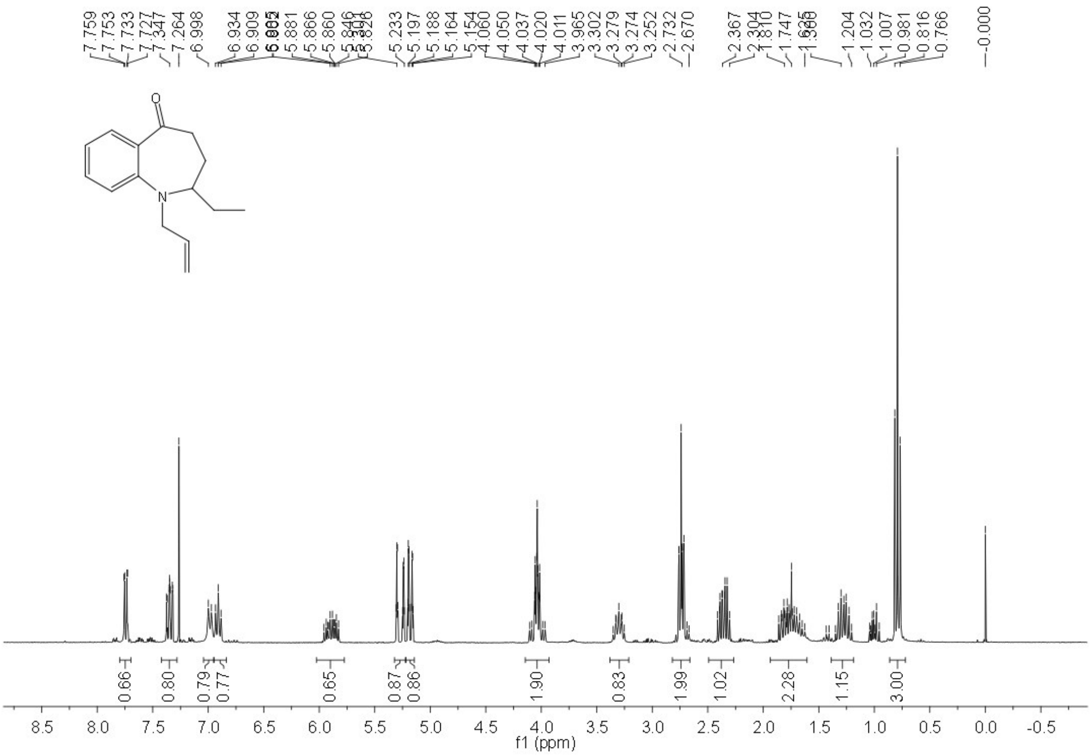

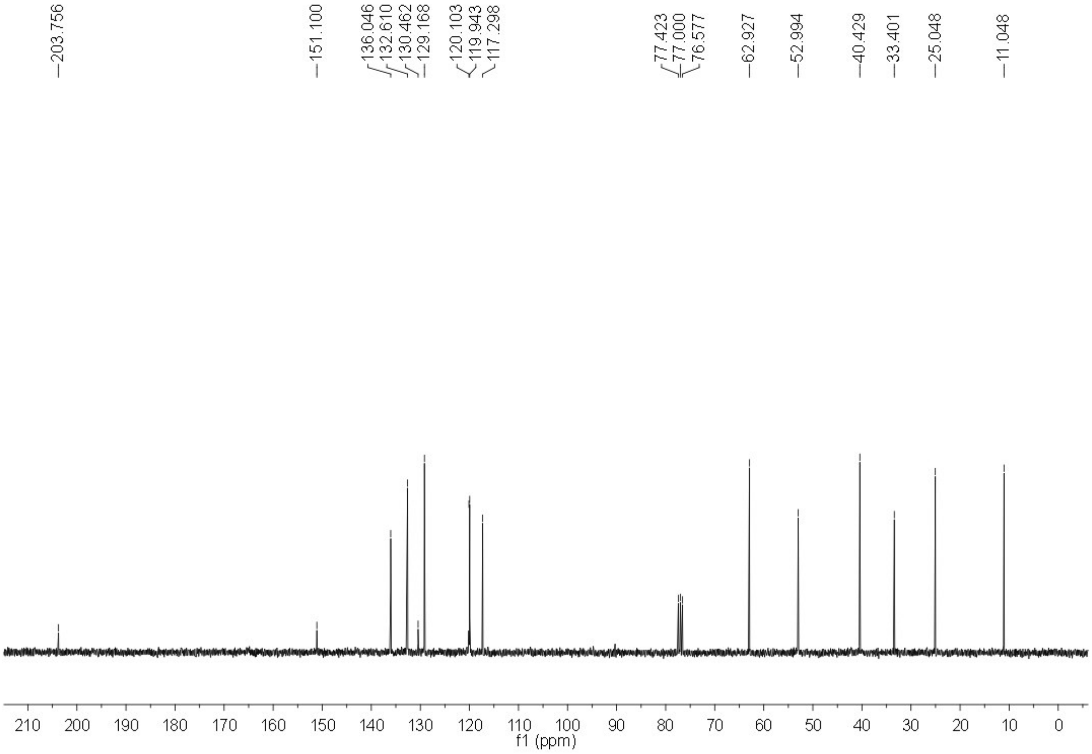

NMR spectrum of Compound **3c**

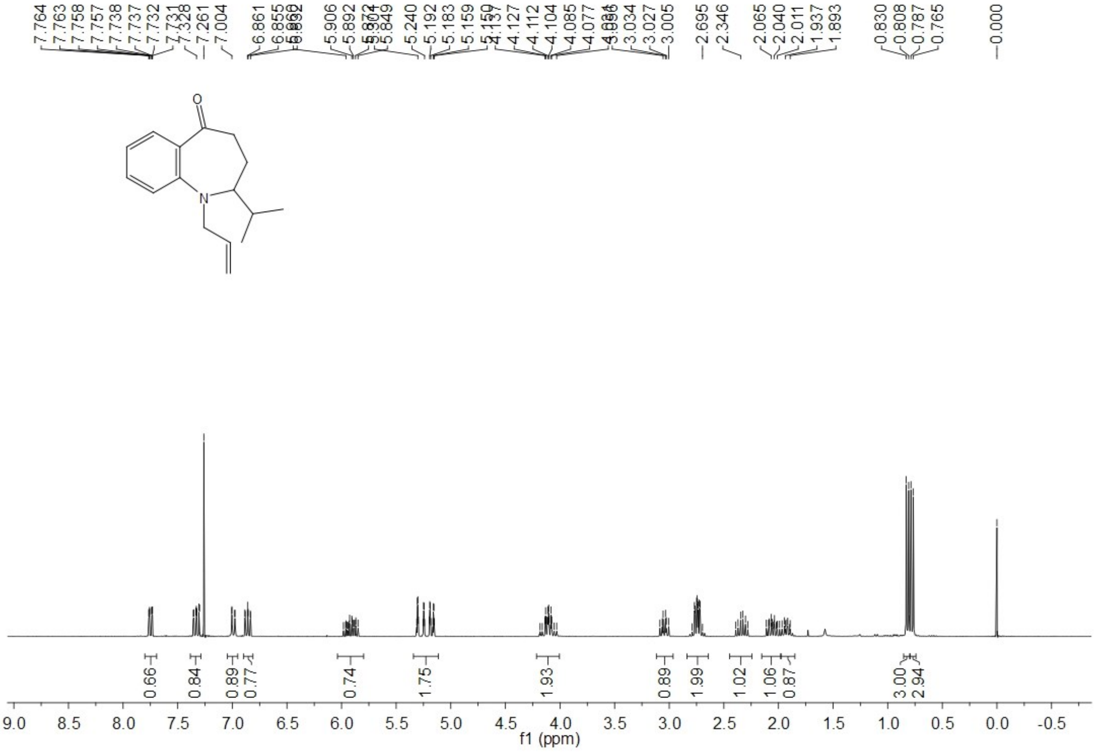

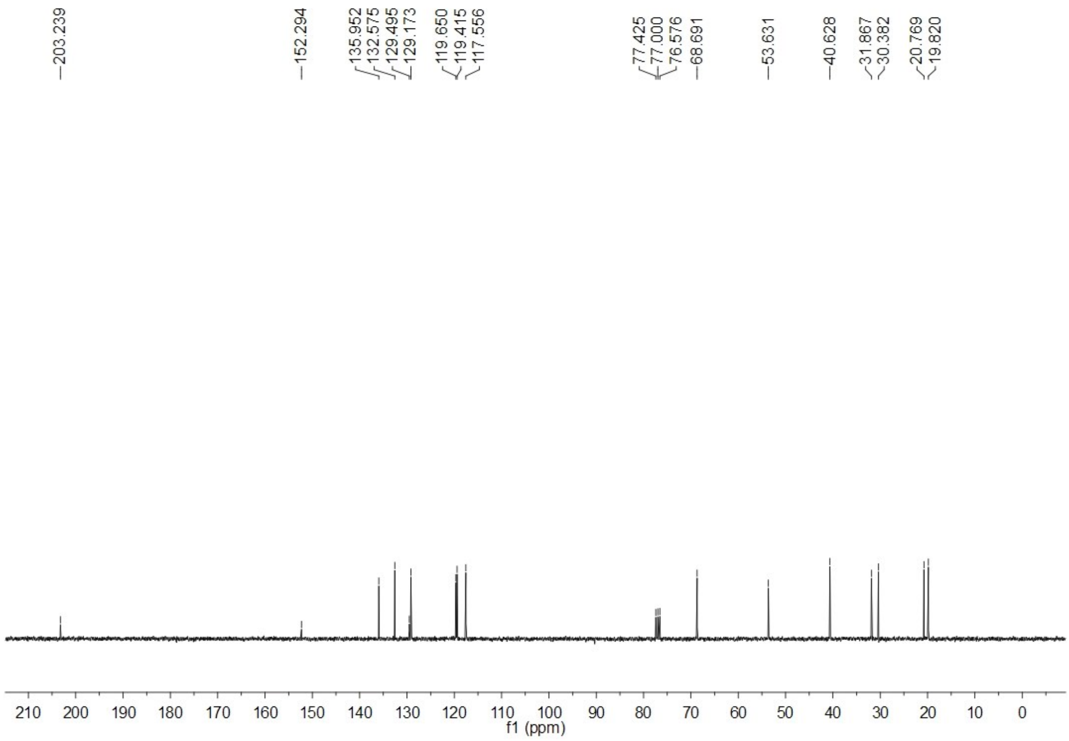

NMR spectrum of Compound **3d**

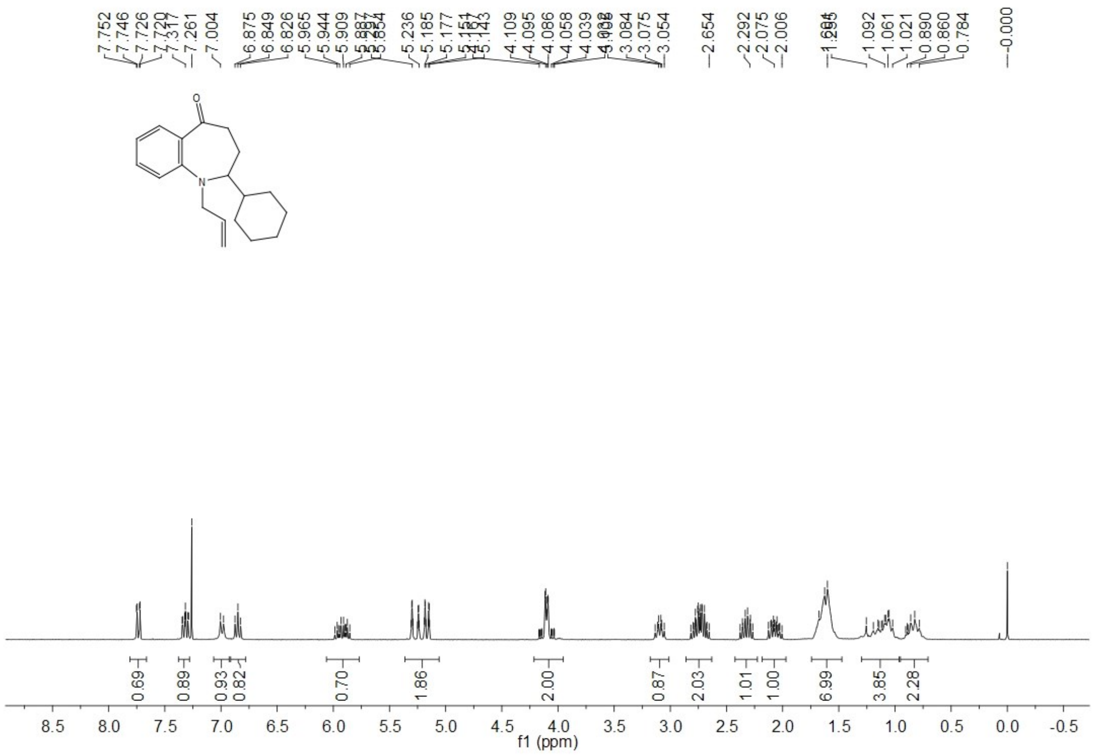

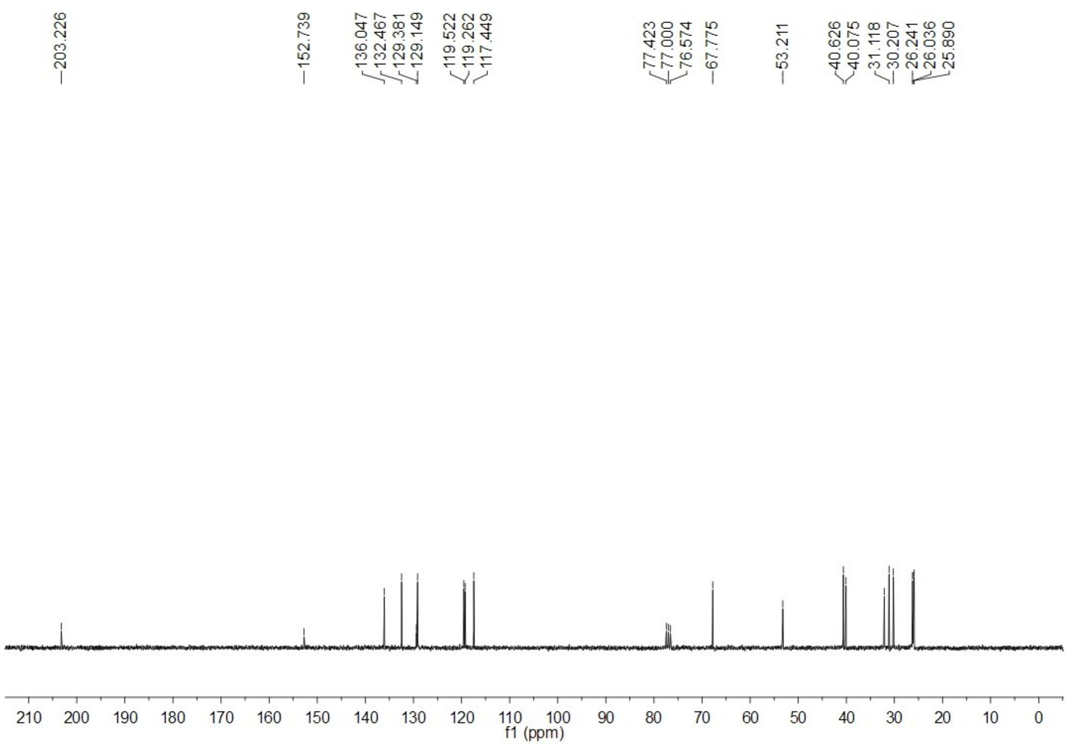

NMR spectrum of Compound **3e**

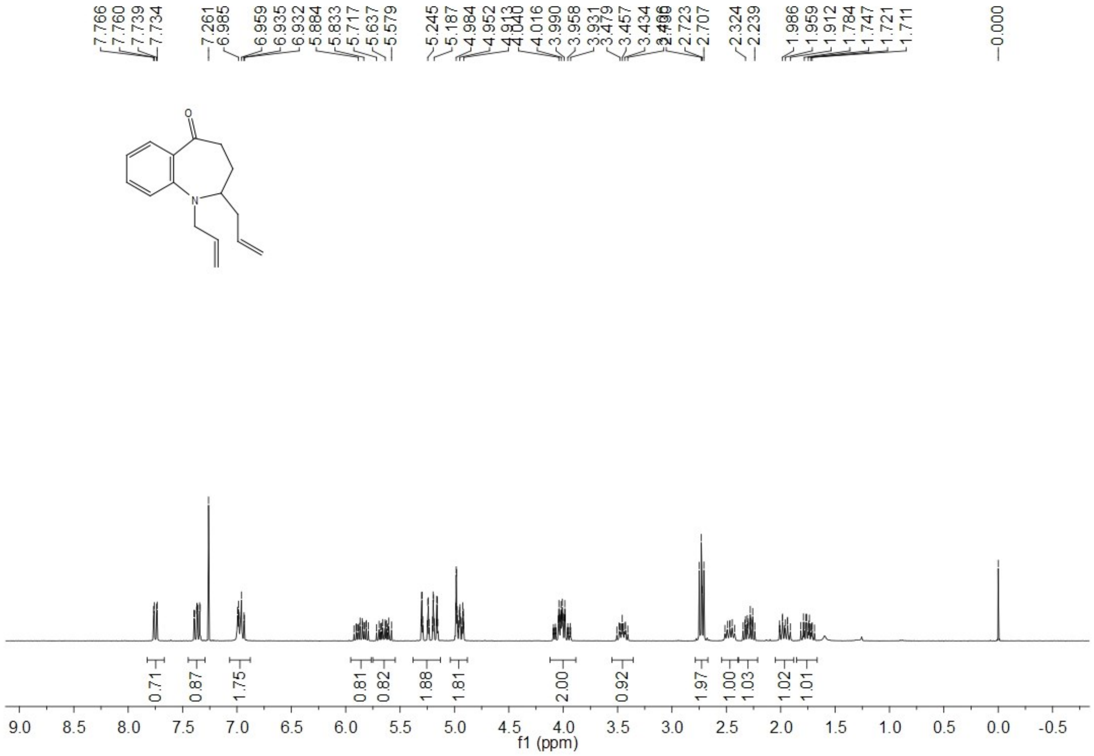

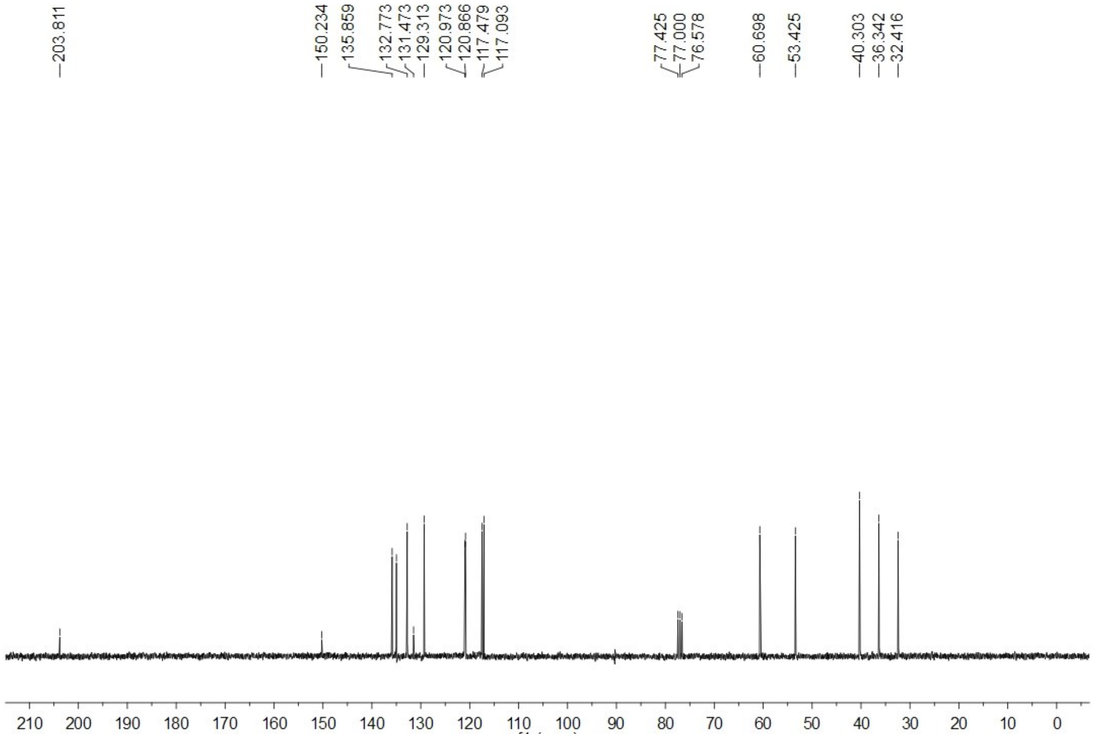

NMR spectrum of Compound **3f**

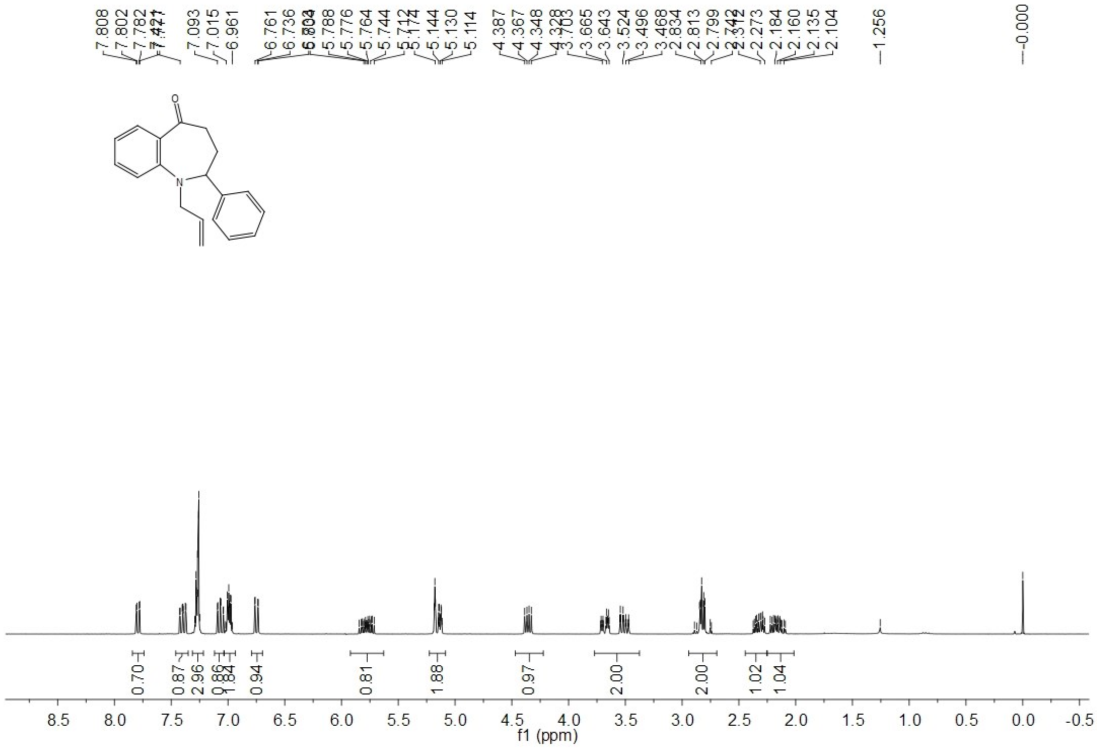

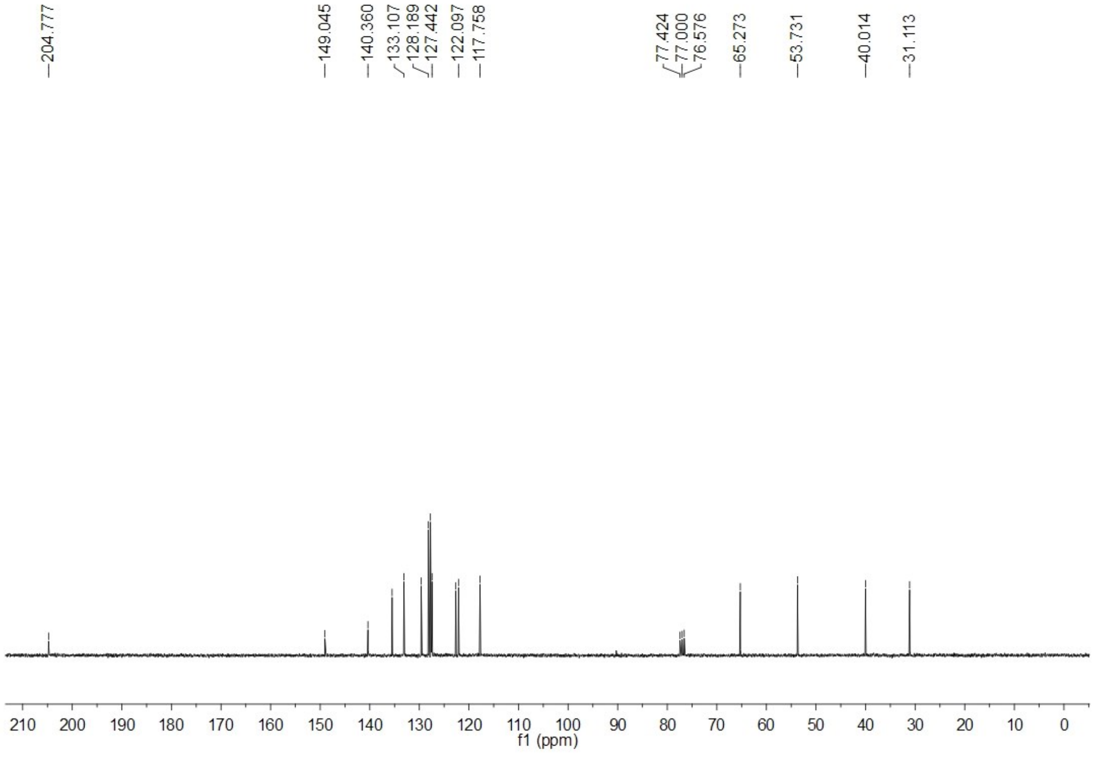

NMR spectrum of Compound **3g**

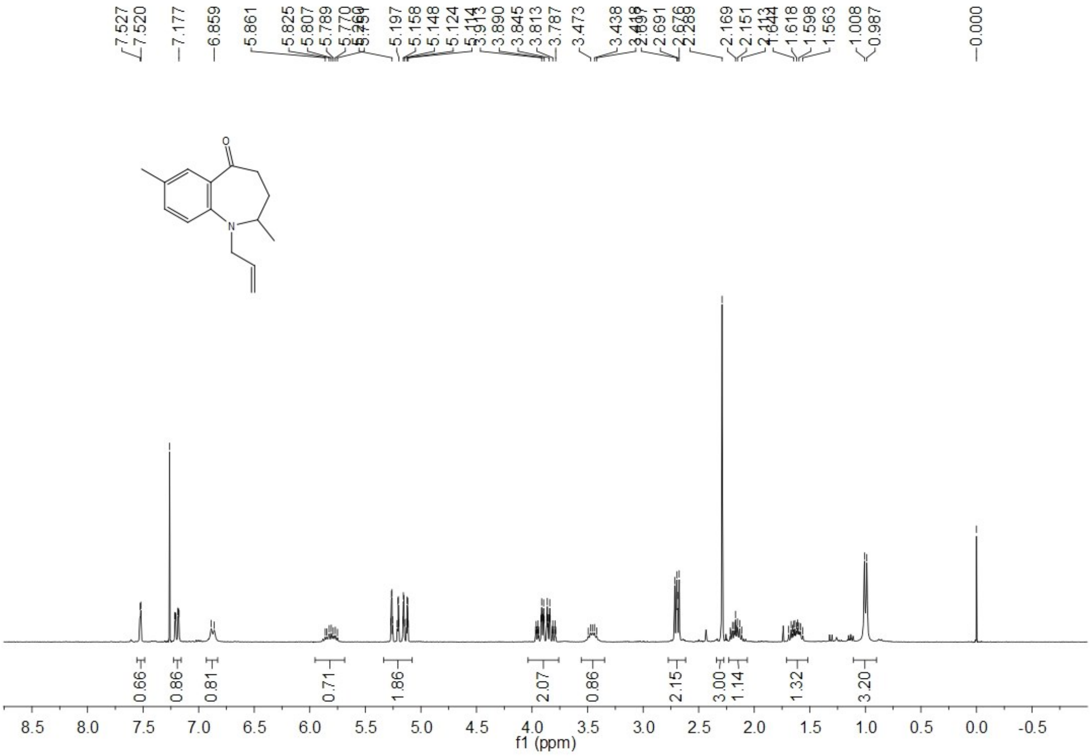

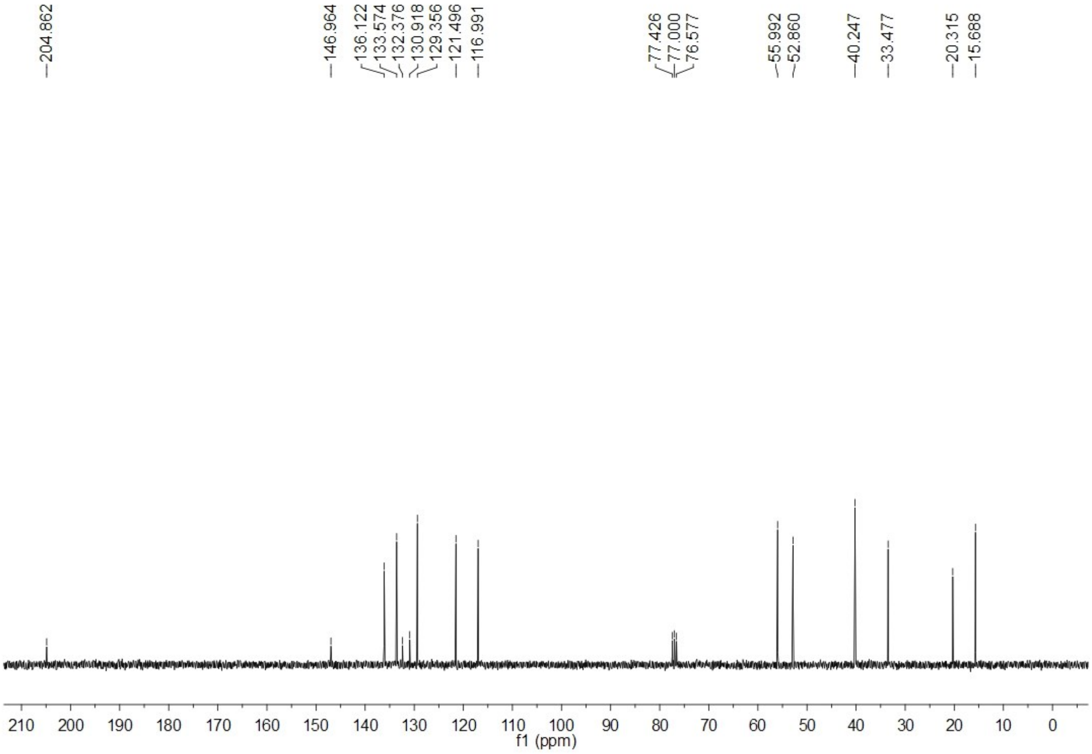

NMR spectrum of Compound **3h**

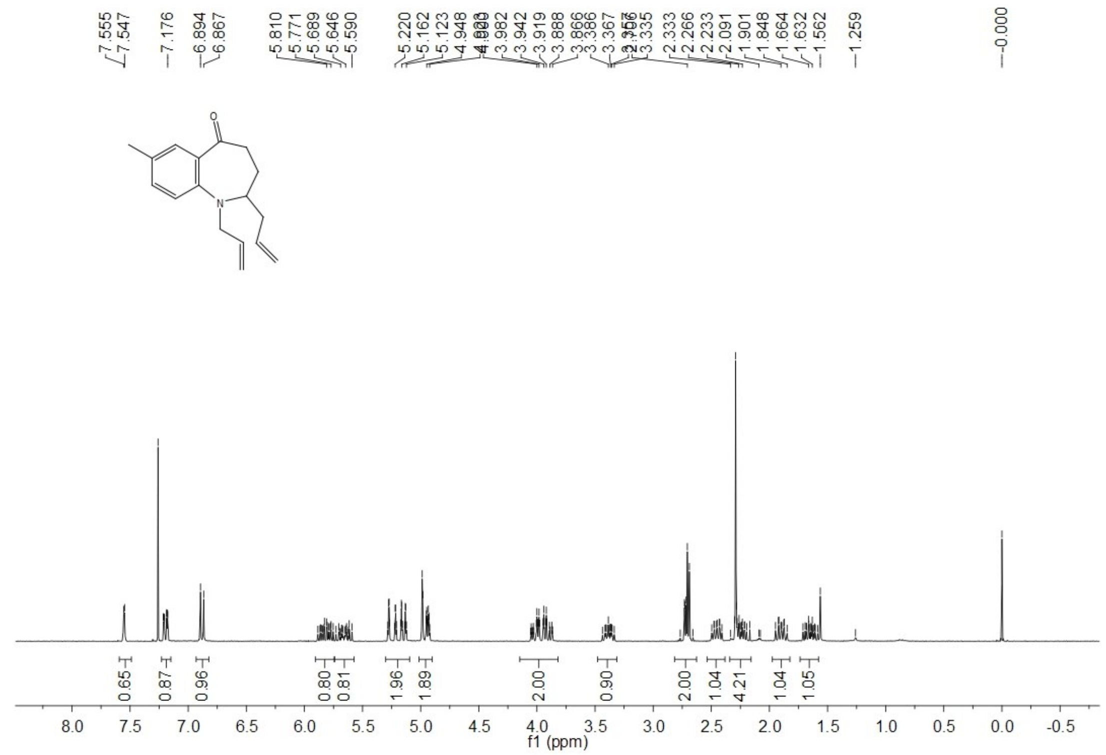

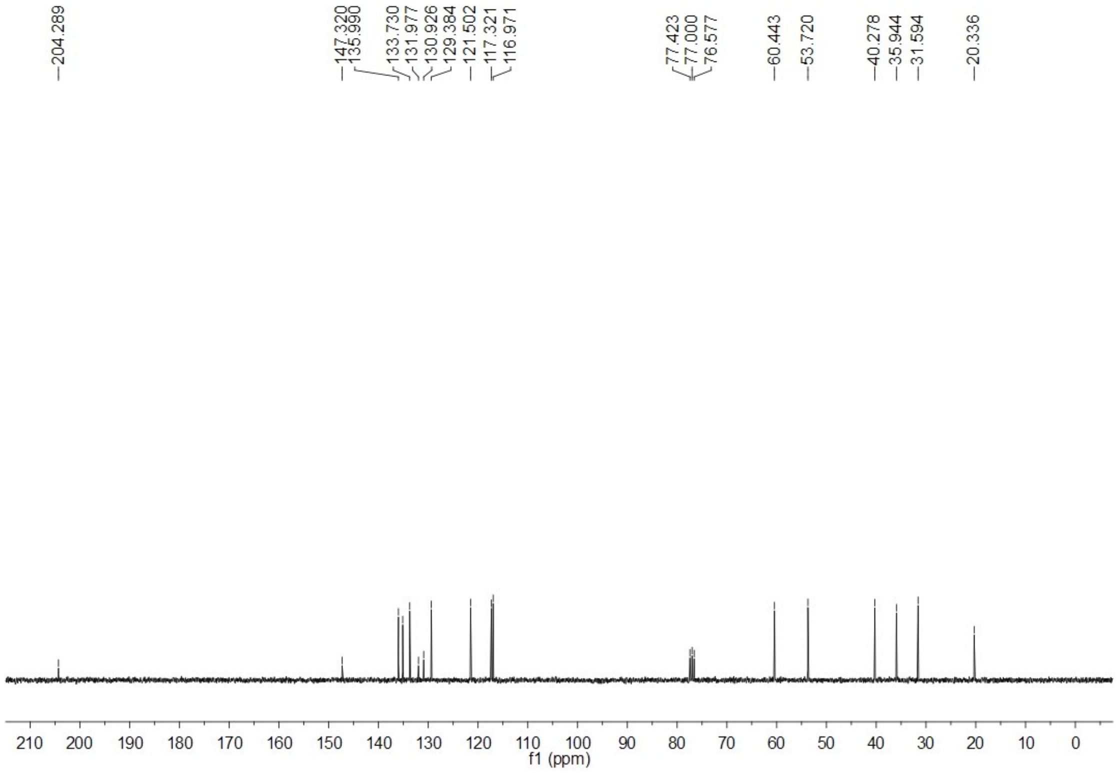

NMR spectrum of Compound **3i**

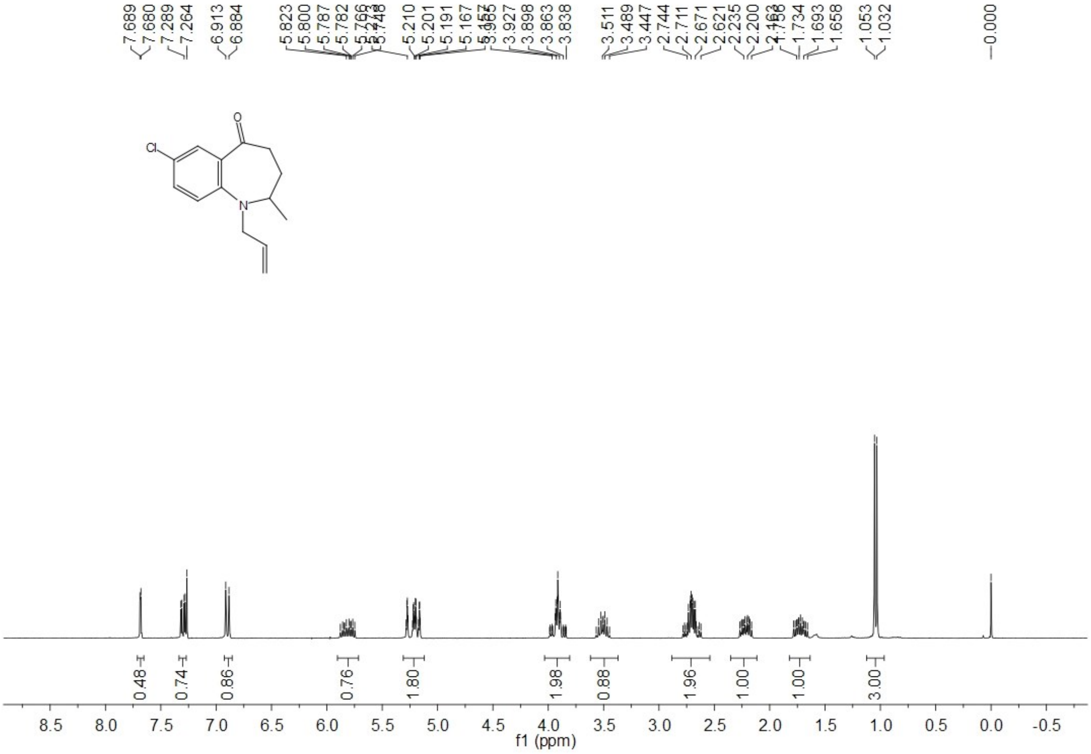

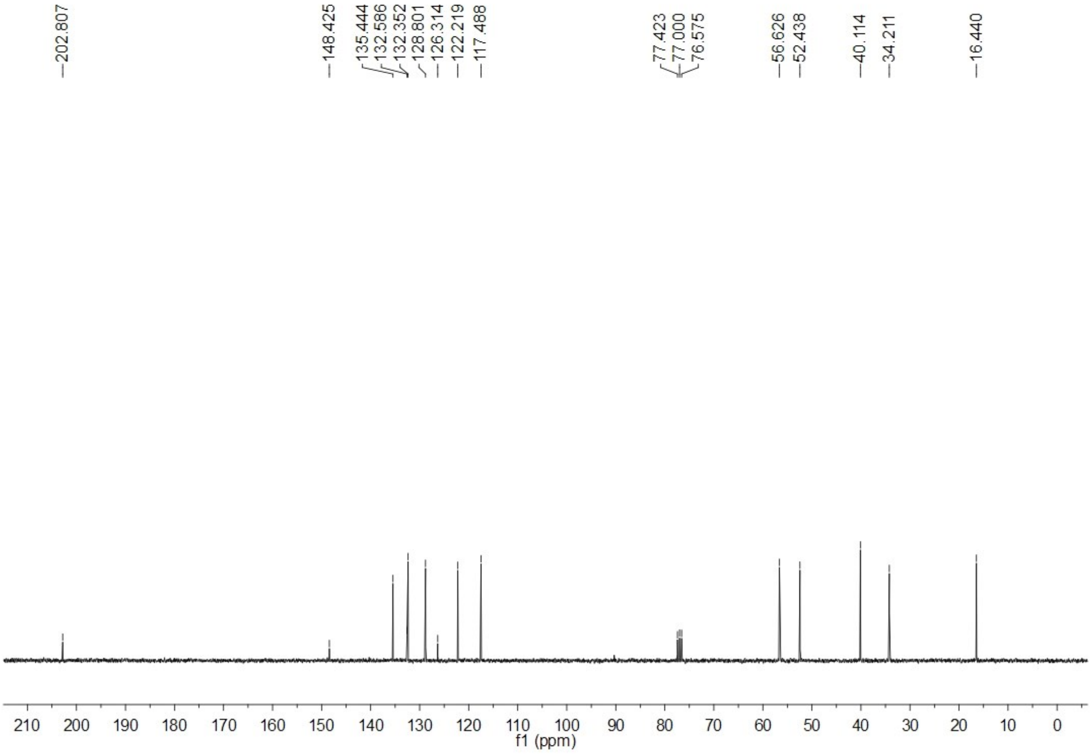

NMR spectrum of Compound **3j**

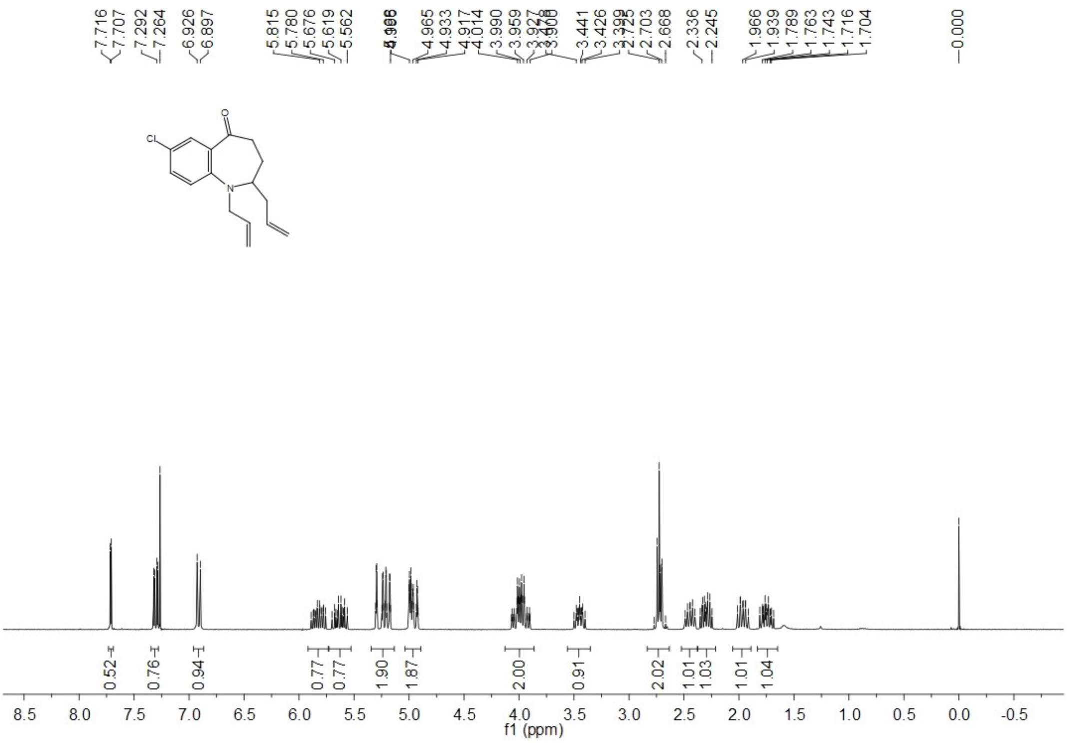

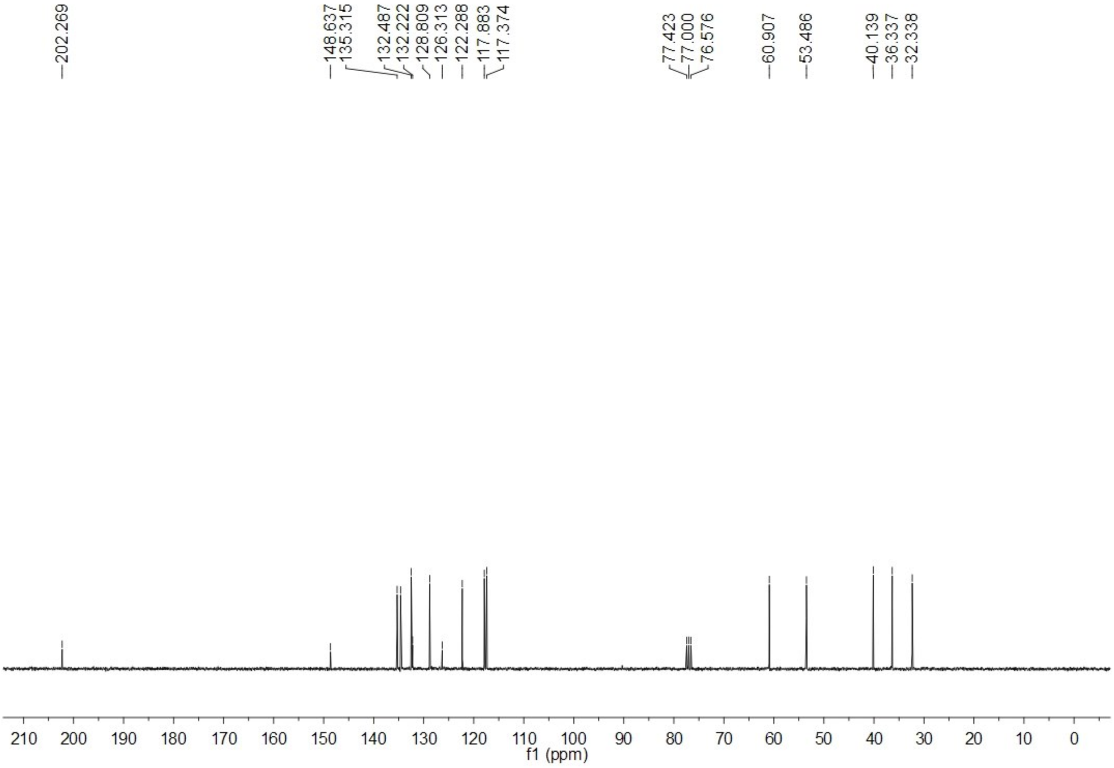

NMR spectrum of Compound **3k**

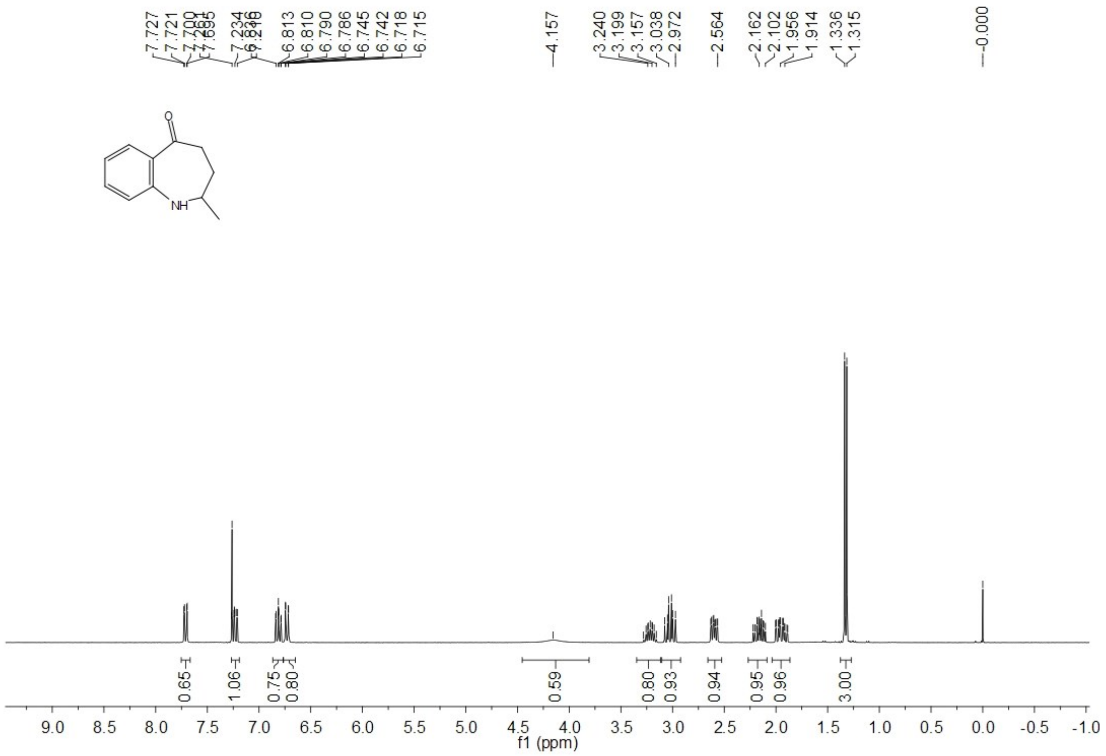

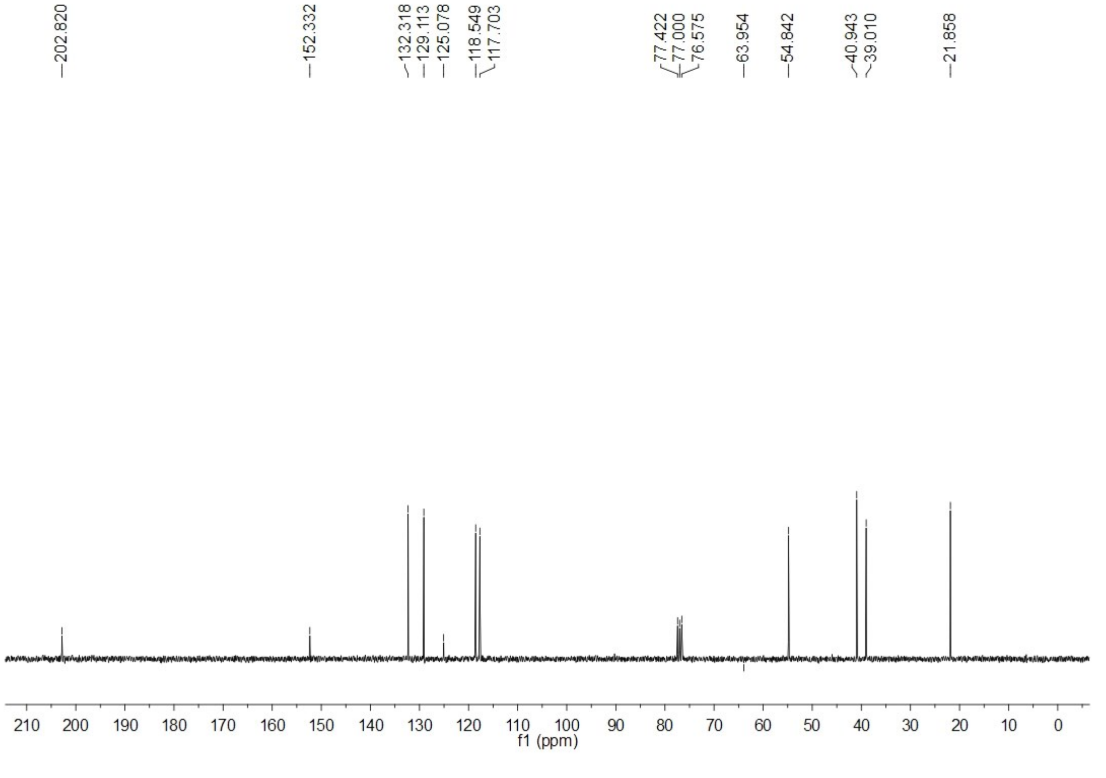

NMR spectrum of Compound **4**

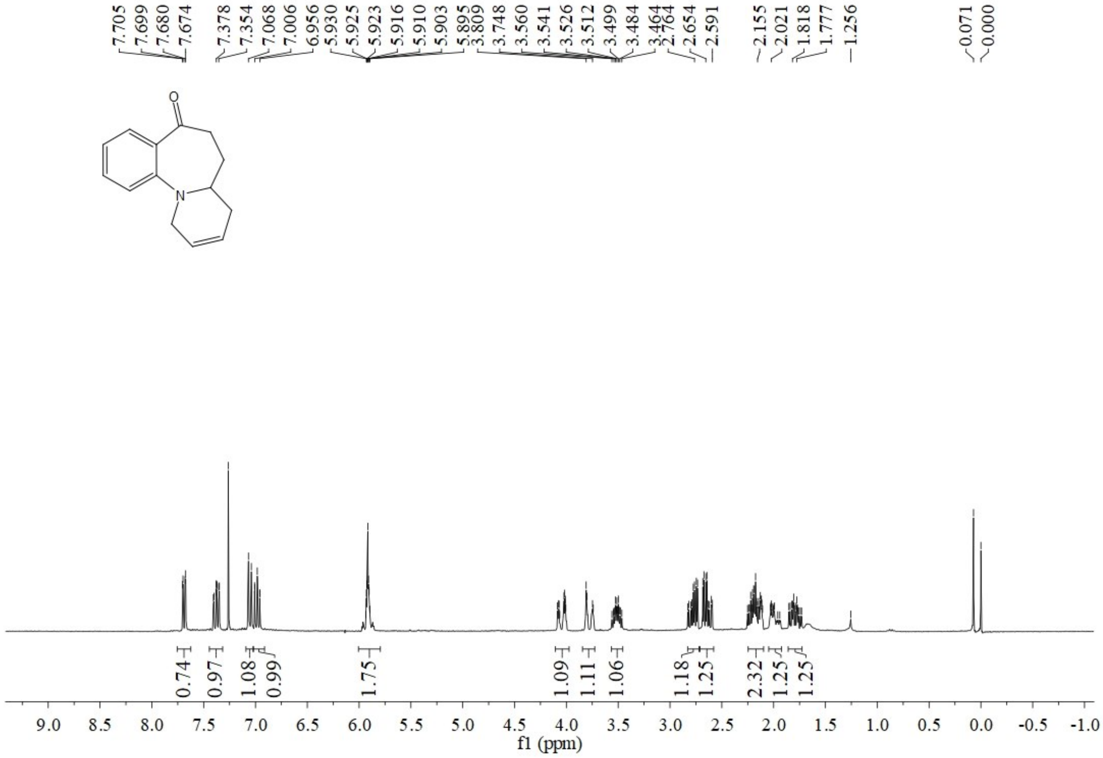

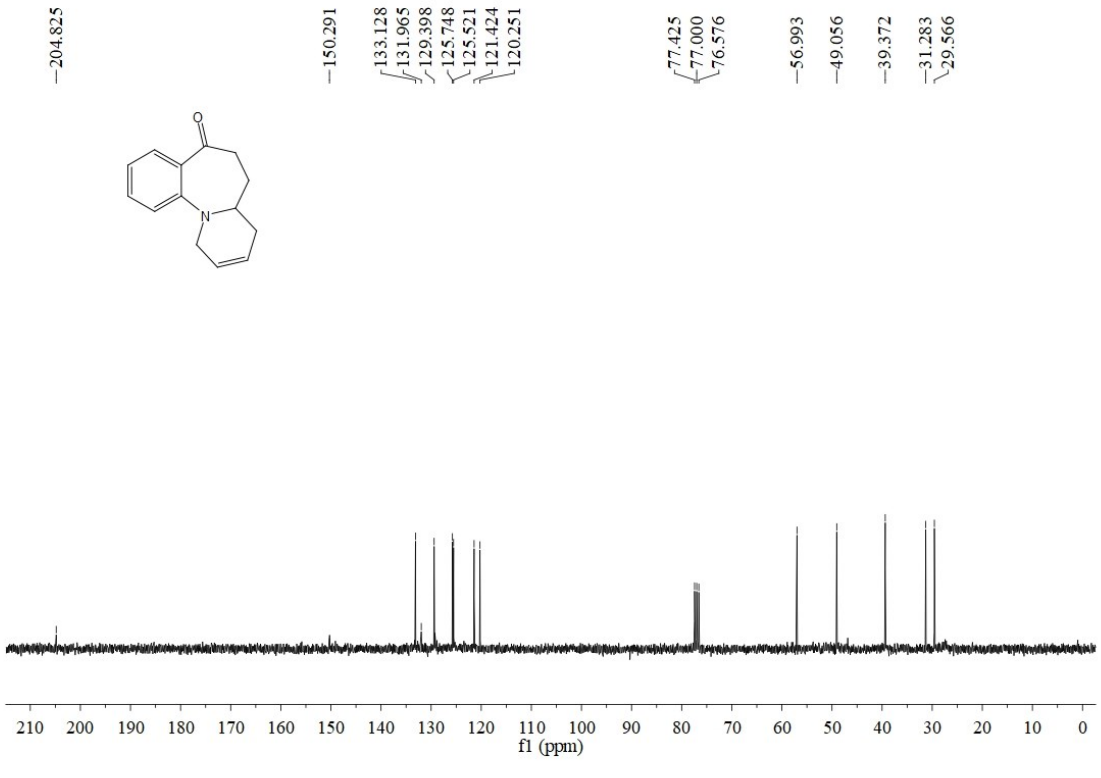

X-ray Crystallographic data for Compound syn-2m

The crystallographic data of syn-**2m** (CCDC 1033762) can be obtained free of charge from The Cambridge Crystallographic Data Centre via www.ccdc.cam.ac.uk.

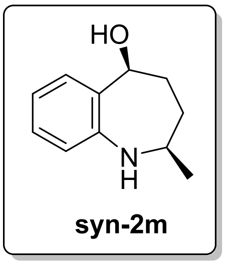

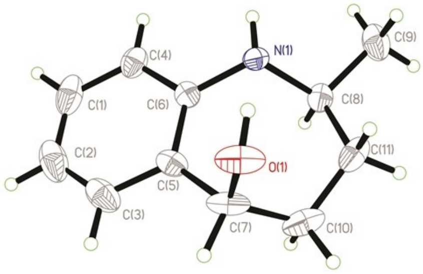

Confirmation of the ratio of stereoisomers 2

Through the analysis of ^{1}H NMR spectra, the chemical shifts of hydrogen on C1 of different isomers are significantly different. The diastereomeric ratio of compound **2m** were calculated by ^{1}H NMR spectroscopy (by comparison of the chemical shifts of the proton H_1 at 4.81 ppm (anti-**2m**) and 4.76 ppm (syn-**2m**)). In the same way, the diastereomeric ratio of compound **2n** can also be calculated (S-Table 1).

S-Table 1 Confirmation of the diastereomeric ratio of compound **2m** and **2n**

Entry	structure	*H* (C1)	isomer 1/isomer 2	syn/anti
1	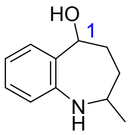 **2m**	isomer 1: 4.81 (dd, J = 8.7, 1.8 Hz)<br>isomer 2: 4.76 (d, J = 6.3 Hz)	1:10	91/9
2	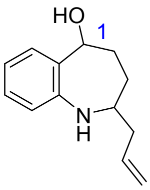 **2n**	isomer 1:4.81 (dd, J = 8.7, 1.8 Hz)<br>isomer 2: 4.77 (d, J = 6.6 Hz)	1:3	75/25

The ^{1}H NMR spectrums of the compounds with a substituent attached to the nitrogen atom were quite different from compound **2m**, so the diastereomeric ratio could not be calculated by direct comparison. Therefore, we established the correlation between these two types of compounds using chemical method. Treatment of **2a** and **2e** with $Pd(OAc)_2$ and *N,N*-dimethylbarbituricacid (NDMBA) proceeded to give **2m** and **2n**, without affecting the chirality of the substrate[1-9]. Then, the diastereomeric ratio of **2a** and **2e** could be determined (S-Fig. 1 and S-Fig. 2).

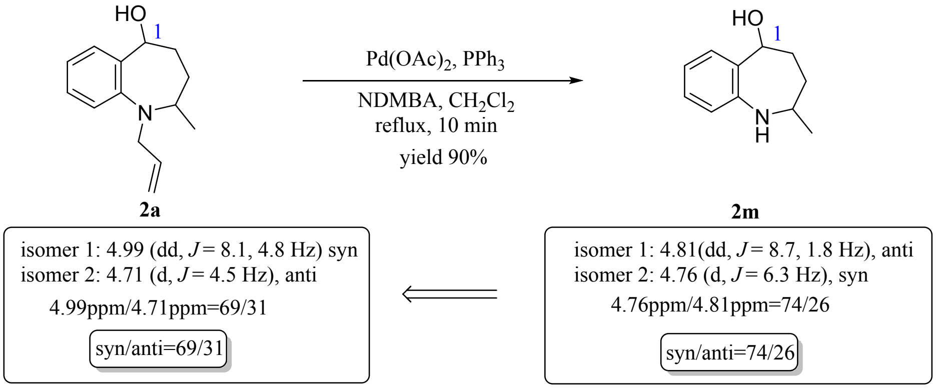

S-Fig.1 Correlation between compound **2a** and **2m**

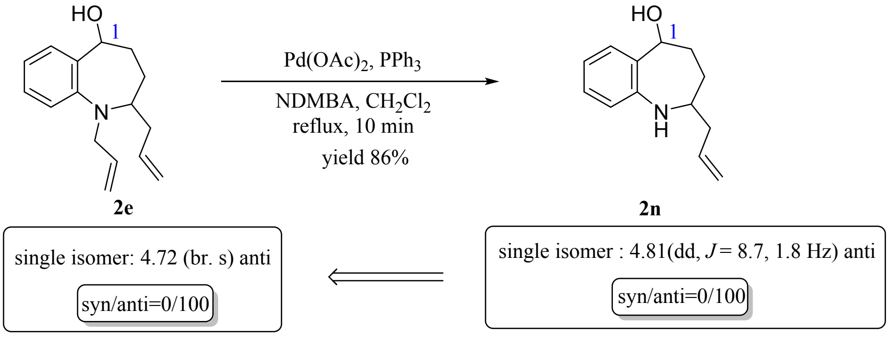

S-Fig.2 Correlation between compound **2e** and **2n**

The stereochemistry in the rest of the series could be unambiguously assigned by comparison of their NMR spectra with those of **2a** and **2e** (S-Table 2).

S-Table 2 Confirmation of the diastereomeric ratio of compound **2a-l**

entry	structure	*H* (C1)	isomer 1/ isomer 2	syn/anti
1	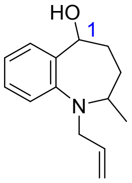 **2a**	isomer 1: 4.99 (dd, J = 8.1, 4.8 Hz)<br>isomer 2: 4.71 (t, J = 4.5 Hz)	2.22:1	69/31
2	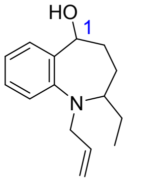 **2b**	isomer 1: 4.89 (dd, J =9.6, 3.6 Hz)<br>isomer 2: 4.71 (br. s)	10:1	91/9
3	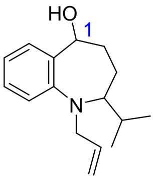 **2c**	4.83 (dd, J = 10.2, 3.6 Hz, 1H).	single	100/0
4	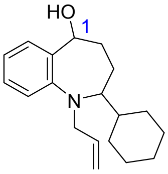 **2** **d**	4.82 (dd, J = 10.2, 3.3 Hz)	single	100/0
5	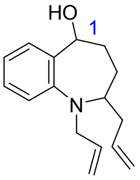 **2e**	4.72 (br. s, 1H)	single	0/100
6	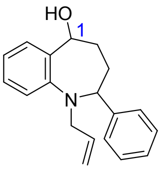 **2f**	5.36 (t, J = 8.1 Hz, 1H)	single	100/0

7	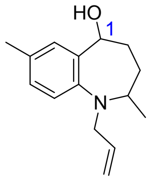<br>**2g**	isomer 1: 4.98 (dd, J = 7.8, 4.8 Hz)<br>isomer 2: 4.64 (t, J = 4.5 Hz)	7.69:1	88/12
8	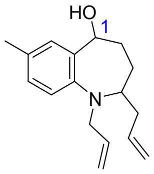<br>**2h**	4.67 (br. s)	single	0/100
9	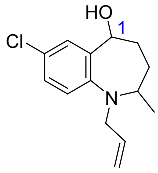<br>**2i**	isomer 1: 4.96 (dd, J = 8.7, 4.5 Hz)<br>isomer 2: 4.68 (br. s)	2.04:1	67/33
10	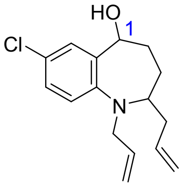<br>**2j**	4.69 (br. s)	single	0/100
11	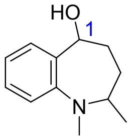<br>**2k**	isomer 1: 4.80 (t, J = 4.5 Hz)<br>isomer 2: 4.77 (d)	2.13:1	68/32
12	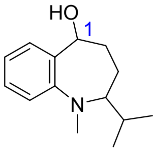<br>**2l**	4.80 (dd, J = 9.9, 4.2 Hz)	single	100/0

References:

1. F. Garro-Helion, A. Merzouk and F. Guibe, *J. Org. Chem.*, 1993, **58**, 6109-6113.

2. S. Lemaire-Audoire, M. Savignac, J. P. Genêt and J.-M. Bernard, *Tetrahedron Lett.,* 1995, **36**, 1267-1270.

3. Z. Ling, L. Yun, L. Liu, B. Wu and X. Fu, *Chem. Commun.*, 2013, **49**, 4214-4216.

4. S. G. Davies, A. M. Fletcher, J. A. Lee, T. J. A. Lorkin, P. M. Roberts and J. E. Thomson, *Tetrahedron*, 2013, **69**, 9779-9803.

5. S. Norsikian, J.-F. Soulé, A. Cannillo, R. Guillot, M.-E. Tran Huu Dau and J.-M. Beau, *Org. Lett.*, 2012, **14**, 544-547.

6. S. G. Davies, J. R. Haggitt, O. Ichihara, R. J. Kelly, M. A. Leech, A. J. Price Mortimer, P. M. Roberts and A. D. Smith, *Org. Biomol. Chem.,* 2004, **2**, 2630-2649.

7. S. Lou and S. E. Schaus, *J. Am. Chem. Soc.*, 2008, **130**, 6922-6923.

8. J. I. Candela-Lena, S. G. Davies, P. M. Roberts, B. Roux, A. J. Russell, E. M. Sánchez-Fernández and A. D. Smith, *Tetrahedron: Asymmetry*, 2006, **17**, 1135-1145.

9. S. G. Davies, A. M. Fletcher, J. A. Lee, T. J. A. Lorkin, P. M. Roberts and J. E. Thomson, *Org. Lett.,* 2013, **15**, 2050-2053.
